# Updated Perceptions on Polymer-Based Enhanced Oil Recovery toward High-Temperature High-Salinity Tolerance for Successful Field Applications in Carbonate Reservoirs

**DOI:** 10.3390/polym14102001

**Published:** 2022-05-13

**Authors:** Anas M. Hassan, Emad W. Al-Shalabi, Mohammed A. Ayoub

**Affiliations:** 1 Petroleum Engineering Department, Khalifa University of Science and Technology, Abu Dhabi 127788, United Arab Emirates; emad.walshalabi@ku.ac.ae; 2 Petroleum Engineering Department, Universiti Teknologi PETRONAS (UTP), Seri Iskandar 32610, Malaysia; abdalla.ayoub@utp.edu.my

**Keywords:** enhanced oil recovery (EOR), polymer flooding, high temperature high salinity (HTHS), field applications, carbonate reservoirs

## Abstract

The aging of the existing reservoirs makes the hydrocarbon extraction shift toward newer reserves, and harsh conditioned carbonates, which possess high temperature and high salinity (HTHS). Conventional polymer-flooding fails in these HTHS carbonates, due to precipitation, viscosity loss, and polymer adsorption. Therefore, to counteract these challenges, novel polymer-based cEOR alternatives employ optimized polymers, polymer–surfactant, and alkali–surfactant–polymer solutions along with hybrid methods, which have shown a potential to target the residual or remaining oils in carbonates. Consequently, we investigate novel polymers, viz., ATBS, Scleroglucan, NVP-based polymers, and hydrophobic associative polymers, along with bio-polymers. These selected polymers have shown low shear sensitivity, low adsorption, and robust thermal/salinity tolerance. Additionally, adding an alkali-surfactant to polymer solution produces a synergy effect of improved mobility control, wettability alteration, and interfacial-tension reduction. Thus, enhancing the displacement and sweep efficiencies. Moreover, low-salinity water can precondition high-salinity reservoirs before polymer flooding (hybrid method), to decrease polymer adsorption and viscosity loss. Thus, this paper is a reference for novel polymers, and their hybrid techniques, to improve polymer-based cEOR field applications under HTHS conditions in carbonates. Additionally, the recommendations can assist in project designs with reasonable costs and minimal environmental impact. The implication of this work will aid in supplementing the oil and gas energy sector growth, making a positive contribution to the Middle Eastern economy.

## 1. Introduction

The demand for and supply of hydrocarbon (fossil fuel) continues to be significant and contributes in the order of 85% to the total energy mix. A part is contributed by crude oil. According to the International Energy Agency, (IEA, 2020), crude oil is expected to contribute approximately 30% of the world’s energy supply by 2035 [1,2,3].

The growing energy demand worldwide has pushed oil extraction to venture toward newer hydrocarbon energy resources, as most existing reservoirs reach maturity. A large portion of the new reserves are to be found in carbonate reservoirs [4,5,6], where the reservoir environment is marked by heterogeneity, high temperature and high salinity (HTHS) or harsh conditions [7,8]. Consequently, improved and enhanced oil recovery (IOR/EOR) techniques can be a powerful tool to unlock a significant amount of both unswept oil as well as capillary rapped oil saturations from the existing and new (i.e., harsh carbonates) hydrocarbon reservoirs.

Traditionally, IOR/EOR refers to oil recovery by injecting materials not naturally present in the reservoir [9]. This definition embraces all methods of the oil recovery process (e.g., drive, push–pull, and well treatments) and covers many oil recovery agents [9]. Most importantly, the definition does not restrict or limit IOR/EOR to a particular production stage (e.g., secondary and tertiary) in the production life of the reservoir [9]. Therefore, in this contribution, the terminology of EOR is used as a representative for IOR/EOR, irrespective of whether it is secondary or tertiary. Primary recovery refers to the process of extracting hydrocarbons through natural drive (i.e., natural depletion) mechanisms, such as water influx, solution gas, and gas cap drive or gravity drainage [10]. Secondary recovery involves the injection of water and gas whose purpose of maintaining reservoir pressure. Tertiary recovery, is any technique applied after secondary displacements, such as gas-EOR flooding, water-based EOR injection (i.e., low salinity), chemical flooding (i.e., polymer, surfactant, and alkaline surfactant polymer or ASP [9,11]), and hybrid-EOR (e.g., low salinity polymer flooding [12] and smart-water assisted foam flooding or SWAF Technology [13,14,15,16,17,18]).

**Figure 1:**
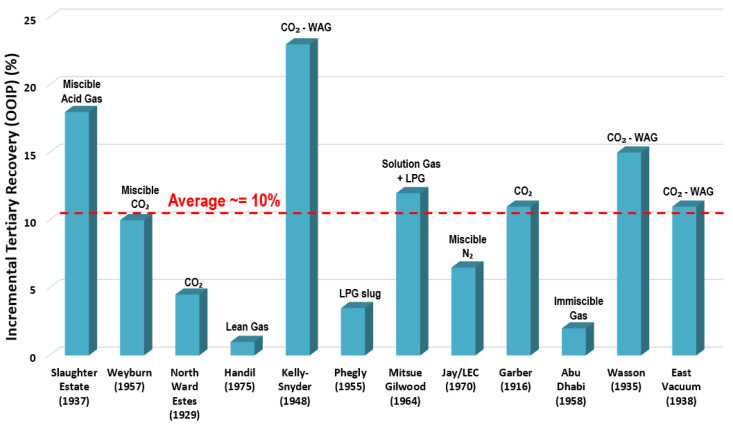
Incremental tertiary recoveries by gas-injection in selection successful EOR projects; the dates in the x-axis indicate the discovery years of the fields.

The technique of gas flooding involves injections of either nitrogen (N${}_{2}$) or carbon dioxide (CO${}_{2}$) gases, which solubilize with crude oil to reduce the oil viscosity and displace it easily, thus, the oil mobility improves. Figure 1 shows that the incremental tertiary recoveries by gas-EOR injection in current successful projects is 10% (i.e., original oil in place or OOIP) [19].

Regarding environmental impact, N${}_{2}$ gas derived from air is generally considered more environmentally acceptable than CO${}_{2}$ gas. Nonetheless, CO${}_{2}$ sequestration through the carbon capture, utilization, and storage (CCUS) technology, can be advantageous in CO${}_{2}$ emission cuts. Furthermore, to decarbonize energy sources, hydrogen as an energy carrier can be a retrofitted with CCU [20], which has zero-carbon emission (hydrogen with 141.86 MJ/kg energy density) during combustion, and can be sustainably stored in depleted reservoirs [21].

Moreover, the principal role of the EOR process is in displacing oil in the production wells to supplement the natural energy present in the reservoir using injection of different fluids. These injecting fluids transform the reservoir properties; for instance, they can lower the interfacial tension (IFT) between oil and water, modify the rock wettability, cause alteration in the pH value, form emulsions, assist in clay migration, and decrease the oil viscosity [22].

One of the promising water-based EOR techniques is low salinity water (LSW) flooding. Since its introduction, over 50 years ago in the 1960s [23,24], LSW flooding has been applied and considered an effective technique for improved oil recovery. This technique’s effects decrease and optimize the water chemistry, i.e., ion type, concentration, and salinity [25].

It is proven that injected LSW, which is unlike the composition and salinity of the initial formation water, i.e., connate water, can disrupt the pre-existing chemical equilibrium of the COBR system through the course of creating a new chemical equilibrium [26,27,28]. In addition, the wetting properties, which strongly affect the two-phase fluid flow, are altered because of the capillary pressure (P${}_{c}$). Moreover, the relative permeability of water and oil (K${}_{w}$ and K${}_{o}$) shifts, which may cause desirable improved oil recovery in both clastic and carbonate formations. In addition, a methodical laboratory investigation by Morrow et al. [29] resulted in improved optimal oil recovery using LSW flooding when the COBR system was slightly water wet [30]. In the sandstone reservoirs, the execution of LSW flooding either as a secondary or tertiary oil recovery method to accelerate oil production and to reduce residual and remaining oil in the formation, was expansively studied by numerous investigators and research-groups [24,28,31].

Furthermore, one of the main EOR techniques is chemical enhanced oil recovery (cEOR) that is applied in about 11% of EOR projects worldwide [32]. These cEOR methods usually target both unswept oil as well as capillary rapped oil saturations [9]. Chemical EOR (cEOR) is a procedure in which one or more chemicals are first screened and selected and then added to water, which is injected into the reservoir to improve the oil recovery factor over the levels achieved by water flooding [22]. Polymers, surfactants, and alkalis are the most frequently used chemicals in cEOR, which can enhance the water/oil and gas/oil interfacial tension (IFT), microscopic and macroscopic displacement efficiencies, and water–oil bedside viscosities. Additionally, they can modify the rock wettability and cause reduction in the water-cut and water/oil ratio [22]. These modifications of properties of a rock/fluid (i.e., crude oil–brine–rock or COBR) system through EOR include changing the mobility ratio or the capillary number. In the case of polymers, they are employed to improve the macroscopic displacement efficiency by lowering the mobility ratio (i.e., polymers provide mobility control [33,34], whereas surfactants are used to improve the macroscopic displacement efficiency by increasing the capillary number (i.e., surfactants reduce the capillary forces) [22,35].

**Figure 2:**
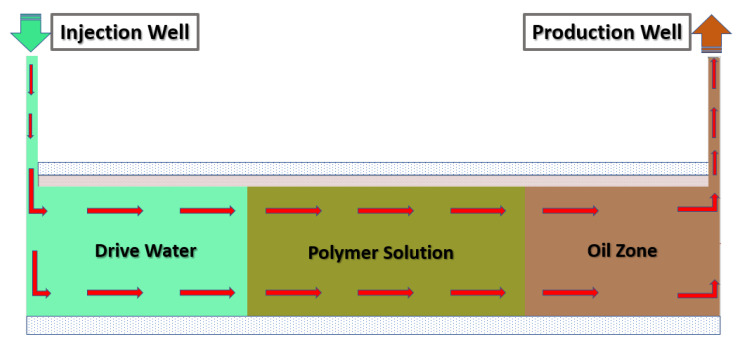
Illustration of a typical polymer-based chemical-enhanced oil recovery (cEOR) procedure.

Polymer flooding (PF) is the most common of these cEOR methods, accounting for 77% of worldwide projects, and the remaining 23% CEOR applications are polymers combined with surfactants and alkali for polymer–surfactant flooding (PSF) and polymer–surfactant–alkaline flooding (PSAF), respectively [36]. Polymer flooding (PF) is a cEOR technology, in which a highly viscous polymer solution is injected into the formation to sufficiently reduce the fluidity ratio and expand the swept volume, thereby improving the oil recovery, as shown in Figure 2 [37].

**Figure 3:**
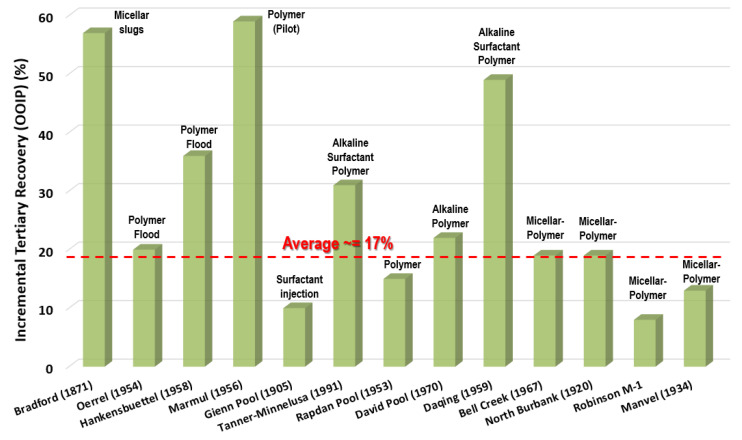
Incremental tertiary recoveries by chemical injection in selection of successful EOR projects; the dates in the x-axis indicate the discovery years of the fields.

Unlike water, polymer solutions display non-Newtonian fluid behavior, i.e., either shear thinning or shear thickening as a function of the shear rate [38,39]. Apart from the viscosification behavior, polymers have unique characteristics, such as toughness and viscoelasticity, owing to their long chain structure and high molecular weight. These features enable them to stretch oil droplets and oil film during flow, resulting in a higher carrying capacity [40]. Figure 3 shows that the incremental tertiary recoveries by chemical-based EOR (i.e., cEOR) injection in current successful projects is 30% (OOIP) [19].

Polymer behaviors correspond and alter with temperature and salinity as well as permeability and heterogeneity variations. Nonetheless, polymers (i.e., in low salinity aqueous solutions) can alter the rock wettability and reduce the water cut and water/oil ratio. When this alteration is permanent on the rock surface, other cEOR processes cannot be carried out [22,41]. The two categories of polymers most extensively used in polymer-based cEOR are synthetic polymers and biopolymers [42]. The synthetic polymer, called hydrolyzed polyacrylamide (HPAM), is the most commonly applied, as it has the benefits of low cost and high molecular weight. Additionally, Xanthan, a non-ionic bio-polymer is another important polymer used in cEOR [32]. The general expectation from the polymer-based cEOR technique is 50% final recovery with 15% to 20% improved recovery beyond water flooding [43].

According to Akbar et al. [6], 60% of oil reserves globally are concentrated in fractured carbonate reservoirs, many of which have harsh conditions (i.e., HTHS) [6]. Therefore, extending the application of polymer-based cEOR to HTHS carbonate reservoirs to successfully optimize production will significantly help in the sustainable growth of the oil industry. Thus, the development of appropriate polymers able to maintain their functionality in harsh conditions, such as those encountered in Middle East carbonate reservoirs (temperature higher than 100 ${}^{{}^{\circ}}$C and salinity up to 280 g/L), is essential to sustain the oil industry by unlocking a lot of reserves [44]. With this purpose, numerous experiments were conducted on novel polymers to evaluate their stability in HTHS-conditioned carbonates. These include, but are not limited to, the new ATBS polymers (with a high degree of polymerization), scleroglucan, NVP-based polymers, and hydrophobic associative polymers, smart thermoviscosifying polymers (TVP), salt-induced TVPs, soft microgel polymers (SMG), sulfonated polymers, and HPAM modifications. For instance, the new smart TVP exhibited a strong salt-induced viscosifying property, which is influenced by temperature, shear rate, and polymer concentration. The viscosifying ability due to salt is decreased with the increase in temperature and shear, whereas it is increased with the increase in concentration of polymers [45]. Consequently, this review paper analyzes the data from these investigations and connects them with most relevant literature to derive positive conclusions for the successful extension of polymer-based cEOR to HTHS carbonate reservoirs.

**Figure 4:**
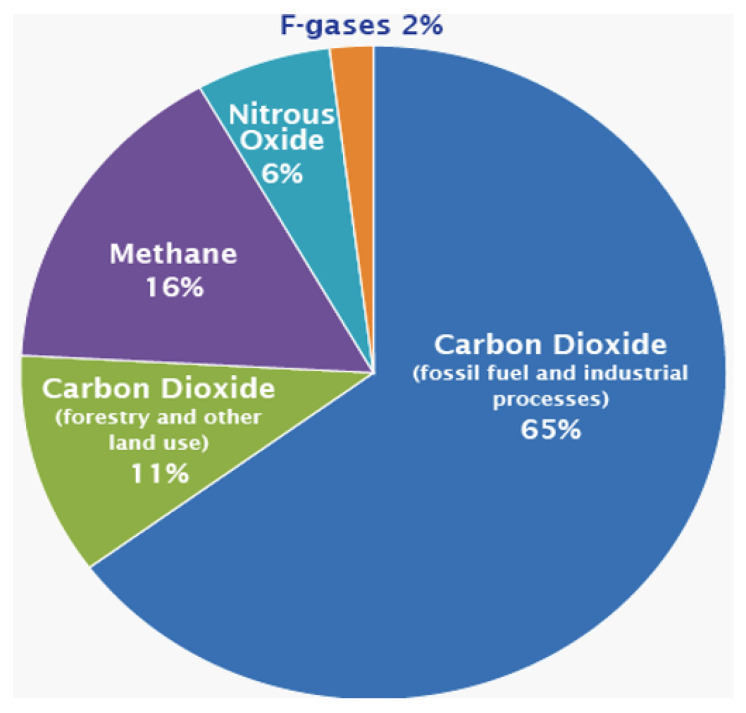
Global greenhouse gas emissions, based on data from global emissions from 2010 (Data from IPCC, 2014 [46]).

Additionally, climate change has become a global concern of our time. Therefore, limiting CO${}_{2}$ emissions (CO${}_{2}$ footprint) associated with polymer-based cEOR may become the primary screening criteria for polymers apart from other parameters. Moreover, in 2015, global efforts were launched in the Paris Agreement to lower CO${}_{2}$ emission risks which affect climate change by controlling the global average temperatures, i.e., attaining 1.5 ${}^{{}^{\circ}}$C average temperature, and to achieve net-zero emissions by the 2050s [46]. However, the current statistics show that the atmospheric CO${}_{2}$ concentration is rising steadily as depicted in the pie chart in Figure 4. Therefore, in order to effectively avoid the climate change effects, several large-scale solutions are necessary [21], which include decarbonization through solar and wind renewable energy, and hydrogen produced through electrolysis from renewable energy or via hydrocarbon reformation, which has been retrofitted with carbon capture and storage (CCS) [20], as a viable low-carbon energy carrier, among other solutions. Hydrogen can potentially substitute the natural gas used for domestic heating and power generation, decarbonize transport facilities, enable increased renewable energy power production by providing an energy store to stabilize supply and demand, and offer a sustainable energy storage solution [47,48].

Furthermore, even the hydrogen created from fossil fuels via conventional methods can act as an energy carrier, and facilitate the carbon capture process by practically transmitting the carbon emission source from users to power plants. To enable hydrogen as a low-carbon energy pathway, inter-seasonal [49] or longer-term TWh storage solutions (e.g., 150 TWh [50] for the UK seasonal energy storage will be required, which can be addressed by storage in suitable geological formations. It is here that the matured and depleted gas/oil fields can be utilized for geological storage of hydrogen (GSH), as there are numerous depleted reservoirs and a large operational dataset to ease the hydrogen storage aptness evaluations. Additionally, the offshore geological storage of hydrogen (OGSH), in deep sea sites, may be the more suitable choice for the application of hydrogen storage, rather than onshore sites. This way, through the repurposing of present gas/oil fields is expected to lessen the appraisal and development costs of storage sites while minimizing leakage hazards [51].

Finally, as mentioned above, the objective of this work is to analyze the most relevant works pertaining to the polymer-based cEOR flooding, so that the most suitable and viable polymers may be highlighted to support successful field implementations of polymer-based cEOR in HTHS carbonate reservoirs. It is also worth mentioning that, in the forthcoming decades, the criteria for field application will rely heavily on environmental sustainability and economic factors.

## 2. Challenges Associated with Polymer Flooding in Harsh Conditions

Enhanced oil recovery (EOR) methods have been studied and applied for many years in sandstones (clastic) as opposed to carbonates. Due to the complex conditions of carbonates, including heterogeneity, mixed-to-oil rock wettability as well as harsh conditions of high-temperature and high-salinity [8,52,53], polymer-based cEOR becomes less effective in oil recovery under these harsh conditions. Before we proceed any further, it is important to define harsh conditions to establish a clear understanding for further reading in the paper. The harsh conditioned carbonates may be defined as any reservoir where two or more of the following conditions are encountered [54]:

- Reservoir temperature of over 85 ${}^{{}^{\circ}}$C;
- Formation water salinity above 100,000 ppm (TDS);
- Concentration of divalent cations (hardness) above 1000 ppm;
- Permeability lower than 100 millidarcies (mD);
- Heterogeneity and complex structures of carbonates.

**Figure 5:**
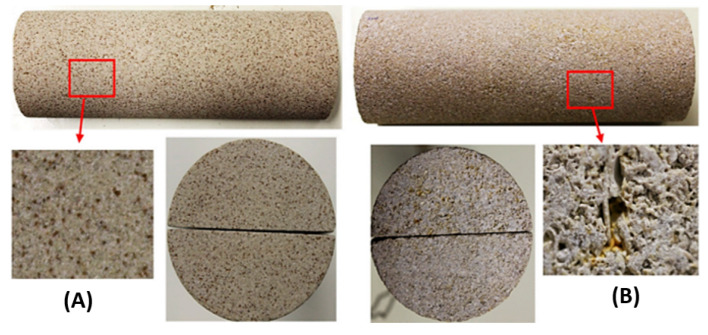
Images of (**A**) sandstone rock sample with relatively homogeneous matrix and (**B**) carbonate rock sample with heterogeneous matrix and vuggy porous media (Reprinted from Telmadarreie and Trivedi, 2018 [55]).

With regards to polymer flooding, it is apparent that high salinity and high concentration of divalent cations (hardness) present the main issue of synthetic polymers, while high temperature and low permeability present the main hurdle for biopolymers. In addition, the complex structures and the heterogeneous vuggy matrix carbonate formation, as shown in Figure 5, make the process of polymer-based cEOR more challenging [55].

Finally, it is important to note that these challenges are usually case dependent and differ from one reservoir to another. This paper focuses on harsh conditioned carbonate reservoirs, which possess high-temperature and high-salinity (HTHS) conditions.

### Instability and Degradation of Polymers under HTHS Conditions

**Figure 6:**
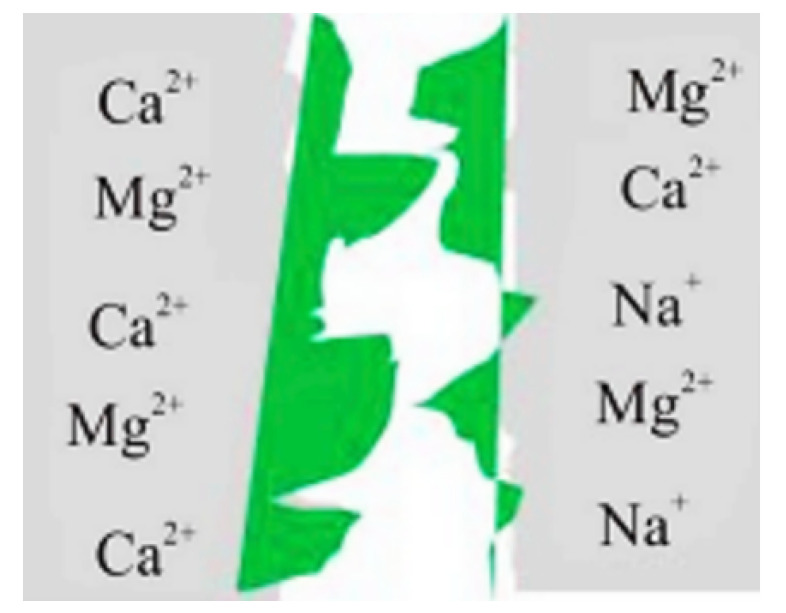
Adsorption of polymer molecules due to divalent ions causing permeability reduction under HTHS conditions (Reproduced from Firozjaii and Saghafi, 2020 [32]).

Polymers can be broadly classified into two types, which are used for oil recovery methods, namely, synthetic partially hydrolyzed polyacrylamide HPAM (and its derivatives) and bio-polymers, such as xanthan [56]. Although polymer-based cEOR is a cost-effective (i.e., economic benefits) method, serious challenges and drawbacks are associated with it. The adsorption of HPAM in carbonate reservoirs is higher than in sandstones, possibly due to the strong attraction forces between the negatively charged carboxylates on the HPAM backbone and the positively charged calcite surface, as illustrated in Figure 6 [32]. This leads to instability (i.e., precipitation) and degradation (i.e., reduced viscosifying power) of polymers under HTHS conditions [33].

Polymer adsorption may cause damage to the reservoir though damaging the formation and the well casing owing to the plugging problem [40]. Additionally, HPAMs (synthetic partially hydrolyzed polyacrylamide), the most extensively used polymers for cEOR applications [57,58], are prone to degrade or hydrolyze under harsh conditions, particularly in a high salinity environment [33].

Furthermore, the adsorption of HPAM in carbonate reservoirs is higher than in sandstones, possibly due to the strong attraction forces between the negatively charged carboxylates on the HPAM backbone and the positively charged calcite surface. This leads to instability (i.e., precipitation) and degradation (i.e., reduced viscosifying power) of polymers under HTHS conditions [33]. The degree of polymer adsorption increases with increasing brine salinity (NaCl concentration) and reservoir temperature. Xu et al. [59] observed that the degree of polymer adsorption increases with increasing brine salinity (e.g., NaCl concentration) [59]. Also, even though the xanthan bio-polymer is capable of tolerating very high salinity conditions, it is afflicted with issues of poor injectivity and bio-degradation [60,61,62,63].

**Figure 7:**
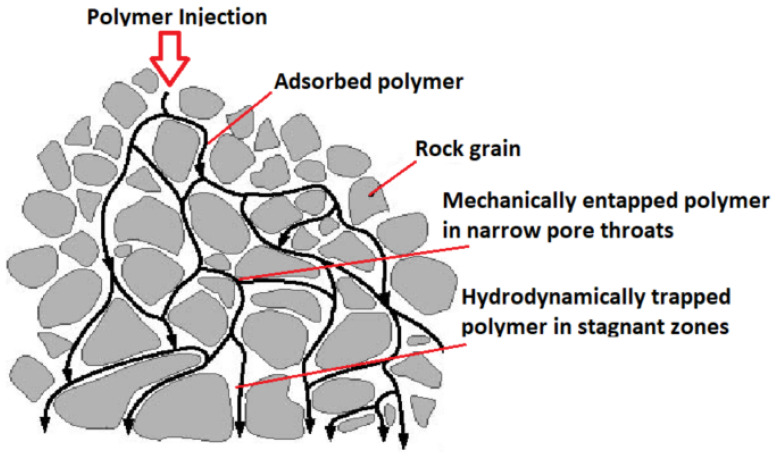
Several polymer retention mechanisms during polymer injection.

Additionally, while operating polymer flooding, the extrication of polymers and the recycling from the production flow is a major challenge, which can substantially increase the project cost [38]. Moreover, when polymer adsorption occurs, it is almost completely irreparable (i.e., it takes a large pore volume of displacing fluid to release the adsorbed polymer); the polymers adsorbed into the pores irreversibly alter and spoil the formation. Consequently, when there is some oil left in the formation after polymer flooding (i.e., polymer flooding generally cannot displace the residual oil out), it becomes more strenuous to recover this oil by other cEOR methods, as the formation permeability is considerably decreased [40]. Therefore, HPAMs have not been implemented in the high salinity (180,000 ppm) and high temperature (120 ${}^{{}^{\circ}}$C) reservoirs of the Middle East [64,65]. At these conditions, the currently available HPAM-based polymers undergo both chemical and thermal degradation [66,67]. Additionally, there is mechanical trapping i.e., when polymer molecules are of greater sizes than pores in the porous media, leading to entrapment of these molecules in small pores. These adsorbed polymers remain trapped on the reservoir rock surface, as shown in Figure 7, which negatively affects the polymer flood efficacy. This is also an undesirable effect environmentally, causing pollution to the reservoir [58,68,69].

In summary, to achieve a functionable mobility ratio in HTHS reservoirs by making up for undesirable temperature and salinity effects, higher concentrations of polymers are required for polymer floods [65,70]. So far, a very small number of studies have examined polymer-based cEOR in carbonate reservoirs under harsh conditions. However, in severe circumstances, such as in the harsh carbonate reservoirs, polymers with greater tolerance to these conditions are essential to ensure a stable recovery in reservoirs for extended periods of time [64,71].

## 3. Mechanisms of Polymer Flooding in Harsh Carbonate Reservoirs

In the preceding works, numerous mechanisms have described the process of increased oil recovery through polymer-based cEOR. The principal recovery mechanism is viscosification of the injected fluid, which is achieved by adding polymers, to supplant hydrocarbon, which results in improved recovery [38]. Unlike water, this highly viscous polymer solution sweeps the oil in the porous medium and improves oil production. The heightened viscosity ($\mu_{w}$) of the brine (aqueous phase), combined with the reduced effective permeability of water ($k_{w}$) through polymer floods is together denoted by the mobility ratio (*M*), which is defined as follows (i.e., given by Lake [9]):

(1) $$M=\frac{\lambda_{W}}{\lambda_{o}}=\frac{W/\mu_{W}}{k_{o}/\mu_{0}},$$

where $\lambda_{w}$ and $\lambda_{o}$ are the brine and oil mobilities, respectively, $k_{w}$ and $k_{o}$ are effective permeabilities of the brine and oil, respectively, and $\mu_{w}$ and $\mu_{o}$ are the brine and the oil viscosities, respectively. The mobility ratio describes the competition to flow between two fluids in a porous medium, in this case, viscous water and oil. Low values of mobility ratio (M<1) indicate piston-like displacement and high sweep efficiency. In the case of high mobility ratio (M>1), the discussion of polymer flooding becomes relevant. Increasing the displacing water viscosity by the addition of polymer results in improving the mobility ratio and ultimately improving the sweep efficiency [9,68,72].

Moreover, the addition of polymer to the injected water can improve the oil recovery efficiency (*RE*), which is given by Lake [9] as follows:

(2) $$RE=E_{D}\times E_{V}=E_{D}\times E_{I}\times E_{A},$$

where $E_{D}$ is the displacement, $E_{V}$ is the volumetric, $E_{I}$ is the vertical, and $E_{A}$ is the areal sweep efficiencies [9]. Polymers improve both $E_{D}$ and $E_{V}$ by viscoelasticity and diverting the water to the unswept regions, respectively. Hence, polymer flooding improves the oil recovery efficiency. The improvement in $E_{V}$ is the primary incentive for polymer flooding. Furthermore, in polymer flooding, the oil production plateau is reached much more rapidly, compared to a regular water flood that requires several pore volumes. The latter promotes better project economics.

In summary, the key mechanism behind enhanced oil recovery through polymers is the viscosification (i.e., thickening) of the polymer solution. The polymer solution is injected to enhance the mobility ratio, resulting in improved sweep efficiency in a porous medium, which displaces the oil. Moreover, polymer flooding reduces the permeability of the formation, making water flooding after polymer injection more effective.

## 4. Polymer Screening Criteria and Requirements for Polymer Flooding

Different screening criteria have been proposed over the years for selecting the most suitable polymer based on laboratory studies and field implementations [11,43,73,74,75,76,77,78,79,80]. The main selection guidelines can be summarized as follows: polymer chemistry, polymer structure, polymer size, rheology, and environmental impact.

The properties of polymers vary greatly. Therefore, it is necessary to select the appropriate polymer for a particular application. It is vital to study the reservoir permeability and oil viscosity when determining the ideal molecular weight of the polymer [38]. The thermal stability of the polymer in high-salinity brine at high temperatures is also important, so the cloud point of the polymer solution must be minded [43]. Polymer retention is another essential property, which includes the possible mechanisms responsible for reducing the mean velocity of polymer molecules during their flow through porous media [43]; it is endorsed mainly to polymer adsorption. It is important to know the rock composition and polymer adsorption level to determine the best degree of hydrolysis (anionicity) because some polymers can be entrapped in a porous medium mechanically or hydrodynamically [38,43]. Polymer selection screening tests are inexpensive and important to derive knowledge of solution rheology, thermal stability, and salinity tolerance. Role of rheology, viscosifying ability, long-term thermal stability, sensitivity for salinity and hardness, shear stability, and static adsorption are most important polymer screening tests [8].

### 4.1. Polymer Chemistry

For both synthetic and biopolymers, composition of the synthetic polymer will depend on the temperature and salinity of the injection/formation water. Once injected into the reservoir, the polymer has to spread out in the porous medium for extended durations (months to years depending on the spacing between injectors and producers), while maintaining its viscosity. The tendency of acrylamide moieties to hydrolyze in a higher temperature restricts the usage of conventional HPAM for field projects at elevated temperature and salinity levels [66,71]. Indeed, acrylamide-based copolymers containing high level of acrylate monomers will precipitate when divalent cations, such as calcium and magnesium, are present.

### 4.2. Polymer Forms

The polymer form (powder, emulsion or concentrated liquid form for biopolymers) is prepared based on the specifications of site location, the logistical provisions and the footprint of the dissolution procedure [81]. For instance, the emulsion polymer was chosen for Captain and Schiehallion fields in the North Sea owing to the perilous weather conditions and the practical constraints of handling polymer [82,83].

### 4.3. Polymer Size

The polymer size is strongly affected by the molecular weight and brine composition. Based on the permeability and heterogeneity of the reservoir, the polymer size can greatly influence the performance of a polymer flood [84]. It is essential for polymer chains to enter into the reservoir with the least impairment of the wellbore region and flow much further from the injector, being least affected by adsorption and entrapment. As mentioned earlier, heightened polymer retention due to mechanical entrapment and lower injectivity in the matrix are expected behaviors when the molecular weight is increased. In case the molecular weight is much greater and exceeds the size of the pore throat, the polymer movement will be difficult. Additionally, mechanical degradation will occur when the flow rate or the impairment of the wellbore area is excessive [85,86].

### 4.4. Rheology

Knowledge of polymer solution rheology at reservoir conditions is fundamental in designing and evaluating polymer-based cEOR [87]. Many polymers can be screened based on rheological measurements. To understand the role of rheology in the evaluation of the polymer flood, one must be familiar with the mobility ratio (M) term. The ultimate objective in any polymer-based cEOR process is to achieve a favorable mobility ratio, which is less than 1. In polymer-based cEOR, favorable (M) is achieved by increasing the viscosity of water. Other polymer-based cEOR techniques also utilize oil viscosity reduction to obtain a favorable mobility ratio. Since the main objective is to maintain a considerably high viscosity, it is important to have knowledge of viscosity variation at different shear rates in the reservoir.

### 4.5. Environmental Impact

The $CO_{2}$ emission and environmental footprint can easily be considered as becoming the principal basis for selecting the polymer and in assessing the effects of various aspects of polymer injection [88,89]. Certain disadvantages of traditional polymers, such as HPAM, include adsorption where polymer retention on the reservoir rock surface occurs, which not only affects polymer flood efficacy, but also from an environmental perspective, pollutes and damages the hydrocarbon reservoir. Additionally, the resourcing and recycling of polymer from the production lines is usually not easy for operations and can be costly. Therefore, ultimately, the dependency of the polymer selection lays on the reservoir characteristics, and the features of polymers, such as, polymer chemistry, structural formations, rheology and the environmental and economic costs.

### 4.6. Requirements of Polymer Flooding Agent

Screening is a crucial phase in polymer selection for implementation of any particular EOR. Polymer screening tests are low cost and deduce ample information about the solution rheology, thermal stability, and salinity tolerance. The shear stability (i.e., steady shear and dynamic rheology measurements), viscoelasticity (i.e., viscosifying ability), salt tolerance ability (i.e., sensitivity for salinity and hardness), long-term thermal stability, biological stability, and static adsorption of polymer are factors (i.e., the most critical screening tests) that affect polymer flooding. Therefore, the selection of polymers to improve the oilfield EOR efficiency should meet the following requirements:

- High water solubility and injection performance;
- A required degree of thermal stability and ability to resist shear degradation;
- Suitability with brine salt and hardness;
- Satisfactory chemical and biological stability;
- Environmentally acceptable and non-polluting to oil reservoir;
- Must be reasonably priced and have logistical ease. Almost all the research is focused on acrylamide-based copolymers, hydrophobic polymers, and thermo-viscosifying polymers.

Updated screening criteria for polymer selection before cEOR are provided in Table 1.

Furthermore, in all harsh condition carbonate reservoirs, high temperature and high salinity have the most significant impact on polymer flooding. Therefore, suitability with brine, and thermal stability for extended period need to be first established through screening tests. The latter tests were conducted by Han et al. [91] to identify potential polymers for polymer-based cEOR under HTHS carbonate reservoirs [91]. Despite the fact that HPAM is the most used polymer in the field due to its good water solubility, viscosifying ability, and rheology, the current studies have shifted to novel polymers to evaluate their tolerance to HTHS carbonates for applicability from HPAM.

In summary, as the cEOR operations are shifting to these challenging harsher reservoirs, consequently, the key criteria for the selection of polymer candidates are based on the water solubility and injection performance, thermal stability and low shear degradation, salinity tolerance, chemical and biological stability, low-cost and logistical ease, and ability to be non-polluting.

## 5. Potential Polymer Types Suitable for HTHS Carbonate Reservoirs

There are three classes of polymers available namely, synthetic polymers, biopolymers, and other types based on the source of their production. As was previously mentioned, the current most extensively used polymer for applications in cEOR is partially hydrolyzed polyacrylamide (HPAM), which is a synthetic polymer, followed by the bio-polymer xanthan. Studies have detailed poor adaptability in harsh reservoir conditions by both polymers. In cases where polymers contain negative charges which interact strongly with divalent cations of the injection brine, both biopolymers and synthetic types tend to precipitate, losing a substantial amount of the viscosifying power. It is therefore necessary to have a neutral repeating unit that does not hydrolyze into a negatively charged unit or to have a negatively charged unit that does not lead to charge bridging with divalent cations, such as (Ca${}^{2+}$) and (Mg${}^{2+}$). This statement was also reiterated in the recent literature by Waver et al. [92], V.B. Lee [93], Pu et al. [94], and Delamaide [95], among several others [8,96]. Accordingly, modifications of conventional polymers were engineered to tolerate specific carbonate reservoirs in harsh conditions (HTHS) for cEOR field applications. In this section, the discourse (review) is based on the critical analysis of the new advances in polymer synthesis and assessments of stability under HTHS for implementation in harsh reservoir conditions, which magnifies the possibility of cost-efficient and non-polluting polymer-based cEOR in large carbonate reservoirs in the nearby future.

### 5.1. Synthetic Polymers

As stated earlier, the most extensively used polymer for cEOR applications, is the synthetic polymer HPAM, which is hydrophilic in nature. The HPAM polymer is utilized in over 90% of all the polymer-based cEOR field projects [78]. HPAM hydrophilicity is the characteristic, which makes it dissolve easily in water to establish hydrogen bonds that create a robust viscosifying influence. Moreover, the electrical repulsion effect in the molecular chains completely expands the molecular chains, which leads to a large hydrodynamic capacity [97].

Additionally, extensive studies have been conducted on HPAM for application in polymer-based cEOR because of its availability, relative low cost, and ease of manufacturing on site [41,98]. Moreover, HPAM has proven to be highly successful in moderate reservoir conditions, such as in the Daqing field in China (formation water TDS of 5000 to 7000 ppm, temperature of 45 ${}^{{}^{\circ}}$C, and permeability of 720 mD). Under these conditions, HPAM proved to also be highly effective [41], and the conditions in the reservoir were beneficial in yielding considerably high incremental recovery [98]. However, when it comes to harsh HTHS conditions, the application of HPAM is restricted, as it shows unstable behavior in the presence of divalent cations. Moreover, when temperatures exceed 60 ${}^{{}^{\circ}}$C, the acrylamide groups within the HPAM polymer hydrolyze to form acrylate clusters. Furthermore, when temperatures exceed 75 ${}^{{}^{\circ}}$C and there is a proximity of divalent cations, the degree of hydrolysis rapidly increases, triggering precipitations [99,100,101,102]. Additionally, it was noticed that the polymers with a greater degree of hydrolysis at the start tend to precipitate much faster, and this precipitation is even more aggravated at higher temperature and salinity/hardness [66,68,72]. Thus, the variations in temperature limited for polymer application depends upon the variations in salinity and pH [9,66,68,72]. Observations from studies also suggest that HPAM solutions undergo acute degradation in the presence of oxygen and free radicals [101].

Nonetheless, because synthetic polymers are much cheaper and easier to make than biopolymers, and since they can be customized and adjusted to suit the reservoir conditions, there have been more comprehensive studies on synthetic polymers than biopolymers [41,98]. Several additional formulations have been developed in recent years, and these are covered in the following sections. Most of these novel polymers are acrylamide-based copolymers, hydrophobic polymers, and thermo-viscosifying polymers. The acrylamide polymers application envelope to harsh HTHS conditions can be enlarged by functionalizing/fabricating synthetic polymers with monomers, resulting in copolymers and terpolymers that are more chemically and thermally robust [32,103,104,105].

Accordingly, novel polymers, such as, co- and terpolymers of acrylamide and hydrophobic polymers, thermo-viscosifying polymers, etc., have been developed to be more temperature and salt resistant, and non-reactive with carbonate surfaces, as discussed below. PAM can be divided into three categories. The first category consists of those copolymers or terpolymers synthesized by incorporating rigid or stiff monomers [106,107]. These monomers are more resistant to chemical degradation, more resistant to cation shielding, and can sterically hinder the polymer chain to keep the hydrodynamic radius at a reasonable value at high salinity. The second category includes those polymers synthesized by adding a hydrophobic monomer (attracted to oil since the procedure takes place in either oil or water) so that intermolecular association can enhance the viscosity [43]. The third category consists of PAM-based thermo-viscosifying polymers with a thermosensitive monomer on the primary hydrophilic chain [97,108,109,110].

Additionally, to improve the viscosity retention of PAM, Shepitka et al. [111], proposed that the addition of intramolecular imide rings can increase the chain stiffening and the viscosity retention. Similarly, sulfating the polyacrylamide could improve the polymer tolerance to an elevated temperature [103]. Furthermore, terpolymer was created by incorporating the hydrophobic monomer N-dodecyl acrylamide, acrylamide, and polar monomer 2-acrylamido-2-methylpropaneulfonic acid using a micellar polymerization in aqueous solution. This synthesized terpolymer was placed in a solution (i.e., concentration of 1500 ppm and viscosity of 18.7 cP) for a 30-day period with temperature 85 ${}^{{}^{\circ}}$C and water salinity of 32,000 mg/L, and the solution viscosity remained unchanged [103].

#### 5.1.1. Hydrolyzed Polyacrylamide (HPAM)

**Figure 8:**
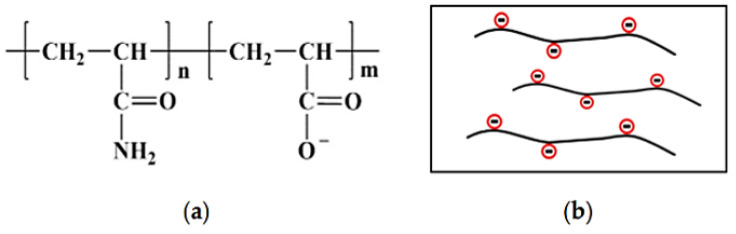
An illustration of the (**a**) chemical structure of HPAM polymer (Reprinted with permission from Liu et al., 2020 [112]) and (**b**) physical structure of HPAM polymer (Reprinted from Shakeel et al., 2020 [38]).

The commercially used hydrophilic PAM used for cEOR is a copolymer of acrylamide (AM) and acrylic acid or variant of salts and is called HPAM. HPAM is highly hydrophilic, forming hydrogen bonds easily, and dissolves in water readily, creating a powerful tackifying (i.e., viscosifying) effect [97]. Additionally, the electrical repulsion amid molecular chains stretches the molecular chains completely to deliver a large hydrodynamic volume [97]. The chemical structure and illustrative physical structures are shown in Figure 8.

When typical concentrations of divalent cations occur in a brine (5–10% of the total salinity), HPAM polymers will hydrolyze and precipitate in high temperature reservoirs i.e., compromising their utility at over 70 ${}^{\circ }$C temperatures [102,113,114]. If a substantial amount of soft water is available, HPAM solutions should be conceptually applicable in reservoirs up to 100 ${}^{\circ }$C, given that the total polymer concentration is viscous enough for sweeping through the reservoir with minimum mixing with the hard formation water [101,115]. This concept necessitates limiting the ion interchange and carbonate dissipation to prevent the hardness from affecting the polymer bank, as it intermingles with minerals in the rock formation [9,116,117]. However, when there is no soft water available, stronger polymers will be required for cEOR applications in reservoirs with temperatures above 70 ${}^{\circ }$C. Additionally, HPAM is usable at temperature of 100 ${}^{\circ }$C, if the reservoir brine has divalent cations less than 200 ppm [118].

Furthermore, Wang et al. [103] discovered that HPAM silica possessed thermal stability for extended durations as opposed to HPAM, when in synthetic brine. Wu et al. [104] researched the application of polymer-based cEOR for extremely high temperature and salinity in an oilfield in Chinese province. They employed a novel SMG (i.e., soft micro gel) as a new polymer, which exhibited excellent tolerance to the HTHS condition. The oil recovery factor improved from 44% to 66%, following the experiments. The rheological behavior of HPAM is most affected by the reservoir temperature, the amount and type of salts contained in the reservoir brine, the impact of shear while being injected, molecular weight of polymer, and interactivities with the surfactant in the case of surfactant–polymer-based cEOR. Sandstones have a high concentration of COO clusters, which initiates a high degree of hydrolysis (DOH) that reduces HPAM adsorption [118]. Contrariwise, in carbonates, due to the positive surface charge, HPAM adsorption is high because of the decrease in DOH, which leads to viscosity loss [107]. The hydrolysis of HPAM begins from 25 ${}^{\circ }$C to 90 ${}^{\circ }$C, inducing viscosity loss of the polymer solution as the temperature contributes substantially to thermo-thinning of the HPAM solution [118]. Hence, by and large, HPAM can be applied in temperatures up to 75 ${}^{\circ }$C only, when exposed to divalent cations [119,120].

#### 5.1.2. Temperature- and Salt-Resistant Cross Linker and Polymer

Temperature- and salt-resistant monomer polymers are a large side group which does not hydrolyze easily, and can restrict the hydrolysis of amide group into the molecular chain of HPAM that forms various co-polymers, and this leads to better temperature and salinity tolerance of the polymer-based cEOR agent [40,121]. In a polymer water solution (HPAM), the flexible chain has the structure of randomly distributed coils under HTHS environments, and the crimp effect on the molecular chain increases the polymer solution viscosity. However, this flexible chain is prone to degradation via mechanical loss, and this limits its application in the field. Nevertheless, the temperature- and salt-resistant type of polymer can be applied in certain reservoir formations under an environment of high-temperature and high-salt conditions [84].

Liu Kun et al. [40] developed a highly viscoelastic flow gel formed by temperature- and salt-resistant cross linker and polymer. This proposed flow gel can effectively block the large pore channels and reduce the permeability of the sweeping phase and, together with injection pressure, increase the low-pressure wells. Consequently, these combined effects attained both deep profile control and displacement effect improvement. Thus, the terpolymer enhanced the displacing fluid viscosity, which diminished the fingering and tonguing occurrences that take place during polymer flooding to enlarge the swept volume and augment polymer-based cEOR efficiency [40].

In another development, Sarsenbekuly, Bauyrzhan et al. [122] investigated a novel functional modified-PAM-based polymer (RH-4), which behaved positively in relation to the viscosity and hydrophobicity when under the effects of salt and temperature. With the help of a scanning electron microscope (SEM), the viscoelasticity of aqueous solutions of the novel RH-4 polymer was observed to alter according to the shear rate when under high-salinity conditions. It was observed that the viscosity decreased at lower salt concentrations and increased at higher concentrations of salt. The SEM showed the condensation of the network structures of RH-4 polymer high salinity, resulting in the obvious viscosity increase, which was the strongest at 80,000 mg/L salinity. Additionally, the increase in temperature caused the viscosity of several concentrations of polymer solutions to decrease slightly. Nonetheless, at 1000 mg/L and 1100 mg/L concentrations of the polymer, the viscosity increased with the rise in temperature [122].

#### 5.1.3. Co-Polymers of Acrylamide

These are polymers containing a co-polymer of acrylamide and 2-acrylamido-2- methylpropane sulfonic acid. The 2-acrylamido-2-methylpropane sulfonic acid (AMPS) monomer with high thermal tolerance can be co-polymerized with AM to produce a hydrophilic anionic polymer that has robust thermal stability. There have been several studies carried out to improve the overall efficacy of polyacrylamide by adding salt-tolerant and temperature-tolerant monomers in greater quantities. Co-polymers of acrylamide-based hydrophilic polymers, which are prepared by using these monomers have better temperature and salinity tolerance in polymer-based cEOR [43].

#### 5.1.4. Comb Type Polymer

Another class of acrylamide (AM) modified polymer is the comb-type polymer, which consists of both hydrophilic and lipophilic groups on the same chain segments. Through an esterification reaction of acrylic acid (AA) and t-octylphenoxypolyoxyehylene (OP) a comb polymer of acrylamide and non-ionic amphiphilic macromer (OPAE) was synthesized [123]. It was observed that the intrinsic viscosity of this macromer in the salt solution was superior in comparison with HPAM, and this was described by different investigations on the use of comb-shaped polymers in cEOR [124,125].

#### 5.1.5. Co-Polymer of Acrylamide and Vinylpyrrolidone

Vinylpyrrolidone (VP) is another important monomer employed to co-polymerize with AM for polymer-based cEOR applications. The homo polymer of VP (PVP) has excellent thermal stability in aqueous and salt solutions 100 but PVP lacks viscosifying power, as almost 10 times higher concentration is required to attain the corresponding HPAM viscosity. The price of PVP is 3 times more than PAM [126] and it is prone to high adsorption on rocks [127], which make PVP inapt for EOR applications.

The proposed mechanism of hydrogen bonding between VP and AM enhances AM robustness, yet, the low polymerization activity makes it challenging to acquire a polymer of high molecular weight [128]. It was observed that the co-polymer of VP and AM with an equal monomer weight ratio in seawater at 120 ${}^{\circ }$C is stable for several months [126]. Additionally, owing to the large size of the VP, incorporating it on the AM chain enhances the shear stability when compared to the homo polymer of the AM group [61].

Kulawardana et al. [129] showed that by replacing some acrylate units in HPAM molecules with functional groups, such as the monomer groups of 2-acrylamido-2-methylpropane sulfonic acid (AMPS), poly vinylpyrrolidones (PVP), acrylamide (AM) and N-vinylpyrrolidones (NVP), HPAM can be modified for application in reservoirs with high salinity and high temperature (HTHS) [129]. Thus, adding a terpolymer of AM, NVP and AMPS resulted in an outstanding thickening capacity in high-salinity condition [130]. Moreover, increased divalent ions tolerance was observed when HPAM was replaced by AMPS [66]. Apart from polymer flooding, these novel polymers could be improved for CO${}_{2}$ foam flooding, which is expected to be an effective strategy in future EOR projects, and therefore, more research is needed.

#### 5.1.6. Terpolymer

The novel PAM terpolymers such as those synthesized by Uhl et al. [131] have ionic surfactant molecules of the acrylamide–sodium acrylate copolymer as side chains. These terpolymers were synthesized by integrating rigid monomers, which are highly resilient against chemical degradation, cation shielding, and can deter the polymer chain sterically, keeping the hydrodynamic radius at a specific value at high-salinity conditions. Considerably greater viscosity was observed in the terpolymer than in HPAM; however, the thermal stability of these polymers has not exhibited any marked improvement from that of HPAM [43,131].

Consequently, these terpolymers are not the best alternatives for high-temperature reservoirs, owing to its high shear thinning properties; however, its injectivity is superior compared to HPAM [132].

#### 5.1.7. Cationic Polymer

In the available literature, there are very few reports on cationic polymer-based cEOR applications. These are not applicable in sandstone reservoirs due to the strong interactivity between the cationic polymer and the negatively charged rock minerals (i.e., silica). Nevertheless, since the greater number of reservoirs with residual oil are carbonate reservoirs, these cationic polymers can be a useful candidate for carbonate reservoirs due to low reactivity with the positively charged rock minerals (i.e., calcite and dolomite) [43]. A cationic terpolymer of AM-VP and methacrylamide propyl trimethyl ammonium chloride (MP) was synthesized by Fernandez [133]. With an increase in the vinylpyrrolidone (VP) content in the terpolymer, the DOH decreased and viscosity preservation was higher, a behavior that was also evident in the copolymers of VP. With a different copolymer of MP and AM having 10% MP content, 94% DOH was achieved when aged for 15 days at 120 ${}^{\circ }$C, and additional aging at 120 ${}^{\circ }$C resulted in molecular weight deficit. It was observed that the AM chain size is key to thermal stability, as the polymers with the shortest AM chains show better stability because of the strong interactions between AM and VP. Another cationic terpolymer of acrylamide was reported by Zou et al. [134] with allyl-β-cyclodextrin, and dimethyl diallyl ammonium chloride [134].

In summary, cationic polymers have not yet been practically implemented, but ought to be considered for lab-scale examinations because of the possibility of low adsorption in carbonate reservoirs and the potential application in HTHS carbonates.

#### 5.1.8. Hydrophobically Modified Associating Polymers

These hydrophobically associating water-soluble polymer are created by introducing hydrophobic groups on the hydrophilic macromolecular chain of polymer, resulting in hydrophobic association within and between molecules. This leads to the formation of a huge three-dimensional network structure, resulting in shear- and salt-resistant polymers [135]. Additionally, because of its unique network structure and physical crosslinks of macromolecular chains, hydrophobically associating polymers can recover some viscosity when the shear force is decreased [136]. Apart from having good temperature and salt resistance, these polymers exhibit considerable thickening behaviors, which could be appropriate for effective operation demands of polymer-based cEOR technology in field applications [137].

Jaing et al. [138] developed a hydrophobic associating polymer-based cEOR agent P(AM/AA/BEM) with exceptional temperature resistance, salt resistance, and high solubility in a fragile alkaline environment. From their experimental studies, an excellent viscosifying behavior was observed with heat resistance up to 90 ${}^{\circ }$C salt tolerance up to 20 g/L, and shear thinning capability. Jaing et al. [138] also observed a strong synergistic effect between (PAM/AA/BEM) and SDBS (sodium dodecyl benzene sulfonate). Upon adding 400 mg/L SDBS in 1 g/L P(AM/AA/BEM), the viscosity of this hybrid system increased by 3.3 times. Better thickening behavior was seen in P(AM/AA/BEM), including superior heat and shearing resistance as well as salt tolerance as opposed to HPAM. Good shear recovery property, viscoelastic property, and tackify effect were also noticed in hydrophobically associating polymer [139,140]. As a result, contemporary research has developed hydrophobically associating polymers with a variety of benefits, including temperature resistance, salt resistance, and shear resistance [135,136,137,138,139,140,141].

In addition, the research on the mechanisms of this polymer is still at a early stage and hence, more studies should work on strengthening the mechanical properties, solubility, wear resistance and creep resistance of hydrophobically associating polymer for prospective practicability in HTHS carbonate reservoirs.

#### 5.1.9. Star Polymer

Star polymer is a type of water-soluble polymer produced through a connection of star cores and many polymer molecules. This polymer has a star-shaped main chain, due to which the rigidity of the polymer molecular chain is effectively enhanced, while the polymer possesses regular molecular structure [56,142]. These attributes prevent the molecular chain of the polymer from curling, thus widening the hydraulic radius of the molecular chain rotation, and significantly improving the temperature resistance, and salt resistance, as well as the viscosifying ability of the polymer. Star polymers are widely used in oil fields as oil flooding, mud assisting, and plugging agents [143]. Nevertheless, star polymers have certain limitations in relation to the acid–alkali stability state, and regarding their solubility and shear resistance capabilities. Thus, further experimental studies are needed to determine the acid–alkali stability, solubility, and shear-resistance ability of star polymer for potential effective cEOR applications [144].

#### 5.1.10. New Smart Thermoviscosifying Polymers (TVPs)

New smart thermoviscosifying polymers (TVPs) were defined by Wang et al. [109], which are designed to maintain the viscosity of the solution phase at high temperature and high salinity reservoirs. Wang et al. suggested that the apparent viscosity and the elastic modulus increased for TVP aqueous solution as the temperature increased, contrary to HPAM solution in a similar condition. The results from the Wang et al. studies demonstrated that TVPs have much potential for utility in polymer-based cEOR in harsh-conditioned carbonate reservoirs [109]. Correspondingly, in another work by Li et al. [145], the salt-induced viscosifying property and mechanism of a TVP solution were investigated. They also evaluated the overall performance of the TVP when used as fracturing fluid under the Jianghan inter-salt shale oil reservoir conditions in China. Li et al. observed that the salt-induced viscosifying characteristic of the TVP solution reduced when temperature and shear rate increased. However, with higher polymer concentration, the salt-induced viscosifying property increased [145]. This was ascribed to the increase in quantity of intermolecular hydrophobic domains as the salt concentration increases, which strengthens the 3D network structure, leading to greater viscosity. Furthermore, the TVP fracturing fluid that was formulated with saturated brine displayed viscosity above 50 cP post shearing for a duration of one hour at temperature of 140 ${}^{\circ }$C. The formulation retained the sand-suspending stability for over one week at temperature of 100 ${}^{\circ }$C. In addition, within 12 h, the fracturing fluid was disintegrated using 0.2 wt%–0.3 wt% potassium persulfate, which left no residue [145]. Accordingly, the results from Wang et al. [109] and Li et al. [145] studies have demonstrated that TVPs can be viable candidates for polymer-based EOR in harsh-conditioned carbonate reservoirs.

### 5.2. Biological Polymers

Enhancing the properties of bio-polymer for stability in HTHS condition differs in several ways from synthetic HPAM-based polymer [43]. In the following sections, some recent viable biopolymers developed for high-temperature and high-salinity tolerance, with apparent suitability for harsh carbonate environment, are discussed.

#### 5.2.1. Xanthan Gum

**Figure 9:**
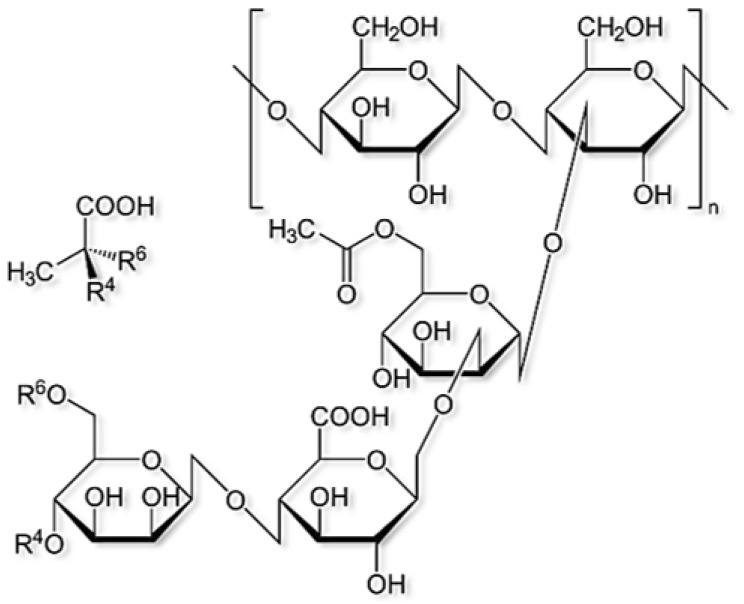
An illustration of chemical structure of xanthan gum (Reprinted from Lwisa, Essa, 2021 [22]).

Xanthan gum also known as xanthan polysaccharide, is a kind of monospore polysaccharide produced by pseudoxanthomonas, which is an important bio-polymer. The chemical structure of xanthan is as shown below in Figure 9 [22].

Nasr et al. [146] investigated the effect of temperature, pH, and salt content on the viscosity of xanthan gum as a polymer-based cEOR agent. It was observed that temperature and salt content did not affect the xanthan gum’s characteristics, and the xanthan gum solution retained at least 80% of the original viscosity [146]. Additionally, it was found that xanthan gum was the most suitable solution for EOR when the temperature increased to 120 ${}^{\circ }$C. Xanthan gum also possesses good suspending, thickening, and emulsifying properties, as well as water solubility and acid–base stability. As a result of its strong qualities, xanthan gum is commonly employed in polymer-based cEOR. However, it still has several drawbacks, such as low shear resistance [147]. Xanthan gum has a more rigid molecular chain than polyacrylamide, making it more resistant to mechanical damage. Nonetheless, it is sensitive to bacteria and is easily to be degraded by bacteria, which might cause the reservoir profile to be blocked. As a result, bactericide and deoxidizer are required for proper cleaning [148]. Consequently, xanthan gum could be a high potential candidate for polymer flooding in harsh carbonate environments, owing to its robust HTHS-tolerant properties.

#### 5.2.2. Scleroglucan

Scleroglucan is a type of non-ionic polysaccharide obtained by the fermentation of the pathogen fungus genus Sclerotium. Due to the rigidness of the scleroglucan structure, the viscosity and shear resistance of the polymer is high in an aqueous solution. Under HTHS conditions, the polymer scleroglucan shows good transport capabilities in both sandstone and carbonate cores. Furthermore, the polymer’s triple helix structure improves the biopolymer’s heat stability [129]. The polymer scleroglucan exhibits decent transport capabilities in both sandstone and carbonate cores at HTHS conditions. Moreover, the triple helix nature of the polymer also improves the thermal stability of this biopolymer [129]. According to published data on thermal stability, scleroglucan is thermally stable for 500 days at 90 ${}^{\circ }$C [113], 60 days at 100 ${}^{\circ }$C [149], and 720 days at 100 ${}^{\circ }$C [150]. Scleroglucan’s main drawback is its low filterability, which has hampered its extensive use [129]. Scleroglucan, however, could be a high-potential choice for polymer-based cEOR in HTHS carbonates if more research is done.

#### 5.2.3. Schizophyllan

The biopolymer schizophyllan has been known to have high-temperature and high-salinity tolerance. One of the studies to demonstrate this was conducted by Quadri et al. [119,120], who investigated the potential of schizophyllan under the HTHS condition using the carbonate core. It was observed that the viscosity of the solution remained stable at a temperature of 135 ${}^{\circ }$C and 220 g/L salinity. Eventually, the study of Quadri et al. [119] concluded that schizophyllan is possibly the best candidate biopolymer for HTHS conditions in both sandstone and carbonate rocks, due to the fact that it is environmentally acceptable and its non-ionic nature causes low adsorption on the exterior of the carbonate rocks [119,120]. However, in certain cases, the productions of this polymer may not be cost effective. Nevertheless, generally, the schizophyllan polymer is produced from the fermentation of palm or other waste products from farms. Therefore, in such cases, the manufacture of schizophyllan is economical and enhances the cost efficiency of polymer-based EOR applications [32].

#### 5.2.4. Welan Gum

Another gum in which can be categorized in the same class, due to its production source is welan gum, which is produced by Alcaligenes microorganisms. When polysaccharides or their derivatives are dissolved in water at low concentrations, they form a viscous solution [151]. Natural gum and modified gum are two types of gum that are classified based on the source of their manufacturing. Welan gum, despite having a smaller molecular weight, has higher viscoelasticity than xanthan gum under similar conditions, according to rheological tests [43,152]. Additionally, the network structure may form in solutions of welan gum, and the concentration is strongly related to the dynamic modulus. Moreover, the adjacent double helices of welan gum are arranged in parallel, which forms a zipper model arrangement not seen in xanthan gum, and this is a stable structure in the HTHS environment. Therefore, in light of this data, welan gum can be a promising candidate for polymer-based enhanced oil recovery, particularly in HTHS carbonate reservoirs [43,152].

#### 5.2.5. Hydroxyethylcellulose (HEC)

Hydroxyethylcellulose (HEC) is an environmentally acceptable non-ionic hydrophilic polysaccharide, which is obtained by the chemical modification of water-insoluble cellulose. The advantage of the HEC polymer is that it does not have problems associated with cellular debris, such as xanthan. Therefore, the HEC polymer does not have any injectivity issues like xanthan, making it possibly useful for polymer-based cEOR in HTHS reservoirs [43].

#### 5.2.6. Starch–Graft-Poly (AM-co-AMPS)

In another polymer synthesis process, biopolymer is grafted and copolymerized with synthetic polymers. For instance, starch–graft-poly/S-g-P (i.e., acrylamide-co-2-acrylamido-2-methylpropane-sulfoacid) (AM-co-AMPS) was synthesized based on both natural and synthetic polymers [153]. The authors reported an improved oil recovery compared to HPAM under identical conditions [43]. Overall, biopolymers have been proposed for high-applicability prospects in HTHS reservoirs; however, some problems, such as the debris of biomaterial at the wall of the wellbore create injection obstructions. These are prone to biological degradation by bacteria and other microorganisms, and the production costs can be high, compared to synthetic polymer, such as HPAM. Biological degradation occurs when the biopolymers and synthetic polymers are in a fluid state during the field injection process, but it has more effect on biopolymers [154]. Additionally, biological degradation could damage formation by causing pore blocking. However, there are several reports on the effect of bio-plugging improving the oil recovery, as it restricts the flow in highly permeable porous regions, such as fractured areas [43].

### 5.3. Other Polymers

Aside from the polymers listed above, new polymer synthesis techniques have developed hybrid polymers with nanoparticles, such as multiwall carbon nanotubes (MWCNTs), that can be tuned to meet specific needs. Furthermore, hot melt adhesive (HMA) polymers are a separate class of polymers than PAM polymers and, as detailed below, can be combined with PAM and HPAM to generate stronger hybrid polymers.

#### 5.3.1. Co/Terpolymer and Polymer/Multiwalled Carbon Nanotubes (MWCNT) Hybrid

In recent years, a new technique of applying nanoparticles for polymer-based EOR surfaced as a possible alternative to enhance the efficiency of oil production. The characteristics of injected fluids such as interfacial tension, viscosity, thermal conductivity, and the fluid-rock interactivities that include adsorption and wettability alterations, can be customized and modified by various nanoparticles [155].

Several researchers have developed different altered variants of acrylamide copolymers (polymers synthesized from two different monomers), and terpolymers (polymers synthesized from three different monomers) were developed through free-radical polymerization and through the introduction of multiwall carbon nanotubes (MWCNTs). The MWCNTs help in producing aqueous polymer dispersions with definite desirable features. Investigations were conducted for the interfacial, rheological behavior, and stability of the dispersions under HTHS conditions with a range of pH values, to identify the most eligible of this set of developed polymers for cEOR implementation. It was concluded that the polyampholytic terpolymer and polyelectrolyte copolymer, which have negative sulfonate clusters exhibited enhanced viscosity and stability when MWCNTs were present in both the alkaline and saline conditions, respectively. Furthermore, as compared to pure polymer dispersions in situations where both the alkaline pH and API brine are present, adding MWCNTs to polymers improved oil recovery efficiency while also reducing pressure decline at elevated temperatures of ${}^{\circ }$C. Hence, it is evident that incorporating MWCNT into polymers could have great practicability for polymer-based EOR applications in future oil field projects [155].

#### 5.3.2. Hot-Melt Adhesive (HMA) Polymers

According to the hydrophobic moieties included, hot-melt adhesive (HMA) polymers are categorized into four groups [156,157,158]. These are as follows:

- The associative polymers with a single associating block;
- Telechelic group;
- Multisticker group;
- Combined HMA polymers group.

The viscosity of the HMA polymer improves over time as the polymer concentration rises [159]. However, with a rise in polymer concentration, the viscosity of HMA polymers might change quickly [159]. When compared to their non-modified counterparts, HMA polymers have better shear resistance. Surfactants can either increase or decrease the viscosity of HMA polymers, depending on the concentration of the added surfactant and the interactions between intermolecular and intramolecular relationships [159].

As a result, various research groups across the world are aiming to extend the uses of HMA polymers to HTHS carbonate reservoirs due to their appealing rheological features. Furthermore, when compared to HMA polymers with a blocky architecture that form hydrophobic associations, the adsorption of HMA polymers is not substantial due to random monomer distribution [160,161]. In addition, adsorption reduces with increasing salt concentration in HMA polymers at first, but above a certain concentration, adsorption rises with increasing salinity [160,161]. Feng et al. [140] prepared HMA-HPAM and HMA-PAM by incorporating octyl bromide as a hydrophobic group and reported the effect of salinity on the rheological properties of these associated polymers, demonstrating that HAPAM polymers exhibited enhanced viscosity in either a monovalent or divalent cation aqueous environment. HAPAM solutions are normally shear-thinning fluids in pure water, but when NaCl is added, these polymers show a shear-thickening reaction [140].

As a result of the favorable rheological qualities exhibited in laboratory assessments, HMA polymers may be used for field applications in HTHS carbonates.

## 6. Experimental Laboratory Studies

Investigatory research to evaluate the long-term stability of several recent polymers in high-salinity and high-temperature were conducted, with promising results. The following studies detail the intriguing features of novel polymers, as well as their capacities, limitations, and overall usefulness in field applications during polymer-based EOR in HTHS carbonate reservoirs. These novel polymers include synthetic polymers, such as acrylamide copolymers with ATBS and NVP monomers [70,128], copolymer of AM and AA functionalized with ATBS and AMPS [130], branched-shape polymers (KYPAM and GLPAM), star-shape polymer (STARPAM), hydrophobically associating polymer (HAP) [162], thermo-viscosifying polymers (TVP) [163,164], NVP terpolymer [129,165], and modified acrylamide co/terpolymers developed from free-radical polymerization with multi-walled carbon nano tubes (MWCNT) hybrids [155]. Recent developments in biopolymers such as scleroglucan [150,166] and schizophyllan are also included [119,120].

### 6.1. Novel Polymers

To deploy polymer-based EOR in HTHS carbonate settings, the polymers must be able to withstand extreme conditions (temperatures above 100 ${}^{{}^{\circ}}$C and salt up to 280 g/L) in order to unlock substantial residual oil reserves [44]. Because many of the newer and larger hydrocarbon reserves are currently located in carbonate formations, numerous experiments have been conducted to assess the stability and resilience of novel polymers, such as ATBS-based, PAM and HPAM modifications, TVPs, HMAs, HAPs, AMPS and NVP-based polymers, co/terpolymers, scleroglucan, salt-induced TVPs, soft micro-gel polymers (SMG), and sulfonated polymers. Laboratory studies of some of the most promising new polymers for polymer-based EOR in HTHS carbonate reservoirs are shown below.

#### 6.1.1. Synthetic Polymers

Appropriate monomers can be produced and then polymerized to produce macromolecules with desired properties. The physical properties of the polymer are determined by the number of monomer units, which can range from a few hundred to thousands. It is worth noting that a synthetic polymerization process cannot be stopped precisely when a certain number of monomers have been absorbed into the polymer structure. Following the polymerization events, a mixture of polymer molecules with varying molecular weights is formed. As a result, a polymer’s average molecular weight, which can range from 10${}^{5}$ to 10${}^{6}$, is referred to. The plasticity and polymer shape are formed depending on the types of monomers incorporated into the polymer [167,168]. The plasticity and polymer shape are determined by the monomer types incorporated into the polymer. For example, polymers with aromatic ring monomers have less flexibility, whereas acyclic monomer polymers have more flexibility. Additionally, some polymers chains are cross-linked by covalent bonds that create larger macromolecules with more rigid structures [167,168].

- AMPS, ATBS- and NVP-Based Polymers

As a result of the polymerization process, several new polymers were created. Gaillard et al. [128] conducted long-term thermal stability studies on acrylamide copolymers, including ATBS and NVP monomers in low-salinity circumstances. Protective additives were utilized in these tests, which were conducted in the presence of 200 ppb of O${}_{2}$. These protective additions are typically radical scavengers or sacrificial agents, as well as other chemical additives that aid in the prevention of heat deterioration. Protective additives were utilized in these tests, which were conducted in the presence of 200 ppb of oxygen (O${}_{2}$). These protective additions are typically radical scavengers or sacrificial agents, as well as other chemical additives that aid in the prevention of heat deterioration. A protective package is obtained when you mix a bunch of these ingredients together [128]. The results showed that Flopaam AN125 SH, which was just functionalized with ATBS, could maintain its viscosity in a NaCl solution for 60 days at 110 ${}^{\circ }$C (20,000 ppm). They also discovered that when SAV505 was functionalized alone with NVP, it retained its viscosity for nearly 60 days at 110 ${}^{\circ }$C in a NaCl solution (50,000 ppm), without the need of a protective packaging in a NaCl solution (50,000 ppm). When the temperature was raised to 120 ${}^{\circ }$C, however, a protective container was required to keep the solution viscosity stable [128].

Furthermore, Kulawardana et al. [129] found that after being aged at 100 ${}^{\circ }$C for 120 days in synthetic saltwater with a protective package, SAV505 could preserve over 80% of its viscosity (TDS of 57,670 ppm and hardness of 2760 ppm). Furthermore, Vermolen et al. [130] found that stable conditions were maintained at 120 ${}^{\circ }$C for more than 180 days in a brine salinity up to 200,000 ppm TDS with a divalent ion concentration up to 18,000 ppm in tests conducted on Superpusher SAV522 functionalized with 20–25% AMPS and 35–50% NVP. In addition, the terpolymer exhibited higher resistance to pH and a reduced adsorption in carbonates [130].

In a separate investigation, Alfazazi et al. [70] found that SAV10 (i.e., functionalized with ATBS and NVP) could maintain 90% of its original viscosity for 100 days at 120 ${}^{\circ }$C in a high salinity brine, with comparable results (167,000 ppm TDS and 46,000 ppm hardness). At low flow rates, they detected a shear thinning behavior in SAV10, but at high flow rates, they noticed a shear thickening behavior [70].

In addition, unlike NVP terpolymers, Gaillard et al. attempted to synthesize NVP polymers with either identical or increased characteristics. Gaillard et al. [71] performed a study on copolymer and terpolymers with both ATBS and NVP adjoined to determine the degree of the applicability of sulfonated polymers. In their work, they examined a variety of polymer compositions with various degrees of ATBS and NVP in a range of brine salinities (4800–98,000 ppm) and temperatures (85–140 ${}^{\circ }$C), placed in contact with divalent cations [71]. The key findings of the study of Gaillard et al. [71] were as follows:

- The stability of hard brines increased as the ATBS concentrations in the polymer backbone chain increased, and a proportional increase in temperature tolerance along with the degree of heat was observed.
- After a year at 105 ${}^{{}^{\circ}}$C temperatures, Superpusher SAV37 (containing more than 35 mol% ATBS and no NVP) maintained 60% of its viscosity in a hard brine (84,500 ppm TDS and 6000 ppm hardness). It could also only maintain 45% of its initial viscosity when held in a less saline but harder brine at the same temperature.
- Investigations into the capabilities of Superpusher SAV10 (which had significantly more than 35 mol% ATBS and no NVP) and Superpusher SAV333 (which was functionalized with ATBS and 30–45% NVP) in a hard brine (TDS of 84,500 ppm and 6000 ppm hardness) at a temperature of 140 ${}^{{}^{\circ}}$C revealed that SAV10 could retain over 60% of its viscosity after a 6-month period, while SAV333 maintained over 90% of its viscosity. Dupuis et al. [169] made similar discoveries while studying these polymers.

Dupuis et al. produced comparable results in their investigations on these polymers [169]. Furthermore, various rheological investigations of ATBS-based polymer and injectivity on three carbonate rock samples revealed that these polymers need to be salinity tolerant [170]. Sandengen et al. [171] published another crucial study that found that ATBS could suppress hydrolysis in AM-based polymers. The duration of stability improved as the ATBS content rose (up to 70 mol%). They also discovered that the hardness/salinity of the solution had no effect on the rate of hydrolysis of the AM-ATBS copolymer; however, the solution viscosity was considerably reduced due to the carboxylate complexation equilibrium (Sandengen et al., 2018). Rodriguez et al. [172] showed that an ATBS-based polymer maintained 70% of its initial viscosity after one year at 130 ${}^{\circ }$C in hard brines with salinity of 230,000 ppm and hardness of 20,800 ppm in a study on the thermal endurance of ATBS-based polymers in harsh carbonate reservoir settings. They connected viscosity loss to chemical and thermal deterioration [172].

It should be emphasized that one of the main disadvantages of these AMPS, ATBS, and NVP-based polymers is their cost, which is almost 3–10 times that of HPAM. Even though NVP, ATBS, and AMPS have better thermal stability and salinity/hardness tolerance than standard HPAM, they are more expensive, according to Gaillard et al. [67]. Furthermore, NVPs reduce the molecular weight of the polymer, requiring very high concentrations to achieve the same polymer’s expected viscosity when used without NVP, thereby increasing the cost [67]. Furthermore, NVPs reduce the molecular weight of the polymer, requiring very high concentrations to achieve the same polymer’s expected viscosity when used without NVP, thereby increasing the cost. As a result, using ATBS alone can help to alleviate this molecular weight constraint [172]. Several studies have indicated that if excessive amounts of ATBS are utilized, it can operate as a substitute for NVP. When particularly harsh conditions are present, however, NVP is essential in the case of SAV333, as previously noted. It is also worth noting that increased sulfonation levels cause more pore-plugging issues [173].

Thus, these sulfonated polymers are sufficiently robust to withstand harsh conditions (temperatures up to 140 ${}^{\circ }$C, salinity around 230,000 ppm, and hardness up to 20,800 ppm) and provide the injected water with good viscosifying strength, assisting in effective sweep action through carbonate reservoirs. Despite the likelihood of increased chemical costs, which can be reduced by employing a larger concentration of ATBS instead of NVP, they are extremely viable for HTHS carbonate condition field application.

II. Hybrid HPAM/PAM Modifications

Zhu et al. integrated hybrid nanoparticles of silica into polyacrylamide, and the solution’s properties were evaluated and compared to HPAM under HTHS reservoir conditions, which had a cumulative divalent salinity of 32,868 mg/L and a temperature of 85 ${}^{\circ }$C [174]. According to their findings, adding silica nanoparticles to a solution increases the elastic modulus and apparent viscosity of the HPAM solution [174]. In another work by Zhu et al. [162], several polymer formulations were investigated for cEOR application in harsh environments. In order to increase temperature and salinity resistance, these formulations were suitably changed to adhere to the linear molecular structure of synthetic polymers. These modified non-linear structure polymers were, namely, the branched shape polymer (KYPAM and GLPAM), star-shape polymer (STARPAM), and hydrophobically associating polymer (HAP). GLPAM had the most potential for use in extreme reservoir settings, since it could maintain about 60% of its viscosity for three months at 95 ${}^{\circ }$C in salinity less than 35,000 ppm. Nonetheless, more testing is needed to assess the potential of these polymers in harsh environments, as all of the tests thus far were conducted at temperatures below 95 ${}^{\circ }$C, with salinities less than 35,000 ppm and hardness less than 2000 ppm for short periods of time [174].

In summary, the hybrid silica nanoparticle incorporated PAM can be possibly effective in HTHS carbonate environments, given that these polymers are comprehensively evaluated for higher HTHS tolerance.

III. Thermo-Viscosifying Polymers (TVPs)

Recent research has focused on a novel class of polymers known as “thermo-viscosifying/stimuli-responsive/thermo-responsive/thermos-sensitive” polymers. These were investigated to see if they could withstand polymer flooding in a harsh reservoir environment. The polymer’s main feature, as the name implies, is its ability to increase viscosity as temperature rises. Its behavior, however, is influenced by the salinity of the water. Leblanc et al. [163] and Kamal and Sultan [164] reported that as low temperature increases (while being injected) to high temperature increases (while penetrating into the reservoir), the polymer solution’s viscosity behavior changes, i.e., from shear thinning to shear thickening, which is evident from an increase in viscosity [163,164]. This behavior is controlled by the lower critical solution temperature (LCST) as a threshold, which is unique to the features of thermo-sensitive monomers. When the temperature crosses this threshold, the polymer molecules rearrange themselves in a hydrophobic physical network, producing increased viscosity. Another characteristic of the polymer is that as the solution salinity increases, LCST descends to lower temperatures, which further heightens the properties of thermo-viscosifying polymers [163,164].

Gaillard et al. [71] compared the thermal stability of this family of polymers to that of other ATBS-containing polymers, finding that 3150 ppm of Flopaam AN 132 VHM (functionalized with solely ATBS) was required to achieve a viscosity of 25 cP at 85 ${}^{\circ }$C in a soft saline solution (TDS 17,000 ppm). In comparison, to achieve the same viscosity under the same conditions, just 1500 ppm of thermo-viscosifying polymer was required. Furthermore, after 120 days of aging at 90 ${}^{\circ }$C, both polymers were relatively stable [71]. As a result of the above deliberation, thermo-viscosifying polymers appear to be an attractive candidate for EOR applications in hard reservoir conditions, particularly if ATBS and NVP can be integrated into the polymer backbone for increased stability in harsh settings [163]. Furthermore, because increased viscosity with temperature requires less polymer to achieve the same viscosity as related polymers, such as sulfonated polymers, thermo-viscosifying polymers have a cost-effective advantage.

IV. Modified Acrylamide Co/Terpolymers and Multiwalled Carbon Nanotubes (MWCNTs)

Chemical enhanced oil recovery (cEOR) experiments on different modified acrylamide co/terpolymers which were developed from free-radical polymerization and further incorporated with multiwalled carbon nanotubes to increase the stability of the polymer under high-temperature and high-salt conditions were conducted by Haruna et al. [155]. Haruna et al. [155] investigated MWCNT (multi-walled carbon nano tubes) hybrids via different corefloods for two of the most promising polymers and their MWCNT dispersal. To compare the effect of MWCNT on incremental oil recovery, a non-tampered co/terpolymer was used as a control. Under both experimental conditions, the polymer containing MWCNTs had a higher recovery factor, demonstrating the composites’ exceptional capacity to change the oil/water mobility ratio [155]. Moreover, compared to other samples, the polymer/MWCNT flooding could be more successful due to better mobility control, which was obtained by improving the rheological properties of the solutions. Furthermore, when MWCNTs were added to a pure-polymer solution, it increased oil recovery at an elevated temperature of 85 ${}^{\circ }$C under alkaline pH and API brine conditions, with a minor pressure drop, demonstrating the novel polymers’ potential capabilities for polymer-based cEOR applications. In alkaline and API brine environments, the polymer/MWCNTs also lowered the interfacial tension and contact angle compared to pure polymers. Additionally, the results of the cEOR laboratory tests revealed that viscous power is a crucial factor in achieving improved oil recovery [155].

V. Core Flooding Using Novel Polymers

Core floods were conducted by Levitt et al. [165] to investigate the optimization of polymer-based EOR for low permeability carbonate cores in HTHS reservoir conditions (permeability of 10–100 mD, temperature of 80–90 ${}^{\circ }$C, and TDS of 200,000 ppm) using a pre-sheared 3000 ppm medium-MW AM-AMPS copolymer with a viscosity of 6 cP. On comparing to an identical but un-sheared low-molecular weight polymer (5500 ppm) with the exact same viscosity, no pore plugging was noticed [165]. Kulawardana et al. [129] also executed a core flood in a 150 mD Estaillade carbonate plug, using SAV301 (a NVP terpolymer) at 83 ${}^{\circ }$C. The polymer showed good filterability and dynamic adsorption (35 g/g-rock) [129]. Furthermore, Bennetzen et al. performed core floods on oil-wet, low-permeability (0.3-28 mD) limestone core plugs under high-salinity (167,730 ppm TDS and 53,200 ppm hardness) and moderate-temperature (55 ${}^{\circ }$C) reservoir conditions utilizing Flopaam 3330S (a synthetic copolymer of AM and AA) [175]. Flopaam 3330S demonstrated increased viscosity, shear thinning behavior up to 100 s${}^{-1}$, high injectivity, shear stability, and no precipitation [175]. Gaillard et al. [67] used two carbonate cores to undertake polymer-based EOR studies in another core flood (71 and 150 mD). The brine had entirely saturated the cores (TDS of 79,900 ppm, hardness of 27,000 ppm). At 105 ${}^{\circ }$C and 120 ${}^{\circ }$C, SAV225 (functionalized with ATBS and 20–30% NVP) and SAV333 (functionalized with ATBS and 30–45% NVP) were utilized, respectively. These polymers were shown to have good overall injectivity, propagation, and adsorption properties. When both polymers were tested for thermal stability at the various temperatures after one year, the results revealed that SAV225 held around 60% of its viscosity at 105 ${}^{\circ }$C while SAV333 retained about 70% of its viscosity at 120 ${}^{\circ }$C [67].

In another core flood, Hashmet et al. [62] used SAV10 to undertake in situ flood front monitoring experiments, followed by a simulation, and the findings were history matched. SAV10 demonstrated the ability to function well in tough settings by lowering the mobility ratio and increasing the recovery factor as needed. To speed up the oil production, they suggested using a large polymer slug size and injecting polymer before water breakthrough [62]. Alfazazi et al. [70] used in situ saturation monitoring and a low salinity pre-flush in a low permeability (average 43.7 mD) carbonate core to perform coreflooding with SAV10. The formation water salinity (TDS of 182,062 ppm and hardness of 46,030 ppm) was saturated in their investigation, and saltwater was injected (TDS of 52,880 ppm and hardness of 27,280 ppm). The coreflooding tests were carried out at a temperature of 120 ${}^{\circ }$C. The increased oil recovery was roughly 7–11%, and the mobility ratio, which was linked to the polymer slug, was reduced from 4.1 to less than 1. Furthermore, the low-salinity pre-flush had little effect on oil recovery; however, it did acclimate the cores for polymer-based cEOR [70].

Finally, core floods for light oils in carbonate cores were undertaken by S. Masalmeh et al. [176]. The application envelope of polymer-based EOR can be enlarged to HTHS carbonate reservoirs in the Middle East, according to test results from several research. The following are the most important findings from these studies [67,130,169,176]:

- In the Middle East, a polyacrylamide-based polymer containing high levels of ATBS (SAV10) was discovered to be useful under reservoir conditions [67,130,169].
- When exposed to H${}_{2}$S (500 ppm) and/or oxygen at 150 ppb, the polymer SAV10 was found to be functional [176].
- The polymer met the injectivity criteria for a wide range of injection rates, from 1 ft/day to 120 ft/day, as well as a wide permeability range [176].
- There was a shear thickening characteristic in the polymer as the flow increased, but no evidence of mechanical degradation were found [176].
- The stability of the effluents’ viscosity was also detected [176].
- The injectivity, local rheology, and adsorption in the carbonate core surface were all significantly impacted by crude oil [67,169].

Overall, the ATBS polymer demonstrated acceptable injectivity and could be tailored for injection to meet the needs of the reservoirs. Furthermore, the needed viscosity for both SIMGAP and SIWAP processes was average, indicating that field testing and piloting would be effective [176]. The HTHS-tolerant polymers were identified as the polymers that preserved the majority of the solution viscosity under hard conditions as a consequence of the positive results of the long-term thermal and salinity-stability tests. In addition, the core flood tests revealed better incremental oil recovery, indicating a positive trend in the direction of field pilots in carbonate reservoirs, which are anticipated to be successful operations.

#### 6.1.2. Biopolymers

Kalpakci et al. [150] investigated the long-term thermal stability of Scleroglucan using 30,000 TDS seawater for two years. They discovered that the polymer preserved all of its viscosity at 100 ${}^{\circ }$C, but that as the temperature was raised to 105 ${}^{\circ }$C, around 10–20% of its initial viscosity was lost. In another investigation on Scleroglucan, Jensen et al. (2018) discovered that at 25 ${}^{\circ }$C, salinity, hardness, and pH (3–10) had no effect on its viscosity. Furthermore, at 95 ${}^{\circ }$C, scleroglucan could maintain its original viscosity for up to a year. Experiments also revealed remarkable injectivity in a limestone core (16.5 mD) at 100 ${}^{\circ }$C, as well as excellent shear stability after 100 cycles through a centrifugal pump [150].

Quadri et al. [119,120] investigated schizophyllan in a similar study. The results were promising in terms of the schizoplyllan’s prospective capability under difficult reservoir conditions. This was due to the fact that it maintained its viscosity for roughly eight months at 120 ${}^{\circ }$C in a 201,600 ppm TDS solution under anaerobic circumstances. Furthermore, the polymer showed shear thinning behavior at higher concentrations [119,120]. The polymer also demonstrated good shear stability and filterability at high flow rates, making it stable under injection circumstances. Quadri et al. [119,120] also used schizophyllan to perform coreflooding on carbonate cores in severe settings. The temperature was 120 ${}^{\circ }$C, and the salinity was 167,400 ppm TDS. The permeability of the core samples ranged from 2.8 to 444 mD. The polymer exhibited rapid mechanical breakdown and adsorption in the reduced permeability cores, according to the researchers. The polymer flood results also revealed incremental oil recovery of 7–10% and little adsorption when the cores were of higher permeability [119,120].

A study on gaur gum was conducted by Musa et al. [42] for its use as a natural polymer in polymer-based EOR applications for sandstone reservoirs and to thoroughly assess its applicability for HTHS reservoir conditions [42]. The experiments in the study evaluated the rheological properties of a natural polymer derived from guar gum for stability at high temperature (up to 210 ${}^{\circ }$F) and high salinity (up to 20% NaCl), with agreeable conclusions. Additionally, in the case of low shear rate, about 100 cP viscosity can be attained at 210 ${}^{\circ }$F for the polymer preparation in deionized water. Moreover, the guar polymer exhibited robust viscosity in the presence of 20% NaCl, where the viscosity is sufficient for temperatures less than 190 ${}^{\circ }$F. Furthermore, the flooding experiment illustrated an increased recovery factor by 16%. However, it was observed that guar gum exhibited shear-thinning behavior and is affected easily by microbial degradation; still, it also had appreciable stability under harsh HTHS (i.e., high temperature and salinity) conditions. Hence, this study demonstrated that the guar solution is suitable as an environmentally acceptable polymer alternative to increase oil recovery in harsh-conditioned reservoirs [42]. It is also worth noting that although this study was conducted in sandstone reservoirs, the possibility of similarly favorable outcomes cannot be ruled out for HTHS carbonates. This is because gaur is a relatively new polymer and further studies assessing its range of applications are expected. In summary, during long-term stability tests, the biopolymers such as schizophyllan [119,120] and guar gum [42] showed acceptable viscosity retention under harsh HTHS conditions, and thus, resulted in improved incremental oil recovery. This advancement in HTHS-tolerant biopolymers points to a good trend in the development of more environmentally sustainable polymer alternatives for severe carbonate reservoirs.

### 6.2. Conventional Polymers Mixed with Surfactants and Alkali Flooding

Certain laboratory studies have shown that combining a polymer with an alkaline or surfactant, or both, can improve oil recovery [54,176,177]. First, the alkaline combines with the acid in the oil, resulting in in situ soap. Surfactant and polymer adsorption are reduced by the soap. The IFT is next reduced by injecting surfactant and producing soap, and finally by adding a water-soluble polymer, which improves water viscosity for piston-like displacement and makes the emulsion stable [178]. The experiments listed below were undertaken to evaluate innovative polymer-based formulations for SP and ASP flooding in HTHS carbonates reservoirs.

#### 6.2.1. Polymer–Surfactant (PS) Flooding

Klimenko et al. [54] conducted a series of lab investigations on polymer–surfactant (PS) formulations that addressed various HTHS conditions in limestone and sandstone reservoirs, with a focus on chemical efficiency in such environments as well as technical elements of ASP flooding. Polymers and surfactants were carefully chosen for their chemical stability and efficiency. Surfactants from the ethoxylated carboxylates and ethoxylated sulfonates family were chosen for their experiments, which were created to achieve optimal salinities in the 80–130 g/L TDS range.

The effect of divalent cations on the physicochemical behavior of the COBR system was also carefully investigated. These surfactant compositions were tested utilizing corefloods, which used SAV10 as a thermally stable polymer and preserved the shift in physicochemical phase behavior with oil saturation. Their findings showed that ethoxylated sulfonates-based formulations were effective on limestones, with a residual oil saturation (S${}_{orc}$) of roughly 9% and surfactant retention of 0.26 mg/g of rock. Furthermore, the formulations based on ethoxylated carboxylates were efficient on quartz sand-pack (Sorc 1–5%). As a result of this research, the development workflow and testing protocols for successful cEOR operations in very harsh conditions, as well as numerous new efficient PS formulations that might be used in HTHS, were supplied [54].

Wang et al. [179] conducted two corefloods in which the injection of the surfactant was preceded by a polymer slug by which the polymer adsorption was assessed, in order to analyze polymer–surfactant adsorption on Middle Eastern carbonate rocks. A sulfonated polyacrylamide (AN-125) with a MW of 12 million Dalton was utilized, which is a copolymer of AM and ATBS (25% sulfonation). The cores were soaked with a hard formation brine (91,000 ppm) (TDS of 229,870 ppm). The polymer solution was prepared using a pre-flush of saltwater (57,612 ppm TDS and 26,372 ppm hardness), and the polymer solution concentration was 2000 ppm. Polymer adsorption in 501 mD and 441 mD cores was found to be 121 g/g and 133 g/g after 5 PVs of injection at 100 ${}^{\circ }$C temperature, respectively [179,180].

As a result, as evidenced by the abovementioned core flooding, the innovative surfactant–polymer compositions were effective and stable in difficult conditions. It is also worth noting that the low-salinity pre-flush can help to prepare the reservoir for the polymer or surfactant–polymer slugs that follow. Furthermore, it is clear from the laboratory core floods that these polymer formulations have very low retention, which is an encouraging development for possible field implementations of the novel formulations in carbonates in the near future.

#### 6.2.2. Polymer–Surfactant–Alkali (ASP) Flooding

Klimenko et al. [54] studied recent lab advancements of alkaline–surfactant–polymer flooding (ASP) formulations for different HTHS conditions in limestone reservoirs, in terms of chemical stability and efficiency under such conditions, as well as technical elements of polymer-based cEOR approaches [54]. Micro-emulsion phase behavior tests for ASP application were performed in a carbonate reservoir at a temperature of 100 ${}^{\circ }$C to generate surfactant formulations with ultra-low IFT. The Indiana limestone corefloods provided remarkable oil recoveries at the tertiary stage, with 85.2, 89.5, and 91.8% for the three core floods conducted, respectively, and insignificant surfactant retention of 0.06, 0.02, and 0.11 mg/g-rock, respectively.

**Figure 10:**
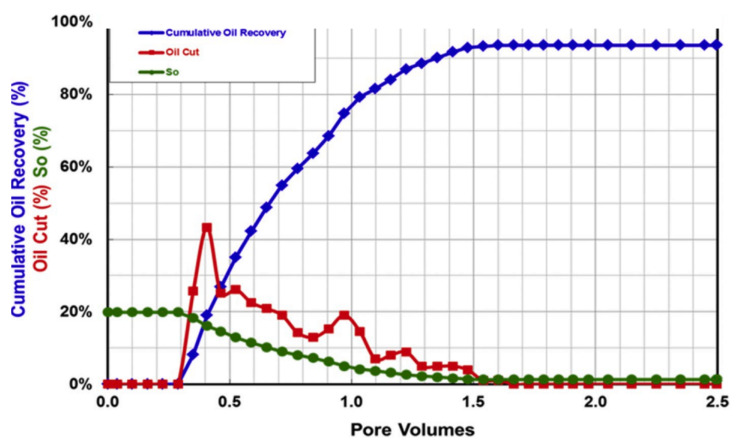
Coreflood results, depicting the oil cut, cumulative oil recovery and the remaining oil saturation (S${}_{o}$) plotted against the injected pore volumes (Reprinted with permission from Abalkhail et al., 2020 [181]).

Abalkhail et al. [181] created an ASP formulation for a high temperature (100 ${}^{\circ }$C) and high salinity (60,000 ppm) large carbonate reservoir with minimal surfactant retention, which is a critical criterion for cheap chemical cost. To find chemical formulations with ultra-low IFT under reservoir circumstances, phase behavior tests were undertaken with anionic surfactants, alkali, co-solvents, brine, and crude oil. In phase behavior studies with surfactant formulations numbered PB-00 to PB-10 [181], NaOH was employed as the alkali in several phase behavior experiments. In addition, the alkali in formulations PB-12 and PB-13 was DIPA-10EO (a novel organic alkali ethoxylated diisopropylamine). In the PB-11 formulation, no alkali was employed. In four coreflood trials, the best two formulations (PB-12 and PB-13) were used. As illustrated in Figure 10, the maximum recovery from core floods in carbonate reservoir cores resulted in 93.6% tertiary oil recovery, 0.012 final oil saturation, and 0.083 mg/g-rock surfactant retention [181].

It may be deduced from the preceding experimental example that novel ASP formulations were effectively devised to resist the HTHS carbonate conditions, and that the issue of chemical retention on the reactive carbonate surface was resolved. These formulations were also beneficial in reducing IFT and altering the wettability of the carbonate cores, resulting in effective oil recovery.

### 6.3. Polymer-Based Hybrid EOR

A hybrid EOR methodology combines two or more separate EOR techniques into a single design [182,183]. Two of the most promising polymer-based hybrid EOR techniques for HTHS carbonates are discussed in this section. The first is low-salinity polymer flooding, which involves flooding the reservoir with low-salinity slugs before applying polymer-based EOR. Under HTHS carbonate circumstances, this is a useful technique to maintain polymer stability while increasing sweep efficiency. The second polymer-based hybrid technique is polymer-enhanced foam (PEF) flooding, in which the polymer not only acts as a thickener, but also speeds up the generation of foam and enhances its mobility in porous media. PEF foam is also more resilient, minimizing viscous fingering and gravity override while improving mobility and sweep efficiency. The two hybrid techniques are discussed in detail in the following sections.

#### 6.3.1. Low Salinity Polymer (LSP) Injection

A hybrid EOR method is defined as any EOR method that combines two or more EOR techniques. A variety of studies are being conducted in the field of hybrid EOR, with the goal of improving the applicability of challenging EOR scenarios.

Karimov et al. [41] conducted an experiment in order to develop a hybrid approach that might boost oil recovery by mixing low-salinity water with polymer flooding to create a synergy effect. Modified seawater and HPAM-based polymer solutions were employed in the studies. Under HTHS circumstances, Flopaam 5115 displayed the least shear-sensitive behavior of the four HPAM-based polymers in terms of concentration, temperature, and salt level. The findings of contact angle measurements and rheological experiments were combined to determine the best conditions for mixing low-salinity water with polymer. At 80 ${}^{\circ }$C, 500 ppm Flopaam 5115 and 0.05× SW, 4 × Ca, and 4× SO${}_{4}$ modified water composition were used to achieve the desired viscosity of 4 cP [41].

In addition, Alfazazi et al. [70] used three core-flooding experiments to investigate the efficacy of polymer-based EOR with low-salinity preconditioning in the HTHS carbonate reservoirs. The tests revealed the barriers to successful polymer-based cEOR in HTHS reservoirs, as well as which approaches could be ideal for overcoming them. To prepare the very saline reservoirs, they suggested injecting slugs of low-salinity brines before applying the polymer-based cEOR. Additionally, the SAV10 polymer was used, which is an HPAM-based polymer obtained in powder form from SNF FLOERGER. This polymer was successful in increasing the mobility ratio, resulting in increased sweep efficiency, and so all of the study’s objectives were met [70].

Furthermore, Al Murayri et al. [184] indicated that the most common deterrents under the HTHS conditions are low-permeability carbonates, which may be addressed by carefully selecting the polymer/ solvent/ co-solvent, pre-shearing, and suitable design of low salinity polymer-based cEOR [184]. They demonstrated that injecting polymer at 113 ${}^{\circ }$C in a reservoir with less than 10 mD permeability was possible in their investigation, which was a long-term stability test [184]. Based on the composition of the injected brine, rheological tests revealed that when the polymer solution had a viscosity of 2–3 cP, a stable flood could be achieved with 2500–4000 ppm polymer. As a result, combining thorough laboratory tests and design with low-salinity water floods proved to be extremely beneficial [184].

In their investigations, Al-Murayri et al. [184] conducted four polymer transport tests with and without a co-solvent and with pre-shearing in order to determine the ideal scenarios for applicability in severe carbonate reservoirs, including economic benefits. The first experiment, in which the polymer was sheared and a cosolvent was added, was the most conservative. A cosolvent was not used with any pre-shearing in the corefloods that followed. Even though the rock formation had limited permeability, the whole polymer core flooding tests (even without cosolvent or pre-shearing) passed the performance standards specified by the researchers with no indication of clogging. Furthermore, polymer floods conducted to assess oil recovery following a low-salinity injection water flood in the tertiary stages revealed significant results. Furthermore, polymer floods conducted to assess oil recovery following a low-salinity injection water flood in the tertiary stages revealed a significant amount of extra oil recovery [184].

Additionally, Lee et al. [185] performed experiments on carbonate core-samples, using low salinity with ionic adjustments followed by polymer flooding. The residual oil saturation (S${}_{or}$) value was considerably decreased by using low-salinity polymer (LSP) flooding compared to conventional high salinity water flooding and engineered water flooding. All designed injection water (IW) solutions (i.e., IW-1: pH 7, 1000 ppm SO${}_{4}^{2-}$, IW-2: pH 7, 4000 ppm SO${}_{4}^{2-}$, IW-3: pH 4, 4000 ppm SO${}_{4}^{2-}$, IW-4: pH 7, 100 ppm Ca${}^{2+}$, IW-5: pH 7, 1000 ppm Ca${}^{2+}$, and IW-6: pH 4, 1000 ppm Ca${}^{2+}$) reduced the S${}_{or}$ value; however, the neutral low salinity water containing only SO${}_{4}^{2-}$ ions gave the lowest Sor value [185].

Subsequently, Yang et al. [186] published works detailing the combined low salinity (LS) and polymer flooding technique in order to target the great heavy-oil reserves on the Alaska North Slope (ANS). A series of core flooding experiments at varying conditions were conducted to show the synergy of LSW and polymer flooding. It was observed from the results that high salinity polymer (HSP) (i.e., salinity 27,500 ppm) necessitates almost two-thirds more polymer than the low-salinity polymer (LSP) (i.e., salinity 2500 ppm) to reach the required viscosity at normal conditions (71 ${}^{\circ }$F). There was additional oil recovery using LSW flooding after extensive high salinity (HS) flooding of approximately 3 to 9% of original oil in place (OOIP). Additionally, LS flooding conducted in secondary mode recovered higher oil volume than LSW flooding in the tertiary mode. It was also possible to delay the water breakthrough in LS flooding in comparison to the HS flooding. Additionally, quite remarkably, even after persistent LS and HSP flooding, the incremental oil recovery of roughly 8% of OOIP was still attained by the LSP flooding that had the same viscosity as the HSP [186]. Furthermore, the increase in pH of the effluent during LS/LSP flooding was considerably higher than that during HS/HSP flooding, which pointed toward the presence of the low-salinity effect. It was deduced that the residual-oil-saturation (S${}_{or}$) decrease brought about by the LS effect in the un-swept area from the LS flooding (i.e., mainly smaller pores) influenced the oil recovery increase. It was also noted that conducting LSP flooding straight after water-flooding retrieved more incremental oil (i.e., about 10% OOIP) than conducting HSP flooding in the same pattern. Therefore, besides the better sweep efficiency by the polymer, it was also noted that the low-salinity-created residual-oil saturation (S${}_{or}$) was causative of the oil recovery increase by the LSP flooding [186].

As a result of these compelling investigations, it can be concluded that low salinity with ionic modifications (smart-water or engineered water) prior to polymer flooding in carbonate reservoirs can be an effective method to reduce the residual oil saturation (S${}_{or}$). Low salinity polymer (LSP) flooding can result in up to 6–10% OOIP (i.e., original oil in place) recovery when followed by PF, showing that LSW alterations of the fluid–rock interactions induce oil volume displacement that is easily conveyed forward by the polymer fluid injection. The synergistic effect of the hybrid LSP flooding process is most pronounced in secondary mode with higher oil recovery volume than recoveries from LSP flooding in the tertiary mode and also reduces water breakthrough.

#### 6.3.2. Polymer Enhanced CO${}_{2}$ Foam Flooding

**Figure 11:**
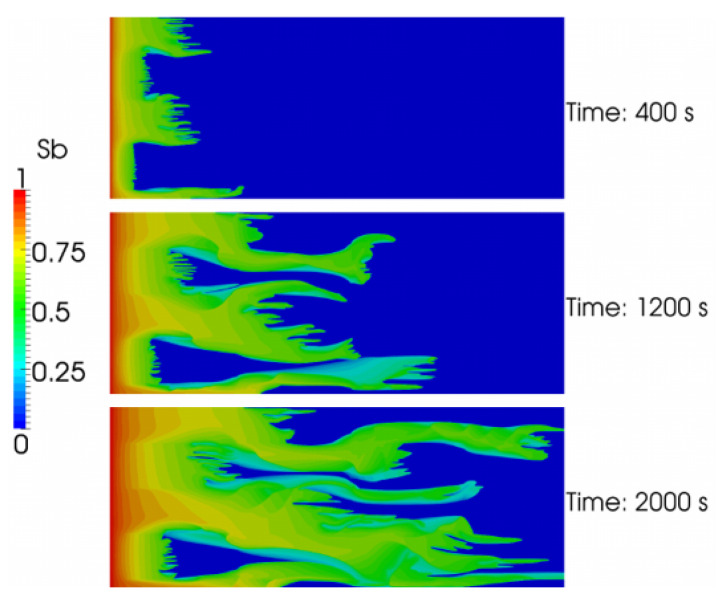
An illustration of viscous fingering in heavy oil reservoir, here, Sb denotes water saturation (Horgue, et al., 2015). (Reprinted with permission from Horgue, et al., 2015 [189]).

Chemical-based enhanced oil recovery (cEOR) methods are the most extensively used non-thermal EOR technologies for heavy oil reservoirs after water flooding. cEOR techniques, on the other hand, are prone to low injectivity and low sweep efficiency, particularly in heterogeneous reservoirs with heavy oil. In these instances, foam and polymer-enhanced foam (PEF) can significantly improve the sweep efficiency over the individual gas and chemical injection methods. Because the foam has a far higher effective viscosity than the gas, it helps prevent viscous fingering and gravity override, which are common during gas injection, as shown in Figure 11 [187]. However, in the $CO_{2}$ foam flooding, the main challenge is the instability or breaking of the bubbles when subjected to harsh reservoir conditions. To overcome this issue, a number of studies were performed; by introducing a polymer into a surfactant solution, the polymer-enhanced foam (PEF) technique could stabilize foams due to dramatically increasing in apparent viscosity. As a result, several investigations created polymers that dissolve in dense CO${}_{2}$ and raise its viscosity sufficiently to operate as direct thickeners for fluid mobility control. On both microscopic and macroscopic sizes, these water-free additions can significantly enhance displacement efficiency [188].

Furthermore, natural fractures characterize carbonate reservoirs, resulting in low oil recovery efficiency. The injected fluids move directly from the injection wells to the production wells through the fractures. This problem is worsened in these reservoirs after CO${}_{2}$ injection, resulting in reduced sweep efficiency due to the displacing agent’s high mobility. Thus, in a study by Chakravarthy et al. [190], the CO${}_{2}$ sweep efficiency was improved in fractured rocks by controlling CO${}_{2}$ mobility. Water viscosified with a polymer and injected directly into the crack was employed as a mobility control to direct the flow of CO${}_{2}$ into the matrix, resulting in a breakthrough delay. The breakthrough was much reduced; however, there was a problem with considerable water “leak off” into the matrix. To combat this, a cross-linked gel was injected into the fractures for conformance control, reducing “leak off” and allowing the CO${}_{2}$ flow to proceed more efficiently into the matrix, improving the displacement [190].

Moreover, water alternating gas (WAG) injection is a prevalent technique for CO${}_{2}$ flooding that limits mobility and improves the volumetric sweep efficiency, with higher recovery ranging from 6 to 20%. Even though the WAG injection recovery is strong, the CO${}_{2}$ flooding sweep efficiency is insufficient to produce higher oil recovery. As a result, Li and Schechter suggested a novel combination technology called polymer alternating gas (PAG) with the goal of improving the volumetric sweep efficiency of the (WAG) process [191]. The polymers are combined with water in the WAG procedure in this unique method to increase the mobility ratio. As a result, the polymer flooding and immiscible/miscible CO${}_{2}$ injection in the PAG process are combined. The study’s findings demonstrate that PAG significantly boosted immiscible/miscible floods recovery in both homogeneous and heterogeneous formations [191]. Other research have explored CO${}_{2}$ foam flooding with mobility control for boosting the reservoir sweep efficiency, with foam potentially improving the reservoir sweep efficiency more than gas injection EOR [192,193]. Apart from improving the sweep efficiency in gas flooding, foam or polymer-enhanced foam (PEF) was also employed in cEOR processes for mobility control, where the created foam functions as a substitute for polymer mobility control during the micellar flooding process [9]. Upon comparison to the performance of chemical flooding, Zhang et al. [194] reported on laboratory and field applications of foam in the Daqing oilfield in China. The foam was successfully used in a heterogeneous porous media [194]. Even though the primary principles of foam creation are widely recognized, the particular circumstances in which strong foams are formed in the reservoir remain unknown. Foam production in homogenous porous media typically requires consistent gas and liquid co-injection with a low-pressure gradient [195,196,197,198]. Surfactant alternating gas (SAG) flooding has been proposed as a way to improve foam formation by alternating rounds of liquid and gas (Rossen, 1990). Due to the reduced contact between water and gas in surface areas [199,200], SAG injection is preferable to the co-injection of gas and surfactant. It improves injectivity and likely promotes foam production close to the near-wellbore section [196].

Despite the fact that many prior research studies have focused on light crude oils, Telmadarreie and Trivedi [55] tested the static and dynamic performances of foam and polymer-enhanced foam (PEF) when heavy oil was present. The surfactants used for forming were Surfonic N85 (Huntsman Corporation, Conroe, TX, USA), a nonylphenol-ethoxylated nonionic surfactant with the chemical formula C${}_{15}$H${}_{23}$ (OCH${}_{2}$CH${}_{2}$)${}_{n}$ OH (Mw = 594 g/mol), two anionic surfactants i.e., sodium dodecylbenzenesulfonate (DDBS; C${}_{12}$H${}_{25}$C${}_{6}$H${}_{4}$SO${}_{3}$Na, Mw = 348.5 g/mol)) and C14–C16 alpha olefin sulfonate (AOS; R-SO${}_{3}$ Na${}^{+}$, Mw = 348.5 g/mol) were used for static foam analysis, and cationic surfactant: cetyltrimethylammonium bromide (CTAB) (Sigma-Aldrich, St. Louis, MO, USA, 99% purity) with chemical formula (C${}_{19}$H${}_{42}$BrN, Mw = 364.5 g/mol) was also utilized as a cationic foaming agent. The polymer employed for viscosity enhancement of the liquid phase was an anionic polyacrylamide polymer (supplied by SNF SAS, Andrézieux-Bouthéon, France). The hydrolysis degree of FLOPAAM 3330S was 25–30%, and the average molecular weight is 8106. Furthermore, in both static and dynamic trials, heavy crude oil (taken from Canadian oilfields) with a dead oil viscosity of 1320 cP (at 22 ${}^{\circ }$C) and a dead oil density of 933 kg/m${}^{3}$ was used. For the static investigation of the foam–oil system, a mineral oil with a viscosity of 27 cP and a density of 850 kg/m${}^{3}$ (22 ${}^{\circ }$C) was also used [55]. The findings of static stability tests revealed that anionic surfactants have a greater power to form more stable foams in the presence of heavy oil. In addition to improving heavy oil recovery, the additional polymer sped up the formation of foam and its transmission across porous media with heavy oil saturation. Optical evaluations revealed that PEF had a higher forward displacement and sweep efficiency than conventional foam flooding. For PEF injection, there was also evidence of reduced channeling or foam rupture [55].

Xu et al. [59] investigated the performance of PEF utilizing AOS (alpha olefin sulfonate) as the foaming agent, which was optimized by a polymer, either HPAM or AVS (anthryl-vinyl-styrene). This polymer was shown to thicken the surfactant solution without affecting its foamability due to the surface-active clusters on the molecular chain. Furthermore, by incorporating the phenyl, sulfo, and other functional groups into the polymer molecule, AVS became more resistant to salt and temperature. From the results, it could be observed that [59]:

- AVS solution demonstrated higher viscosities than HPAM solution under harsh HTHS (high salinity and temperature) conditions.
- Surface tensions between AVS solution and CO${}_{2}$ were lowered, which was attributed to the addition of the surface activity group.
- The foaming factors of AOS/AVS were remarkable in brine salinities ranging from 1000 to 50,000 ppm and temperatures ranging from 25 ${}^{{}^{\circ}}$C to 60 ${}^{{}^{\circ}}$C.
- The CO${}_{2}$ foam blockage that was enhanced owing to AVS was promising and as required at a pressure of 2000 psi.
- When under identical conditions, the oil recoveries of the tertiary phase yielded by AVS/AOS were 5% to 7% higher than those of HPAM/AOS during the core flooding.

As a result, this unique polymer is considered to have a high potential for use in polymer-enhanced CO${}_{2}$ foam flooding in a variety of reservoir conditions [59].

Additionally, Aliakbar et al. [21] carried out a series of experiments to look into the effects of reservoir conditions on CO${}_{2}$ capture efficiency. The injection of flue gas or a CO${}_{2}$–N${}_{2}$ combination into gas hydrate reservoirs is a viable alternative for CO${}_{2}$ geological storage, i.e., CO${}_{2}$ sequestration using carbon capture, utilization, and storage (CCUS) technology. This method can significantly lower CO${}_{2}$ levels in the atmosphere and hence the greenhouse impact [201]. Aliakbar et al. attained the results, where over 60% of the CO${}_{2}$ contained in the flue gas could be captured and stored as CO${}_{2}$ or CO${}_{2}$-mixed hydrates, as methane-rich gas was released. Temperature, pressure, and hydrate saturation were found to have a significant impact on the CO${}_{2}$ capture efficiency in the reservoir. Controlling the injection pressure was shown to be critical for increasing the CO${}_{2}$ capture efficiency when flue gas or CO${}_{2}$–N${}_{2}$ mixed injections were used [21].

In summary, the problem of a low viscosity CO${}_{2}$ displacing fluid causing early breakthrough and lower areal sweep efficiency can be solved by using polymers that dissolve in dense CO${}_{2}$ fluid as direct thickeners. This raises the viscosity of the CO${}_{2}$ fluid enough to improve the injected fluid’s mobility control. Thickened polymer water injected directly into fractures in fractured formations such as carbonates can reroute CO${}_{2}$ flow into the matrix, preventing breakthrough. Furthermore, CO${}_{2}$ foam flooding mixed with mobility control is a hybrid strategy for boosting the sweep efficiency beyond the levels of gas input [192,193]. Additionally, polymer-enhanced foam (PEF) affects mobility control as foam substitutes for the polymer during this micellar flooding hybrid EOR process. Because the effective viscosity of foam is substantially greater than gas viscosity, PEF can alleviate the problem of crude oil as an anti-foam agent, especially in heterogeneous reservoirs with heavy oil. Finally, CO${}_{2}$ sequestration as a means of reducing carbon emissions can be achieved in part by utilizing hydrate reservoirs as geological CO${}_{2}$ storage. Up to 60% of the CO${}_{2}$ in flue gas might be collected and stored as CO${}_{2}$ or CO${}_{2}$-mixed hydrates using CCUS (i.e., carbon capture, utilization and storage) technology.

## 7. Numerical Studies and Field Applications

Because there are few conventional polymers that can withstand high salinity, particularly at high temperatures, various novel polymers have been developed and synthesized that are more resilient under HTHS carbonate reservoir conditions. Numerical simulations are sophisticated programs that facilitate the analysis and estimation of field-scale cEOR models that predict the efficacy of these approaches by evaluating rheology, sweep efficiency, oil recovery, stability, and degree of retention, among other factors. The details of certain advanced modeling programs that consider key parameters to estimate the final recovery from cEOR in HTHS carbonate reservoirs using novel polymer formulations have been presented. This section also identifies important polymer-based EOR field application projects around the world, as well as upcoming prominent initiatives in harsh carbonate reservoirs.

### 7.1. Novel Synthetic Polymers

In recent decade, several novel synthetic polymers, such as modified HPAM-based polymers have been extensively studied for cEOR field pilot projects globally and in varying reservoir conditions [44,78,104,169,176,202,203,204,205,206,207,207]. Nevertheless, to the best of our knowledge, there have only been a few polymer flooding projects undertaken in carbonate reservoirs [175,208,209]. Polymer flooding field applications from 24 countries are listed in the following Table 2. Only around a seventh (1/7th) of them, or 104, have been carried out in carbonates, and only eight have been carried out in offshore oilfields [56].

#### 7.1.1. Field Applications

The first polymer flooding field use occurred in the Northeast Hallsville Crane in the United States, despite a lack of specific information [210]. The temperature in the reservoir was recorded to be 109 ${}^{\circ }$C, with an oil viscosity of 0.09 cP. To the best of our knowledge, there are no reports on the spacing of wells or the compositions of both formation and injection waters available. Polymer-based cEOR was used in the secondary mode, according to reports, and over 900,000 barrels of additional oil were recovered [78]. The Eliasville Caddo Unit, also in the United States, was the second known field application. The unit temperature was 46 ${}^{\circ }$C, the oil viscosity was 3 cP, the formation water salinity was 165,000 ppm, the well spacing was 350 m, and the fresh injection water was only 1200 ppm (400 ppm hardness). In addition, polymer-based cEOR was used in the tertiary mode, resulting in an increase in oil production of over 3,600,000 barrels [78]. It is important to note that the well spacing in this example was quite small, implying that the polymer may not have been stable for a long time, which is unusual in offshore reservoirs and massive carbonate fields.

Several field applications have been completed at this time, and the following are some of the most important recent field applications:

- Wu et al. [104] reported the successful applicability of polymer-based cEOR in extreme temperature and salinity in one of the oilfields in China. They employed a SMG (soft micro gel) in this study, which was a new polymer known to be HTHS condition tolerant. The results obtained had an increased oil recovery factor from 44% to 66% [104].
- A polymer-based cEOR was undertaken in 1996 in Daqing oilfield in China. The incremental oil recovery factor from this field was observed to be 12% more than water flooding [203].
- Since, field application of HMA polymers can be challenging, therefore, based on the available literature, the reported applications of HMA polymers in the field are limited. However, extensive lab-scale investigations are underway to extend the findings to field applications. One pilot-scale test was conducted in Bohai Bay oil field in China using an HMA polymer, where 25,000 m${}^{3}$ of incremental oil was recovered and the water cut decreased from 95 to 54% [202].
- Tiwari et al. [211] successfully conducted a study of polymer-based cEOR at Sanand oil field in the India. HPAM was selected as the water-soluble polymer, where the reservoir had 20 cP oil with temperature of 85 ${}^{{}^{\circ}}$C [211].
- Two polymers 340 and 454 PAM were studied to ascertain their applicability in the Owasco Unit oil field in the United State (US). In this case, the reservoir lithology was sandstone with a temperature of 77 ${}^{{}^{\circ}}$C [205]. The upshots from these projects was that emulsion-based synthetic polymer projects encountered greater issues of injectivity in contrast to the projects where the powder-type synthetic polymers were used. Furthermore, the rate of success for polymer-based projects that employed secondary mode injection was higher than those projects that implemented polymer injection in tertiary mode [78].
- Two polymer-based cEOR field pilots is commenced by ADNOC in two reservoirs which are presently undergoing water-flooding. The targeted reservoirs cover multiple fields in Abu Dhabi [176,212]. The target reservoirs have high temperatures ranging from 100 ${}^{{}^{\circ}}$C to 130 ${}^{{}^{\circ}}$C, high-salinity formation brine of around 200,000 ppm, and high concentrations of divalent ions (Ca${}^{2+}$ and Mg${}^{2+}$) of around 18,000 ppm, with a carbonate reservoir formation. Likewise, one of the targeted reservoirs presents a significant challenge for optimal recovery, as it has a high permeability contrast between the upper and lower zones. This is also a layered reservoir (i.e., the strata), which are a few feet in thickness, which can be correlated field wide, frequently beyond several kilometers [212]. Additionally, the cumulative permeability increases as the top of the reservoir approaches. This reservoir may be characterized at an elevated level as being made up of two main bodies, i.e., an upper zone (high permeable layers inter-bedded between low permeable layers) and a lower zone of low permeability layers of relatively uniform permeability distribution. The permeabilities in the upper part ranges from 10 to many 100 mD, and observations of underwater flooding exhibited good displacement of oil by water. The permeabilities in the lower part are on average much lower than in the upper part, varying between 1 and 10 mD. Thus, overall recovery from these low permeability zones is quite minimal, due to the fact that traditional injectants (i.e., water and gas) will propagate rapidly to the higher permeability regions of the upper reservoir, which overlies the lower reservoir, and a full pressure contact is experienced with it. Additionally, the recent study CO${}_{2}$ on Abu Dhabi reservoirs as mentioned above demonstrate that due to the high heterogeneity of the formation, where water flooding is the more feasible option than both continuous CO${}_{2}$ and CO${}_{2}$ WAG injection techniques [176,212]. Moreover, the specific properties of the Abu Dhabi reservoirs include light crude oil with API ranging from 35 to 40, which eliminates the EOR options that are thermal based. Thus, chemical cEOR alternatives are identified to have much greater potential in retrieving either remaining oil saturation or the bypassed oil in tight pores or low permeability reservoirs. Hence, for these reservoirs at Abu Dhabi, the deduced scheme is a combination of gas cEOR with mobility control options (such as polymer flooding). Even though cEOR methods may be highly challenging in the HTHS carbonate reservoirs in Abu Dhabi, the recently developed polymers have shown high tolerance to harsh carbonate reservoir conditions [67,130,169,172]. Therefore, ADNOC selected polymer-based cEOR as the foremost EOR option (ie.e, new field development plan), which comprises polymer injection, simultaneous injection of miscible gas and polymer (SIMGAP), simultaneous injection of water and polymer (SIWAP), and low-salinity polymer approaches [176,212]. For this field application plan, two polymer-based cEOR concepts were developed for these reservoirs [176,212]:
  1. Simultaneous injection of miscible gas and polymer (SIMGAP): The simultaneous injection of viscosified water (polymer solution) is for the upper highly permeable zone, and miscible gas (e.g., H${}_{2}$S/CO${}_{2}$) is for the low permeable lower zone. This creates a resultant lateral pressure gradient as a result, which is maintained in the upper zone, retaining gas confinement in the lower zone.
  2. Simultaneous injection of water and polymer (SIWAP): The simultaneous injection of viscosified water (polymer solution) will be in the upper zone, and normal water will be for the lower zone. To create a variation of this concept, only viscosified water is injected in the reservoir which also culminates in an identical recovery factor as SIWAP, but consumes a larger volume of polymer for injection.
- Although these novel processes have not yet been implemented in the field, the industry has decades of experience in water flooding, polymer-based cEOR, and miscible gas injection processes to rely on [176,212]. Therefore, these methods (SIMGAP and SIWAP) are a combination of two processes into a single practicable recovery mechanism [176,212]. Furthermore, the simulation results obtained show positive indications with both processes. Thus, these newly developed processes have the capacity to attain elevated recovery factors and evidently improve the sweep efficiency in reservoirs with highly heterogeneity. Consequently, the principal issue concerning these polymers-based EOR processes will be to derive a polymer which maintains its stability at HTHS (i.e., high temperature and high salinity) and has a desirable injectivity in carbonates [176,212].
- The data from the cited experiments [176,212] provided in this paper illustrate that an ATBS polymer was found to be a promising candidate due to the injectivity behavior, which can be adjusted for injection in accordance to the target reservoirs. Additionally, to be workable, both SIMGAP and SIWAP processes require a moderate rise in viscosity. Therefore, the results were found to be very encouraging and suggest successful progression toward possible field testing and piloting. Additionally, to limit the risk of these pilots, a polymer injectivity test was scheduled to commence in 2019. It was expected to begin once a desirable injectivity of the polymer was exhibited in the field; ADNOC will proceed with the initialization of the two polymer-based cEOR pilots to demonstrate the applicability of the two new polymer-based cEOR concepts. Thus, through the tests based on these novel concepts, it is anticipated that a sizable volume of ADNOC reservoirs, both onshore and offshore will become accessible for production [176,212].
- In consistency with their plans, the very first of these injectivity pilot tests for carbonate with bypassed oil within the heterogeneous layers in Abu Dhabi reservoirs were completed successfully. A polymer-based cEOR technique was determined for retrieving the residual and remaining oil. With 10 years in its development, a polymer with high amounts of 2-acrylamido-tertiary-butyl sulfonic acid (ATBS) was used [169,176]. Through screening methods of thermal stability, bulk and in situ rheology, adsorption and injectivity for the harsh conditions of ADNOC reservoirs, their polymer was deemed suitable.
  Employing a de-risking strategy involved polymer injectivity test (PIT) followed by a multi-well pilot that was conducted before the final field implementation of polymer-based cEOR reservoir for several ADNOC reservoirs. The PIT took four months to be completed in February 2019 which was at a 250${}^{{}^{\circ}}$ temperature and salinity >200,000 ppm conditions, with low H${}_{2}$S content [206,213]. A total of 150,000 barrels of viscous solution was expended for injection into the reservoir. The PIT successfully attained the projected crucial performance indicators concerning, the quality of polymer solution, viscosity, concentration, injection rate and skid running time. The latter was confirmed via a dedicated surveillance and injection monitoring throughout the whole period. A new polymer with high (ATBS) content was identified, based on extensive laboratory studies, and an initial polymer injectivity test (PIT) that was conducted. Thus, this positive polymer injectivity test verified the new polymer’s efficacy for field application at HTHS carbonate reservoirs [206].
- Subsequently, ADNOC expanded on their designed polymer-based cEOR application to harsher field environment, which had a higher H${}_{2}$S content. This second PIT was conceived for laying the groundwork for a multi-well pilot polymer flooding project, that dissipated the H${}_{2}$S concentrations (20–40 ppm) to determine the injectivity at prototypical field conditions and in situ polymer functioning in HTHS reservoirs of Abu Dhabi. The PIT was initiated in February 2021 and completed in July 2021, spanning five months, after which chase water flood was run until December 2021. During this PIT, 108,392 barrels of polymer solution in total were injected successfully to acquire a large dataset through sustained monitoring, which was utilized in the assessment of injectivity and in-depth mobility reduction caused by the novel polymer. Initial results showed all of the key performance markers expected, including predictable viscosity yield and desired injectivity at target rates, which were coherent with the data from laboratory investigations. The PIT scheme was optimized with the use of down-hole shut-in tool (DHSIT) to obtain pressure fall-off (PFO) data that brought to light the behavior of the polymer in the near-wellbore area. Consequently, the polymer properties revealed through the PIT is to be used in enhancing field and sector models that will enable the assessment of polymer-based cEOR in myriad other giant, heterogeneous carbonate reservoirs. This is expected to contribute toward higher recovery in ADNOC and Middle Eastern carbonate oil fields [207].
- Another significant study was conducted by Jabbar et al. [204] on a viable sulfonated polymer (ATBS > 35 mol%) for possible employment in a super-giant carbonate field in the Middle East. In this study, polymer rheology and thermal stability were evaluated, and core flooding in a composite reservoir core with live-oil, as well as simulation in a representative sector of the field were performed. The features of the field are defined as highly heterogeneous with low permeability (5–10 mD) and streaks of (200 mD). Furthermore, the formation water has high salinity (i.e., TDS of 180,000 ppm and hardness of 16,000 ppm) and the reservoir temperature of 100 ${}^{{}^{\circ}}$C increased the harshness of the environment. From the simulation, an incremental oil recovery of about 5% was shown when a pre-sheared polymer with a viscosity of 3 cP, concentration of 4000 ppm, and slug size of 0.25 PV was injected. They found the polymer flood effect to be much like that of infill drilling, except with a notable decrease in water production [204].

Thus, according to the reported field applications worldwide, a number of polymer-based EOR were implemented in HTHS reservoirs. The key conclusions that can be derived from these are the performance of the novel polymers, such as SMG (soft micro gel) and HMA polymers that increased recovery factor from 44% to 66% and decreased the water cut decreased from 95% to 54% and the ATBS-based novel polymers with high-tolerance to HTHS carbonate conditions. Polymer-based projects implemented in secondary mode injection have a better success rate than those implemented in tertiary mode. Simultaneous Injection of miscible gas and polymer (SIMGAP) and simultaneous injection of water and polymer (SIWAP), both developed by ADNOC, are aimed at extending the polymer-based EOR project by injecting the ATBS polymer into HTHS carbonate reservoirs that have been flooded. Furthermore, in the super-giant Middle Eastern carbonate field, a new sulfonated polymer with ATBS >35 mol% has been proved to be successful. The pilot projects demonstrate enormous potential for operational plans in the future using polymer-based EOR techniques, with effective recoveries and low water production.

#### 7.1.2. Novel Bio-Polymers

A core-scale numerical study was conducted by Al-Shalabi (2018) on schizophyllan, a bio-polymer in carbonate formations with HTHS conditions. He employed the UTCHEM simulator to highlight the different applied mechanisms in the simulator. Al-Shalabi also highlighted the importance of heterogeneity in history matching for water and polymer core-floods [214]. Furthermore, his demonstrations illustrated that application of polymer flooding during secondary mode of injection is more viable, as it gives results that merit the investment. Given that there are very few feasible and well-established polymers that could effectively function at harsh reservoir conditions, the lack of number of field applications and simulation studies is no anomaly. Nevertheless, looking at recent developments in both synthetic- and bio-polymers, as we have discussed earlier from the exhaustive literature, it can be presumed that it is only a matter of time before new field projects in harsh carbonate reservoirs employing these conventional and novel polymers become common practice. Moreover, the experiences in sandstone reservoirs are indicative of successful implementation of polymers in temperatures up to 100 ${}^{\circ }$C, salinities as high as 350,000 ppm with hardness up to 25,000 ppm [95].

### 7.2. Conventional Polymers Mixed with Surfactants and Alkali

The performance of polymers mixed with alkaline and surfactant was studied using numerical simulation by several researchers. Rai et al. [215] studied the modeling of surfactant and surfactant–polymer (SP) flooding by using CMG-STARS. According to the surfactant flooding simulation, the additional recovery after water-flooding was found to be 17.56%, which was comparable with the experimental result. After history matching with experimental production data, the recovery factor from simulation of SP flooding was found to be 24% more than water flooding [215]. Firozjaii and Moradi [105] studied the effective parameters on the performance of polymer flooding and compared that with ASP flooding, using numerical simulation and experimental design. They found that the effect of different factors is different in polymer flooding compared to that of ASP flooding. They investigated the performance of dodecyl-trimethyl-ammonium bromide DTAB (surfactant), HPAM (polymer), and sodium hydroxide (alkaline) as ASP flooding in numerical simulation after history matching with production data from laboratory work on water-flooding and surfactant injection. The results showed the synergies of mobility control and IFT reduction during ASP with an increase in oil recovery factor compared to surfactant flooding [105]. The screening criteria of polymer flooding will be changed when it is mixed with surfactant or alkaline [11].

#### Field Applications

Two field applications are listed below:

- For SP-based EOR, the pilot tests worldwide include the Russian Tpexozephoe Field, Hungarian H Field, Whittier Field in California, and North Gujarat Oil Field in India [37].
- For PSA-based EOR, large-scale field projects were successful and showed encouraging results. Etzikom in Alberta, Canada and Minas II oil field in Indonesia are two current field projects currently underway [37].

To summarize, based on simulations of several applicable mechanisms, biopolymers such as schizophyllan are possible candidates for carbonate formations under HTHS circumstances. It should be mentioned that the use of polymer flooding during the secondary method of injection is more viable due to the high return on investment. Surfactant flooding recovery factor simulations revealed a 24% higher recovery rate than water flooding. Furthermore, the synergistic effect of mobility control and IFT reduction during ASP boosts the oil recovery factor more than surfactant flooding. As a result, simulation modeling can be useful in determining the performance of SP and ASP cEOR floods, as well as allowing for the selection of the appropriate chemicals and designs based on the needs of the chosen reservoir, such as HTHS carbonate reservoirs.

### 7.3. Polymer-Based Hybrid EOR

Hybrid approaches have the potential to be beneficial in difficult reservoirs and throughout the tertiary stages of residual oil recovery. In high salinity carbonates formations, a synergy of two mechanisms from mixing low-salinity water (i.e., ionically modified brine) or smart water approaches with polymer flooding has proved effective. The efficacy and robustness of the hybrid low-salinity polymer flooding under harsh conditions of carbonate rocks are established in the following sections by decisive laboratory tests, numerical simulations, and field studies.

#### 7.3.1. Low Salinity Polymer (LSP) Flooding

In recent years, a number of experiment-based and modeling studies have been conducted to demonstrate the efficiency of the hybrid low salinity polymer (LSP) technique (i.e., known as engineered water polymer flooding as well). In diverse carbonate formations, studies have indicated that the hybrid LSP approach can achieve up to 30% incremental oil recovery. According to the research on the use of LSP-based EOR in carbonates, the LSP process can boost oil recovery by an amount equivalent to or greater than the sum of the recovery from each technique (i.e., low salinity injection and polymer flooding).

##### Numerical Studies

The simulation assessments of the hybrid low salinity polymer (LSP) approach are listed below.

- Rivet [216] and Seright et al. [101] studied the combined effects of engineered water polymer flooding and highlighted the better polymer stability and yield, improved microscopic and macroscopic sweep efficiency, and reduction in chemical costs [101,216]. A seawater desalination process was developed for combined engineered water and polymer applications in an offshore field [217]. The hybrid engineered water polymer method can achieve up to 30% of OOIP incremental recovery [182].
- Alzayer and Sohrabi [218] conducted a numerical simulation study using a correlation between the residual oil saturation after water flooding (S${}_{orw}$) and the salinity of water [218], as developed by Webb et al. [219] utilizing the relevant published data in the literature [219]. The objective of the study was to improve the oil recovery from a heavy oil reservoir (80 cP and 20${}^{{}^{\circ}}$ API) using low salinity water (LSW) injection followed by polymer flooding (PF). A comparison of the different injection schemes showed that the combination of LSW and PF provided an additional 4% estimated ultimate recovery (EUR) of the original oil in place (OOIP) with significantly lower injection volumes required compared to both methods simulated separately.
- The synergetic effects of hybrid engineered water polymer flooding were also confirmed by the modeling studies conducted by Hirasaki and Pope [220], Han and Lee [221], and Khamees and Flori [222].
- Using a combined MRST-IPhreeqc simulator, Al Shalabi et al. [12] developed a mechanistic model of hybrid low salinity polymer (LSP) flooding. In this study, a coupled numerical model was used to investigate the polymer–brine–rock geochemical interactions as well as the flow dynamics associated with LSP flooding. The geochemical software IPhreeqc, which is the interface module of PHREEQC, was combined with MATLAB Reservoir Simulation Toolbox (MRST). The impacts of polymer on polymer viscosity were captured using the Todd–Longstaff mixing model, inaccessible pore volume, permeability reduction, polymer adsorption, and salinity and shear rate effects [12]. The coupled simulator enables the real-time monitoring of the aqueous phase salinity and its impact on the polymer rheological characteristics, which is advantageous for LSP-based cEOR field applications.

##### Field Application

The very first hybrid low-salinity water polymer-flood pilot test was reported by Dandekar et al., and Ning et al., which began in August 2018 on the Alaska North Shore (ANS) at the Milne Point reservoir, possessing large volumes of heavy-oil reserves [30,223,224,225]. The Milne Point heavy-oil reservoir is of a thin layer and covered with dense permafrost deposit. Due to the risks of heat loss and environmental qualms, thermal-recovery methods were ruled out. Instead, solvent-based methods, in which solvent agents, such as carbon dioxide (CO${}_{2}$), methane, and propane, and/or their mixture are used, were considered to be more conducive for causing in situ oil viscosity reduction and oil recovery enhancement [138,226]. Even then, the high mobility of the displacing solvent agents still posed a challenge in achieving the estimated EOR efficiency if additional measures were not taken. Moreover, the cost of an enormous quantity of fairly expensive solvent had to be utilized, which would have escalated the cost. Additionally, at the initial stage, waterflooding was able to sustain the production; however, premature breakthrough and fast rise of water cut occurred before long [227]. Hence, polymer flooding was deemed to be the effective technique to unlock the heavy-oil resources in this zone, encouraged by reports of several successful field applications of polymer flooding in heavy-oil reservoirs worldwide. For instance, Canada (Pelican Lake, Seal, Cactus Lake), China (Bohai Bay), the Middle East, Suriname (Tambaredjo), and Trinidad and Tobago [228].

Thus, the combined synergic benefits of LSW and polymer flooding were assessed to be the most suitable process at Milne Point. There is also easy availability of LSW resources in the Milne oil field area, which saves the necessity of any additional facilities to be installed. Consequently, the outcome of the almost 2-years of the pilot project in the Milne Point field as on May 2020, showed remarkable outcomes of the proposed hybrid enhanced-oil-recovery (EOR) process, i.e., water-cut reduction was decreased from 70% to less than 15% with oil rate increase and no polymer breakthroughs were observed, mainly attributed to the enhanced sweep efficiency by polymers and the Sor reduction induced by low-salinity in the unswept zones. The flood design had two horizontal injection wells and horizontal producer wells each, further details of which are available in the literature published by Dandekar et al. [229,230] and Ning et al. [223]. It was observed from the pilot test that the decreasing trend in oil rate, which is anticipated during waterflood, was reversed and began to increase owing to the polymer injection. Thus, this most recent field application demonstrates the outstanding performance of hybrid LSW and polymer flooding for heavy-oil recovery improvement [229,230].

To sum it up, simulations comparing different injection schemes show that LSW combined with PF yields a further ultimate recovery (EUR) of 4% OOIP. The results also demonstrate that LSPF requires much smaller injection volumes in comparison to the injection volumes needed for both methods when applied independently. The benefits of LSPF also include the alleviation of water breakthrough problems. Simulations project that the EWPF hybrid method can attain approximately 30% of OOIP incremental recovery, resulting from synergic effects due to enhanced polymer stability and yield capacity as a result of increased microscopic and macroscopic sweep efficiency, leading to low chemical expenditure. In addition, approximately 10% of OOIP is attainable if LSP flooding is conducted directly after waterflooding. Moreover, these results were observed from the first-ever LSPF field project at the Milne oil field, where LSW and polymer flooding successfully increased oil rate, substantially decreased water cut with no early breakthrough and there was a much lower polymer requirement. Hence, an economically viable (polymer consumption is diminished) process for HTHS carbonates, the LSPF/EWPF can induce high oil recovery, with minimal environmental footprint (reduced and water cut), to be one of the important techniques of the imminent EOR applications in harsh carbonate oil fields with high salinity.

## 8. Environmental Impact of Polymer

In recent years, global initiatives have been launched in response to climate concerns, with the goal of lowering CO${}_{2}$ emissions from fossil fuels. Furthermore, the ever-increasing global energy demand has pushed energy production and research efforts to improve low-carbon energy sources and techniques. It is critical to develop and use hydrocarbon fields in a sustainable and energy-efficient manner as the primary energy source is expected to shift from fossil fuel to renewable energy. Hassan et al. [88] devised a process based on the idea of energy return on energy investment (ERoEI) that evaluates the energy efficiency and consequent CO${}_{2}$ footprint of polymer and surfactant cEOR to examine the environmental impact based on the CO${}_{2}$ footprint of polymer-based cEOR [88]. They provided a model for energy analysis to assess the effects of polymer-based EOR on climate change and total environmental impact. The primary energy investment in oil recovery via water injection, for example, is in the water circulation necessary to generate oil. When the water cut (water percentage in total liquid produced) is greater than 90%, the total energy invested in pumping the fluids alone is greater than 70%. As a result, massive amounts of CO${}_{2}$ are generated during the production of oil barrels from old water-flooded oil fields with large water cuts. As a result, the amount of circulated water required to generate oil is the most important energy investment in oil recovery operations by water injection. Furthermore, numerical simulations have estimated that the extra oil recovered by injection of chemicals, is manually obtained from a system at a particular thermodynamics state. In this integrated process, the important surface and subsurface elements of polymer-based chemical EOR techniques are considered. Chemicals injected into the field after the water has been cut can sometimes exceed 90%. As a result, polymer injection into reservoirs with high water cuts, through the period of energy transition, is considered to be a worthy possible solution for the following reasons:

- Satisfying global energy demand using polymer-based cEOR;
- Reduction in the CO${}_{2}$ intensity of produced oil (i.e., larger quantities of oil and cleaner oil).

This ERoEI energy concept allows for the appropriate quantification of the life-cycle efficiency of polymer-based cEOR projects. With the passage of time, the energy recovery factor for cEOR decreases, implying that the method of energy required to produce energy output grows. Practical energy used in the production of hydrolyzed polyacrylamide (HPAM) polymers is estimated to be around 123.6 MJ/kg. For gas, oil, and coal, this results in CO${}_{2}$ emissions of 3.25, 4.72, and 6.35 kg CO${}_{2}$ per kilogram of polymer, respectively. Polymer injection often improves the energy efficiency of an oil recovery system when compared to waterflooding, due to increased oil production (energy gain) and reduced water circulation (in energy investment) [42,88]. Because large volumes of water are consumed, the project time-averaged energy invested to produce one barrel of oil using polymer-based EOR is substantially lower than the extended periods of waterflooding. Some cEOR operations may have larger energy investment than waterflooding; however, because a substantial amount of energy investment is in the form of materials, the CO${}_{2}$ intensity of oil extracted from such projects is relatively low compared to water injection at massive water cutbacks. As a result, polymer injection in reservoirs with a considerable water cut could be a potential solution to the energy transition period’s two major challenges. Furthermore, the total improvement in energy efficacy in surfactant-based EOR is dependent on the excess gain and amount of chemicals in the injected formulation [42,88].

Additionally, HPAM and other synthetic polymers are typically highly expensive, can pollute the environment, and degrade in the HTHS environment. Guar gum is a suitable alternative in this regard because it is both environmentally friendly (biodegradable) and a naturally water-soluble polymer. It is generally available in large amounts in many places of the world, and it is used in a wide range of oil and gas applications. Guar gum is primarily utilized in drilling fluids and hydraulic fracturing operations; however, only a few studies have looked at Guar as a viable candidate polymer for polymer-based cEOR, and no studies on its application in HTHS carbonate reservoirs have been conducted to date. Musa et al. [42] performed a study on guar gum to see if it could be used as a natural polymer in polymer-based cEOR applications for sandstone reservoirs, as well as to comprehensively examine its applicability for HTHS reservoir conditions [42].

Furthermore, to reach net-zero emissions, the global consensus on reducing carbon emissions calls for replacing current fossil-fuel-based energy sources with renewable energy sources, such as wind and solar energy, as well as geological energy, such as hydrogen, as alternate fuels and CO${}_{2}$ sequestration, etc. CO${}_{2}$ sequestration via the carbon capture, utilization, and storage (CCUS) technique is an effective approach for reducing greenhouse gas emissions from EOR applications. Over 60% of the CO${}_{2}$ present in flue gas (used for CO${}_{2}$ flooding process) can be captured and stored as CO${}_{2}$ or CO${}_{2}$-mixed hydrates in depleted reservoirs using CCUS technology. Variables such as injection pressure and the reservoir conditions, such as temperature, pressure, and hydrate saturation, have a substantial impact on CO${}_{2}$ capture efficiency [21]. Similarly, a possible approach of decarbonizing energy sources is to use hydrogen produced by electrolysis using renewable energy or hydrocarbon reformation with carbon capture and storage (CCS) [20]. When combusted, the hydrogen form with an energy density of 141.86 MJ/kg emits no carbon dioxide and can be stored sustainably in geological storage, such as depleted oil fields [21]. As a natural low-carbon energy carrier, hydrogen could eventually replace natural gas for household heating and power generation. Therefore, achieving nominal carbon footprint requires several large-scale approaches and modification of conventional choices of fuel utilisation and storage. Furthermore, concerning EOR procedures, ERoEI energy concept can be tremendously useful in appropriate quantification of the life-cycle efficiency of polymer-based cEOR projects to achieve efficient operations. Moreover, the aged and depleted oil fields can be excellent storage sites for large volumes of hydrogen if repurposed for this, using carbon utilization and storage (CCS) technology, which provides a promising alternative as a green energy storage due to the availability of numerous potential storage sites (i.e., offshore and onshore run-down oil fields) [21].

## 9. Recommended Practices for Polymer Flooding in Harsh Conditions

For a polymer-based EOR project to be deemed successful, the incremental oil recovery must be achieved with the least economic expense (i.e., chemicals cost and water cut) in the shortest amount of time. Due to the unique HTHS environment, heterogeneous formation (i.e., including vugs and natural fractures), and mixed to oil-wet wettability condition, the carbonate reservoir poses various obstacles in polymer-based cEOR applications. For these reasons, meticulous planning and design are required for a successful field implementation of a polymer-based EOR project in severe conditioned carbonate reservoirs. There are a few noteworthy recommendations that will aid operators in the appropriate design and planning of polymer-based EOR projects in carbonate reservoirs under harsh conditions, such as high temperature, high salinity, and low permeability. Several recent screening studies and core-flooding tests have led to these recommendations [42,62,65,67,70,71,88,119,120,128,129,150,155,162,163,164,169,170,171,172,173,175,176], along with the simulation runs and field pilots [42,44,78,88,104,169,176,202,203,204,205,206,207,212] in carbonate rocks. These following recommendations will aid in improved designs for field implementation and are intended to possibly resolve the challenges of high temperature, high salinity/hardness, and injectivity issues in low-permeability carbonate formations. Hence, the key points are as follows:

I. High Temperature

Novel polymers such as sulfonated polymers can remain stable in extremely high temperature reservoirs, which greatly extend the application envelope of cEOR. Although rather expensive, co/ter-polymers containing ATBS and NVP have high thermal stability of up to 140 ${}^{{}^{\circ}}$C in saline solutions and easily last one year at this temperature (Gaillard et al., 2015; Dupuis et al., 2017). However, higher sulfonation levels can lead to increased pore plugging issues [173]. Furthermore, there is found to be great potential in thermo-viscosifying polymers (TVPs) and these would be considerably cheaper if applied, but more tests are required to ascertain the full capacities of these polymers, since these are only partially developed [71,163,164]. Lastly, high temperature is still the biggest hurdle to overcome in polymer-based cEOR.

II. High Salinity/Hardness

Formation water with high salinity/hardness is undesirable, as it causes viscosity loss of the polymer solution and as a result, diminishes the project economics. The way forward in this issue is to use to salinity-resistant polymers such as biopolymers; however, biodegradation severely affects these polymers. Nevertheless, some polymers have been fairly stable in highly saline and hard solutions, namely co/terpolymers of HPAM incorporated with ATBS. One should note that the polymer stability is not only dependent on salinity, but also on temperature. Fresh water injection has been suggested as a useful approach to the high-salinity problem as was seen in the case of the Eliasville Caddo Unit; however, it must be noted that the reservoir temperature was quite low. A pre-flush of fresh water, also termed as low-salinity/engineered water injection, is a potential solution to enhance polymer flood performance in high-salinity reservoirs. These hybrid low salinity water-polymer (LSWP) cEOR mechanisms are indicative of improved recovery rates. It was reported by Al-Shalabi and Sepehrnoori [231,232] that a hybrid LSWP method could help reduce the concentration of polymer needed to reach the appropriate viscosity [231], reducing the project costs, as superior oil recovery efficiency is attained due to enhanced microscopic displacement and macroscopic sweep efficiencies. More details can be gained elsewhere on low salinity/engineered water injection and its hybrid applications [231,232,233,234,235].

III. Low Permeability

If the expenditure of a polymer project is to be lowered, the most straightforward approach is to minimize the amount of polymer used to reach a particular viscosity target. Thus, to achieve this without losing viscosity, it is suggested to utilize a higher-MW (i.e., molecular weight) polymer. However, to avoid injectivity problems, MW should be within the prescribed limits. It is generally advised to use low MW polymers for low-permeability formations. Therefore, to achieve good injectivity, it becomes necessary to meticulously balance MW in an optimization paradox. Pre-shearing polymer solutions may prove to be an effective method, facilitating the solution to retain its apparent viscosity during injection as well as abetting adequate injectivity. The procedure of performing pre-shearing on sulfonated polymers is detailed by Driver et al. [173], which aims to achieve injectivity in very narrow/tight (5-10 mD) carbonate formations. It was demonstrated by them that the conditioning of polymer solutions using the pre-shearing method, tight filtration, and using co-solvents in the case of highly sulfonated polymers would result in very good injectivity [173]. Furthermore, it was reported by Fortenberry et al. [236] that using co-solvents helps in better injectivity of the polymer [236]. Furthermore, it appears to be the case that synthetic polymers are better suited for injection into very narrow/tight formations, as they are not prone to filterability issues that biopolymers are usually affected with [129].

IV. Polymer Adsorption

Adsorption can irreparably damage the reservoir and decrease the polymer flood efficiency. Employing cationic polymers in carbonates can reduce polymer adsorption on rock surface. Moreover, copolymers of AM (i.e., acrylamide nonionic polymer) and AA (i.e., acrylic acid anionic polymer) have been shown to undergo no precipitation and no shearing in oil-wet carbonate reservoirs (i.e., positively charged rock) [123,124,125].

V. Polymerization

Table 3 shows the chemistry of different monomers used to functionalize acrylamide for tolerating harsh conditions, while Table 4 provides a summary of potential polymers for applications in carbonate reservoirs with harsh conditions, wherein temperature, TDS, and hardness conditions refer to stability tests. In Table 4, “Present” denotes that only the presence of the monovalent or divalent ion will cause the polymer to precipitate at the specified temperature, whereas “-” denotes that the presence of the salt has no bearing on whether it will precipitate at the given temperature.

In summary, it is expected that, with the advancing number of research works on optimizing polymer-based cEOR flooding, through the development of a range of robust polymers and effective techniques, the course has been established for imminent field applications to be successful in HTHS carbonate reservoirs.

## 10. Final Remarks

Many of the world’s oil reservoirs are now matured, where polymer-based cEOR techniques have become indispensable for incremental oil retrieval. A large percentage of the hydrocarbon reservoirs worldwide are contained in carbonate formations under harsh conditions, such as high temperature, high salinity (HTHS), especially in the Middle East.

As a consequence of these harsh HTHS conditions, conventional polymer flooding has become ineffective to a great degree due to instability (precipitation), degradation (loss of the viscosifying power), and polymer adsorption. As a result, numerous recent studies have concentrated on developing cEOR techniques with respect to novel polymers, surfactant–polymer (SP) and alkali–surfactant–polymer (ASP) formulations; polymer mixed with other EOR techniques is termed as hybrid polymer solutions. The results from these studies have provided a range of possible approaches to mitigate the challenges concerned in HTHS carbonates during cEOR operations. Since polymers are extensively used in cEOR, it is needless to say that enhancing polymers to become tolerant to harsh conditions is the objective of a sizable number of current research studies. Several recent polymer types viz., ATBS-based and NVP-based polymers, scleroglucan, hydrophobic associative polymers (HPAMs), novel smart thermoviscosifying polymers (TVPs), temperature- and salt-induced viscosifiers, soft micro gel polymers (SMG), sulfonated polymers, and HPAM modifications were analyzed in detail in this review work. Many of these newly engineered polymers (both synthetic and biopolymers) have exhibited excellent tolerance toward HTHS carbonates reservoirs during long-term thermal and salinity tests, core floods, numerical simulations and pilot projects. Researchers, on the other hand, have concentrated on developing surfactant–polymer and alkali–surfactant–polymer formulations that can withstand the thermal instability, degradation, and adsorption issues associated with HTHS conditions in carbonate rocks while maintaining the required viscosity for long periods of time. Researchers have also worked to improve the synergy effects of polymer-based hybrid EOR combination procedures, such as low-salinity water–polymer (LSWP) injection and polymer-enhanced CO${}_{2}$ foam flooding in difficult settings to improve oil recovery rates and overall performance.

It is also worth noting that the issue of climate change has risen to the forefront of global environmental legislation. As a result, CO${}_{2}$ emissions (i.e., CO${}_{2}$ footprint) connected with cEOR may become the most important criterion for selecting polymers. By the second half of the century, the world’s energy dependency will have moved from hydrocarbon fossil fuel energy to renewable energy, with the goal of reaching net-zero carbon emissions. As a result, considering and implementing environmentally acceptable solutions into the oil industry’s key developmental plans has become a vital aspect of technological growth for the industry’s long-term viability. Due to its biodegradable and non-ionic (low adsorption on the surface of carbonate rock) features, biopolymers such as schizophyllan have proved to be the most high-potential green polymers for HTHS condition for both sandstone and carbonate rocks, according to various studies.

Although large-scale manufacture of this polymer is not currently economically practical, it is a viable option worth considering. Gaur gum has been found as a promising biopolymer that is high-temperature tolerant and has a lower environmental impact than synthetic polymers in a few studies. Its use in carbonates is still being researched; however, it is a very realistic choice for future environmentally friendly polymers. Aside from that, low-cost, low-adsorption synthetic polymers, as well as hybrid approaches, such as LSWF, which reduce polymer usage, are potential environmentally responsible options. As a result of the combined efforts of research works based on experimental and field applications, a progressive trend toward expanding polymer-based cEOR toward HTHS has emerged.

## Figures and Tables

**Table 1:** Updated screening criteria for polymer flooding (Data are taken from Antoine Thomas, 2016 [90]).

Parameter	Year 1970s and 1980s	After Year 2000
Oil viscosity	<200 cP	<10,000 cP
Temperature	<95 ${}^{{}^{\circ}}$C	<140 ${}^{{}^{\circ}}$C
Permeability	>20 mD	>10 mD
Salinity Low	(<30 g/L TDS)	<200 g/L TDS

**Table 2:** A list of 733 polymer-flooding projects worldwide (Adopted from Sheng, 2015 [56]).

Country Name	Number of Field Application	Country Name	Number of Field Application
Angola	1	Indonesia	1
Argentina	11	Kuwait	1
Austria	1	Mexico	1
Australia	1	Nigeria	1
Brazil	2	Oman	2
Canada	50	Poland	1
China	67	Romania	3
Columbia	1	Russia	2
France	5	Suriname	1
Germany	12	Trinidad	1
Hungary	1	UK	1
India	6	USA	560

**Table 3:** Chemistry of different monomers used to functionalize acrylamide for sustaining harsh conditions [8].

Monomer	Chemical Name	Charge	Main Function
AA	Acrylic acid	Anionic	Limits hydrolysis and thermal degradation, also a product of AM hydrolysis
AM	Acrylamide	Nonionic	The main constituent of synthetic EOR polymers
AMPS	2-Acrylamido-2-MethylPropane-Sulfonate	Anionic	Increases tolerance to salinity
ATBS	Acrylamide-Tertio-Butyl Sulfonate	Anionic	Limits hydrolysis and thermal degradation
HPAM	Partially Hydrolyzed Polyacrylamide	Anionic	The partially hydrolyzed form of polyacrylamide
PAM	Polyacrylamide	Nonionic	Hydrolyzed form of polyacrylamide
NVP	N-Vinyl-Pyrrolidone	Nonionic	Limits hydrolysis and thermal degradation

**Table 4:** Summary of polymers temperature and salinity limits [8].

Polymer	Temperature	TDS	Hardness	Retained Viscosity	Tested on Core-Flood
Synthetic Polymers					
AM	75	-	Present	Precipitates	Yes
AMPS	120	200	18	180 days	No
ATBS	110	20	-	60 days	No
HPAM	110	50	-	60 days	No
PAM	120	167	46	90% after 100 days	Yes
NVP	140	84.5	6	60% after 365 days	No
SAV37	105	84.5	6	60% after 365 days	No
SAV333	140	84.5	6	90% after 365 days	No
SAV333	120	79.9	27	60% after 365 days	Yes
SAV225	105	79.9	27	60% after 365 days	Yes
AM-ATBS copolymer	130	230	20.8	70% for 365 days	No
Biopolymers					
Xanthan	90	-	-	Precipitates	Yes
Schizophyllan	120	201.6	56.3	240 days	Yes
Scleroglucan	100	30	Present	730 days	Yes
Guar-gum	100	200,000	-	-	Yes

## Abbreviations

**Symbols**
$E_{A}$	areal sweep efficiency
$E_{D}$	displacement efficiency
$E_{I}$	vertical sweep efficiency
$E_{V}$	volumetric sweep efficiency
k	absolute permeability
$k_{o}$	effective permeability of oil
$k_{w}$	effective permeability of water
M	mobility ratio
$N_{De}$	Deborah number
n	shear-thinning index
$S_{w}$	water saturation
**Greek letters**
$\tau_{E}$	characteristic residence time
$\tau_{r}$	relaxation time of the polymeric fluid
λ	phase mobility
μ	viscosity
**Subscripts/Superscripts**
*i*	initial
*o*	oil
*w*	water
**Abbreviations**
AA	Acrylic Acid
AM	Acrylamide
AMPS	2-Acrylamido-2-Methylpropane Sulfonic Acid
AN	(Sulfonated) Polyacrylamide
AOS	(Sodium) Alpha Olefin Sulfate
ATBS	Acrylamide Tertiary Butyl Sulfonic Acid
AVS	Amphiphilic Ter-polymer of AM, AMPS
BEM	Butylethyl Magnesium
CEOR	Chemical Enhanced Oil Recovery
CMG-STARS	Computer Modeling Group Ltd
CO${}_{2}$	Carbon Dioxide
DOH	Degree of Hydrolysis
DTAB	Dodecyl-Trimethyl-Ammonium Bromide
EOR	Enhanced Oil Recovery
ERoEI	Exergy-Return on Exergy-Investment
WE	Engineered Water
EWF	Engineered Water Flooding
EWPF	Engineered Water Polymer Flooding
HAP	Hydrophobically Associating Polymers
HEC	Hydroxyethylcellulose
HMA	Hot-melt Adhesives
HMA	Hydrophobically Modified Associating
HMA-PAM	Hydrophobically Modified Associating Polymers-Polyacrylamide
HPAM	Hydrolyzed Polyacrylamide
HTHS	High Temperature High Salinity
IFT	Interfacial Tension
IOR	Improved Oil Recovery
IW	Injection Water
LCST	Lower Critical Solution Temperature
LSPF	Low-Salinity Polymer Flooding
LSW	Low-Salinity Water
LSWF	Low-Salinity Water Flooding
LSW-P	Low-Salinity Water Polymer
MW	Molecular Weight
MWCNT	Multi-Walled Carbon Nanotubes
NaCl	Sodium Chloride
NMR	Nuclear Magnetic Resonance
NVP	N-Vinylpyrrolidone
OOIP	Original Oil in place
PAM	Polyacrylamide
PEF	Polymer-Enhanced Foam
PF	Polymer Flooding
PS	Polymer–Surfactant
PSA	Polymer–Surfactant–Alkaline
PSF	Polymer–Surfactant Flooding
PV	Pore Volume
PZC	Point of Zero Charge
RE	Recovery Efficiency
SDBS	Sodium Dodecyl Benzene Sulfonate
SIMGAP	Simultaneous Injection of Miscible Gas and Polymer
SIWAP	Simultaneous Injection of Water and Polymer
SMG	Soft Micro Gel Polymers
SP	Surfactant–Polymer
STARPAM	Star-shaped Polymer of Polyacrylamide
TDS	Total Dissolved Solids
TVP	Thermo-Viscosifying Polymers
UTCHEM	University of Texas Chemical Simulator
VP	Vinylpyrrolidone

## References

[1] Bob D. (2018). BP Energy Outlook.

[2] BP Energy Economics. BP Statistical Review of World Energy. https://www.imemo.ru/files/File/ru/events/2021/BP-2021.pdf.

[3] British Petroleum BP Statistical Review of World Energy 2020: A Pivotal Moment. https://www.bp.com/content/dam/bp/business-sites/en/global/corporate/pdfs/energy-economics/statistical-review/bp-stats-review-2021-full-report.pdf.

[4] Kokal S., Al-Kaabi A. (2010). Enhanced Oil Recovery: Challenges & Opportunities.

[5] Roehl P.O., Choquette P.W. (2012). Carbonate Petroleum Reservoirs.

[6] Akbar M., Vissapragada B., Alghamdi A.H., Allen D., Herron M., Carnegie A., Dutta D., Olesen J.R., Chourasiya R., Logan D. (2000). A snapshot of carbonate reservoir evaluation. Oilfield Rev..

[7] Cuiec L. (1984). Rock/crude-oil interactions and wettability: An attempt to understand their interrelation. SPE Annual Technical Conference and Exhibition.

[8] Diab W.N., Al-Shalabi E.W. (2019). Recent developments in polymer flooding for carbonate reservoirs under harsh conditions. Offshore Technology Conference Brasil.

[9] Lake L.W. (1989). Enhanced Oil Recovery.

[10] Stone H., Garder A. (1961). Analysis of gas-cap or dissolved-gas drive reservoirs. Soc. Pet. Eng. J..

[11] Taber J.J., Martin F.D., Seright R. (1997). EOR screening criteria revisited-Part 1: Introduction to screening criteria and enhanced recovery field projects. SPE Reserv. Eng..

[12] Al-Shalabi E.W., Alameri W., Hassan A.M. (2022). Mechanistic modeling of hybrid low salinity polymer flooding: Role of geochemistry. J. Pet. Sci. Eng..

[13] Hassan A., Ayoub M., Eissa M., Bruining H., Al-Mansour A., Al-Guraishi A. (2020). A Novel Hybrid Enhanced Oil Recovery Method by Smart Water-Injection and Foam-Flooding in Carbonate Reservoirs. SPE/IATMI Asia Pacific Oil & Gas Conference and Exhibition.

[14] Hassan A.M., Ayoub M., Eissa M., Bruining H., Zitha P. (2020). Study of surface complexation modeling on a novel hybrid enhanced oil recovery (EOR) method; smart-water assisted foam-flooding. J. Pet. Sci. Eng..

[15] Hassan A., Ayoub M., Eissa M., Bruining H., Al-Mansour A., Al-Quraishi A. (2021). A New Hybrid Improved and Enhanced Oil Recovery IOR/EOR Process Using Smart Water Assisted Foam SWAF Flooding in Carbonate Rocks; A Laboratory Study Approach. International Petroleum Technology Conference.

[16] Hassan A.M., Ayoub M., Eissa M., Al-Shalabi E.W., Almansour A., Alquraishi A. (2022). Foamability and Foam Stability Screening for Smart Water Assisted Foam Flooding: A New Hybrid EOR Method. International Petroleum Technology Conference.

[17] Hassan A.M., Ayoub M.A., Mohyadinn M.E., Al-Shalabi E.W., Alakbari F.S. (2022). A New Insight into Smart Water Assisted Foam SWAF Technology in Carbonate Rocks using Artificial Neural Networks ANNs. Offshore Technology Conference Asia.

[18] Hassan A.M., Ayoub M.A., Mohyadinn M.E., Al-Shalabi E.W., Al-Mansour A., Alquraishi A. (2022). Increasing Reservoir Recovery Efficiency through Laboratory-Proven Hybrid Smart Water-Assisted Foam (SWAF) Flooding in Carbonate Reservoirs. Energies.

[19] Babadagli T. (2007). Development of mature oil fields—A review. J. Pet. Sci. Eng..

[20] Turner J.A. (2004). Sustainable hydrogen production. Science.

[21] Hassanpouryouzband A., Yang J., Tohidi B., Chuvilin E., Istomin V., Bukhanov B., Cheremisin A. (2018). CO_2_ capture by injection of flue gas or CO_2_–N_2_ mixtures into hydrate reservoirs: Dependence of CO_2_ capture efficiency on gas hydrate reservoir conditions. Environ. Sci. Technol..

[22] Lwisa E. Chemical Enhanced Oil Recovery. https://www.researchgate.net/profile/Essa-Lwisa/publication/352241416_Chemical_Enhanced_Oil_Recovery/links/60d18fce92851ca3acbb2612/Chemical-Enhanced-Oil-Recovery.pdf.

[23] Reiter P.K. (1961). A Water-Sensitive Sandstone Flood Using Low Salinity Water. Master’s Thesis.

[24] Bernard G.G. (1967). Effect of floodwater salinity on recovery of oil from cores containing clays. SPE California Regional Meeting.

[25] Hallenbeck L., Sylte J., Ebbs D., Thomas L. (1991). Implementation of the Ekofisk field waterflood. SPE Form. Eval..

[26] Romanuka J., Hofman J., Ligthelm D.J., Suijkerbuijk B., Marcelis A., Oedai S., Brussee N., van der Linde A., Aksulu H., Austad T. (2012). Low salinity EOR in carbonates. SPE Improved Oil Recovery Symposium.

[27] Shariatpanahi S.F., Strand S., Austad T. (2011). Initial wetting properties of carbonate oil reservoirs: Effect of the temperature and presence of sulfate in formation water. Energy Fuels.

[28] Aghaeifar Z., Strand S., Austad T., Puntervold T., Aksulu H., Navratil K., Storas S., Hamsø D. (2015). Influence of formation water salinity/composition on the low-salinity enhanced oil recovery effect in high-temperature sandstone reservoirs. Energy Fuels.

[29] Morrow N., Buckley J. (2011). Improved oil recovery by low-salinity waterflooding. J. Pet. Technol..

[30] Jadhunandan P.P. (1990). Effects of Brine Composition, Crude Oil, and Aging Conditions on Wettability and Oil Recovery. Ph.D. Thesis.

[31] Nasralla R.A., Alotaibi M.B., Nasr-El-Din H.A. (2011). Efficiency of oil recovery by low salinity water flooding in sandstone reservoirs. SPE Western North American Region Meeting.

[32] Firozjaii A.M., Saghafi H.R. (2020). Review on chemical enhanced oil recovery using polymer flooding: Fundamentals, experimental and numerical simulation. Petroleum.

[33] Green D.W., Willhite G.P. (1998). Enhanced Oil Recovery.

[34] Lake L.W., Johns R., Rossen B., Pope G.A. (2014). Fundamentals of Enhanced Oil Recovery.

[35] Brownell L.E., Katz D.L. (1947). Flow of fluids through porous media. 1. Single homogeneous fluids. Chem. Eng. Prog..

[36] Rellegadla S., Prajapat G., Agrawal A. (2017). Polymers for enhanced oil recovery: Fundamentals and selection criteria. Appl. Microbiol. Biotechnol..

[37] Ragab A., Mansour E.M. (2021). Enhanced Oil Recovery: Chemical Flooding. Geophysics and Ocean Waves Studies.

[38] Shakeel M., Pourafshary P., Rehan Hashmet M. (2020). Hybrid Engineered Water–Polymer Flooding in Carbonates: A Review of Mechanisms and Case Studies. Appl. Sci..

[39] Chieng Z., Mohyaldinn M.E., Hassan A., Bruining H. (2020). Experimental investigation and performance evaluation of modified viscoelastic surfactant (VES) as a new thickening fracturing fluid. Polymers.

[40] Li X., Zhang F., Liu G. (2021). Review on polymer flooding technology. IOP Conference Series: Earth and Environmental Science.

[41] Karimov D., Hashmet M.R., Pourafshary P. (2020). A laboratory study to optimize ion composition for the hybrid low salinity water/polymer flooding. Offshore Technology Conference Asia.

[42] Musa T.A., Ibrahim A.F., Nasr-El-Din H.A., Hassan A. (2021). New insights into guar gum as environmentally friendly polymer for enhanced oil recovery in high-salinity and high-temperature sandstone reservoirs. J. Pet. Explor. Prod..

[43] Kamal M.S., Sultan A.S., Al-Mubaiyedh U.A., Hussein I.A. (2015). Review on polymer flooding: Rheology, adsorption, stability, and field applications of various polymer systems. Polym. Rev..

[44] Jouenne S. (2020). Polymer flooding in high temperature, high salinity conditions: Selection of polymer type and polymer chemistry, thermal stability. J. Pet. Sci. Eng..

[45] Li J., Liu W., Sun L., Cong S., Jia R., Zhang J., Yang Y. (2019). Effect of Emulsification on Surfactant Partitioning in Surfactant-Polymer Flooding. J. Surfactants Deterg..

[46] Guilyardi E., Lescarmontier L., Matthews R., Point S.P., Rumjaun A.B., Schlüpmann J., Wilgenbus D. (2018). IPCC Special Report “Global Warming of 1.5 ^∘^C”: Summary for Teachers.

[47] Wu D., Hao J., Wang W., Yu Y., Fu X.Z., Luo J.L. (2021). Energy-saving H2 Generation Coupled with Oxidative Alcohol Refining over Bimetallic Phosphide Ni2P- CoP Junction Bifunctional Electrocatalysts. ChemSusChem.

[48] Abe J.O., Popoola A., Ajenifuja E., Popoola O. (2019). Hydrogen energy, economy and storage: Review and recommendation. Int. J. Hydrog. Energy.

[49] Mouli-Castillo J., Wilkinson M., Mignard D., McDermott C., Haszeldine R.S., Shipton Z.K. (2019). Inter-seasonal compressed-air energy storage using saline aquifers. Nat. Energy.

[50] Scafidi J., Wilkinson M., Gilfillan S.M., Heinemann N., Haszeldine R.S. (2021). A quantitative assessment of the hydrogen storage capacity of the UK continental shelf. Int. J. Hydrogen Energy.

[51] Heinemann N., Alcalde J., Miocic J.M., Hangx S.J., Kallmeyer J., Ostertag-Henning C., Hassanpouryouzband A., Thaysen E.M., Strobel G.J., Schmidt-Hattenberger C. (2021). Enabling large-scale hydrogen storage in porous media—The scientific challenges. Energy Environ. Sci..

[52] Silva I.G., de Melo M.A., Luvizotto J.M., Lucas E.F. (2007). Polymer flooding: A sustainable enhanced oil recovery in the current scenario. Latin American & Caribbean Petroleum Engineering Conference.

[53] Algharaib M., Alajmi A., Gharbi R. (2014). Improving polymer flood performance in high salinity reservoirs. J. Pet. Sci. Eng..

[54] Klimenko A., Molinier V., Dubos F., Joly M., Saint-Loubert M., Jouenne S., Passade-Boupat N., Bourrel M. (2020). Surfactant–Polymer Flooding at High Temperature and High Salinity: Promising Lab Scale Experiments in Challenging Conditions. Abu Dhabi International Petroleum Exhibition & Conference.

[55] Telmadarreie A., Trivedi J.J. (2018). Static and dynamic performance of wet foam and polymer-enhanced foam in the presence of heavy oil. Colloids Interfaces.

[56] Sheng J.J. (2015). Status of surfactant EOR technology. Petroleum.

[57] Samanta A., Bera A., Ojha K., Mandal A. (2010). Effects of alkali, salts, and surfactant on rheological behavior of partially hydrolyzed polyacrylamide solutions. J. Chem. Eng. Data.

[58] Sorbie K.S. (2013). Polymer-Improved Oil Recovery.

[59] Xu X., Saeedi A., Rezaee R.a., Liu K. (2015). Investigation on a novel polymer with surface activity for polymer enhanced CO_2_ foam flooding. SPE International Symposium on Oilfield Chemistry.

[60] Seright R.S., Fan T., Wavrik K., de Carvalho Balaban R. (2011). New insights into polymer rheology in porous media. SPE J..

[61] Zaitoun A., Makakou P., Blin N., Al-Maamari R.S., Al-Hashmi A., Abdel-Goad M., Al-Sharji H.H. (2012). Shear stability of EOR polymers. SPE J..

[62] Hashmet M.R., Qaiser Y., Mathew E.S., AlAmeri W., AlSumaiti A.M. (2017). Injection of polymer for improved sweep efficiency in high temperature high salinity carbonate reservoirs: Linear X-ray aided flood front monitoring. SPE Kingdom of Saudi Arabia Annual Technical Symposium and Exhibition.

[63] Unsal E., Ten Berge A., Wever D. (2018). Low salinity polymer flooding: Lower polymer retention and improved injectivity. J. Pet. Sci. Eng..

[64] Han M., Zhou X., Alhasan F.B., Zahrani B., AlSofi A.M. (2012). Laboratory investigation of the injectivity of sulfonated polymer solutions into carbonate reservoir rocks. SPE EOR Conference at Oil and Gas West Asia.

[65] Alfazazi U., AlAmeri W., Hashmet M.R. (2019). Experimental investigation of polymer flooding with low-salinity preconditioning of high temperature–high-salinity carbonate reservoir. J. Pet. Explor. Prod. Technol..

[66] Levitt D., Pope G.A. (2008). Selection and screening of polymers for enhanced-oil recovery. SPE Symposium on Improved oil Recovery.

[67] Gaillard N., Giovannetti B., Favero C., Caritey J.P., Dupuis G., Zaitoun A. (2014). New water soluble anionic NVP acrylamide terpolymers for use in harsh EOR conditions. SPE Improved Oil Recovery Symposium.

[68] Sorbie K. (1991). Polymer-Improved Oil Recovery, 115 Glasgow.

[69] Thomas R., Morgenthaler L. (1999). Introduction to matrix treatments. Reservoir Stimulation.

[70] Alfazazi U., AlAmeri W., Hashmet M.R. (2018). Screening of new HPaM base polymers for applications in high temperature and high salinity carbonate reservoirs. Abu Dhabi International Petroleum Exhibition & Conference.

[71] Gaillard N., Giovannetti B., Leblanc T., Thomas A., Braun O., Favero C. (2015). Selection of customized polymers to enhance oil recovery from high temperature reservoirs. SPE Latin American and Caribbean Petroleum Engineering Conference.

[72] Sheng J.J. (2010). Modern Chemical Enhanced Oil Recovery: Theory and Practice.

[73] Taber J.J. (1983). Technical screening guides for the enhanced recovery of oil. SPE Annual Technical Conference and Exhibition.

[74] Goodlett G., Honarpour M., Chung F., Sarathi P. (1986). The role of screening and laboratory flow studies in EOR process evaluation. SPE Rocky Mountain Regional Meeting.

[75] Gao C.H. (2011). Scientific research and field applications of polymer flooding in heavy oil recovery. J. Pet. Explor. Prod. Technol..

[76] Kang P.S., Lim J.S., Huh C. (2014). Screening criteria for application of EOR processes in offshore fields. Proceedings of the Twenty-Fourth International Ocean and Polar Engineering Conference.

[77] Saleh L.D., Wei M., Bai B. (2014). Data analysis and updated screening criteria for polymer flooding based on oilfield data. SPE Reserv. Eval. Eng..

[78] Standnes D.C., Skjevrak I. (2014). Literature review of implemented polymer field projects. J. Pet. Sci. Eng..

[79] Saboorian-Jooybari H., Dejam M., Chen Z. (2015). Half-century of heavy oil polymer flooding from laboratory core floods to pilot tests and field applications. SPE Canada Heavy Oil Technical Conference.

[80] Zhang Y., Wei M., Bai B., Yang H., Kang W. (2016). Survey and data analysis of the pilot and field polymer flooding projects in China. SPE Improved Oil Recovery Conference.

[81] Rivas C., Gathier F. (2013). C-EOR projects–offshore challenges. Proceedings of the Twenty-Third International Offshore and Polar Engineering Conference.

[82] Chapman E., Mercer D., Jerauld G., Shields R., Sorbie K., Mogford D., Cable A. (2015). Polymer flooding for EOR in the Schiehallion Field-porous flow rheological studies of high molecular weight polymers. Proceedings of the IOR 2015—18th European Symposium on Improved Oil Recovery.

[83] Poulsen A., Shook G.M., Jackson A., Ruby N., Charvin K., Dwarakanath V., Thach S., Ellis M. (2018). Results of the UK Captain Field interwell EOR pilot. SPE Improved Oil Recovery Conference.

[84] Wang W., Lu X. (2009). Property evaluation of EOR technology by means of expansive granular crosslinked polymer. SPE Production and Operations Symposium.

[85] Jouenne S., Chakibi H., Levitt D. (2018). Polymer stability after successive mechanical-degradation events. SPE J..

[86] Seright R.S. (1983). The effects of mechanical degradation and viscoelastic behavior on injectivity of polyacrylamide solutions. Soc. Pet. Eng. J..

[87] Kaminsky R.D., Wattenbarger R.C., Szafranski R.C., Coutee A. (2007). Guidelines for polymer flooding evaluation and development. IPTC 2007: International Petroleum Technology Conference.

[88] Hassan A.M., Ayoub M., Eissa M., Musa T., Bruining H., Farajzadeh R. (2019). Exergy return on exergy investment analysis of natural-polymer (Guar-Arabic gum) enhanced oil recovery process. Energy.

[89] Farajzadeh R., Kahrobaei S., De Zwart A., Boersma D. (2019). Life-cycle production optimization of hydrocarbon fields: Thermoeconomics perspective. Sustain. Energy Fuels.

[90] Thomas A. (2016). Polymer Flooding. Chemical Enhanced Oil Recovery (cEOR)—A Practical Overview.

[91] Han M., AlSofi A., Fuseni A., Zhou X., Hassan S. (2013). Development of chemical EOR formulations for a high temperature and high salinity carbonate reservoir. IPTC 2013: International Petroleum Technology Conference.

[92] Wever D., Picchioni F., Broekhuis A. (2011). Polymers for enhanced oil recovery: A paradigm for structure—Property relationship in aqueous solution. Prog. Polym. Sci..

[93] Lee V.B. (2015). The Development and Evaluation of Polymers for Enhanced Oil Recovery. Ph.D. Thesis.

[94] Pu W., Shen C., Wei B., Yang Y., Li Y. (2018). A comprehensive review of polysaccharide biopolymers for enhanced oil recovery (EOR) from flask to field. J. Ind. Eng. Chem..

[95] Delamaide E. (2018). Polymers and their limits in temperature, salinity and hardness: Theory and practice. SPE Asia Pacific Oil and Gas Conference and Exhibition.

[96] Scott A.J., Romero-Zerón L., Penlidis A. (2020). Evaluation of polymeric materials for chemical enhanced oil recovery. Processes.

[97] Fu M.L., Zhou J.C., Xiong F., YE C., WANG R.H. (2010). A Study on Formation Plugging Mechanism of Cross linked Polymer Flooding in Henan Oilfield. Oilfield Chem..

[98] Chang H., Zhang Z., Wang Q., Xu Z., Guo Z., Sun H., Cao X., Qiao Q. (2006). Advances in polymer flooding and alkaline/surfactant/polymer processes as developed and applied in the People’s Republic of China. J. Pet. Technol..

[99] Levitt D., Klimenko A., Jouenne S., Chamerois M., Bourrel M. (2013). Overcoming design challenges of chemical EOR in high-temperature, high salinity carbonates. SPE Middle East Oil and Gas Show and Conference.

[100] Moradi-Araghi A., Cleveland D., Westerman I. (1987). Development and evaluation of eor polymers suitable for hostile environments: II-Copolymers of acrylamide and sodium AMPS. SPE International Symposium on Oilfield Chemistry.

[101] Seright R.S., Campbell A., Mozley P., Han P. (2010). Stability of partially hydrolyzed polyacrylamides at elevated temperatures in the absence of divalent cations. SPE J..

[102] Zaitoun A., Potie B. (1983). Limiting conditions for the use of hydrolyzed polyacrylamides in brines containing divalent ions. SPE Oilfield and Geothermal Chemistry Symposium.

[103] Wang G., Yi X., Feng X., Jing B., Ouyang J. (2012). Synthesis and study of a new copolymer for polymer flooding in high-temperature, high-salinity reservoirs. Chem. Technol. Fuels Oils.

[104] Wu X., Yang Z., Xu H., Zhang L., Xiong C., Yang H., Shao L., Kang B., Fu Y., Tian X. (2016). Success and lessons learned from polymerflooding a ultra high temperature and ultra high salinity oil reservoir-A case study from west China. SPE Improved Oil Recovery Conference.

[105] Firozjaii A.M., Moradi S. (2018). Sensitivity analysis and optimization of the effective parameters on ASP flooding compared to polymer flooding using CMG-STARS. J. Pet. Environ. Biotechnol..

[106] Khune G., Donaruma L., Hatch M., Kilmer N., Shepitka J., Martin F. (1985). Modified acrylamide polymers for enhanced oil recovery. J. Appl. Polym. Sci..

[107] Sabhapondit A., Borthakur A., Haque I. (2003). Characterization of acrylamide polymers for enhanced oil recovery. J. Appl. Polym. Sci..

[108] Dimitrov I., Trzebicka B., Müller A.H., Dworak A., Tsvetanov C.B. (2007). Thermosensitive water-soluble copolymers with doubly responsive reversibly interacting entities. Prog. Polym. Sci..

[109] Wang Y., Lu Z.Y., Han Y.G., Feng Y.J., Tang C.L. (2011). A novel thermoviscosifying water-soluble polymer for enhancing oil recovery from high-temperature and high-salinity oil reservoirs. Advanced Materials Research.

[110] Wu Y., Liu X., Wang Y., Guo Z., Feng Y. (2012). Synthesis and Aggregation Behaviors of Well-Defined Thermoresponsive Pentablock Terpolymers with Tunable LCST. Macromol. Chem. Phys..

[111] Shepitka J., Case C., Donaruma L., Hatch M., Kilmer N., Khune G., Martin F., Ward J., Wilson K. (1983). Partially imidized, water-soluble polymeric amides. I. Partially imidized polyacrylamide and polymethacrylamide. J. Appl. Polym. Sci..

[112] Liu J.F., Feng J.Y., Yang S.Z., Gang H.Z., Mu B.Z. (2020). The recovery of viscosity of HPAM solution in presence of high concentration sulfide ions. J. Pet. Sci. Eng..

[113] Davison P., Mentzer E. (1982). Polymer flooding in North Sea reservoirs. Soc. Pet. Eng. J..

[114] Moradi-Araghi A., Doe P.H. (1987). Hydrolysis and precipitation of polyacrylamides in hard brines at elevated temperatures. SPE Reserv. Eng..

[115] Maitin B. (1992). Performance analysis of several polyacrylamide floods in North German oil fields. SPE/DOE Enhanced Oil Recovery Symposium.

[116] Berge A., Lenchenkov N., Wever D., Farajzadeh R., Al-Mjeni R., Glasbergen G. (2018). The role of synthetic polymer on rock-fluid interactions and the resulting change in ionic composition and viscosity of the polymer slug for a ceor flood in the sultanate of oman. SPE EOR Conference at Oil and Gas West Asia.

[117] Pope G., Nelson R. (1978). A chemical flooding compositional simulator. Soc. Pet. Eng. J..

[118] Ghannam M.T., Esmail M.N. (1998). Rheological properties of aqueous polyacrylamide solutions. J. Appl. Polym. Sci..

[119] Quadri S.M., Jiran L., Shoaib M., Hashmet M.R., AlSumaiti A.M., Alhassan S.M. (2015). Application of biopolymer to improve oil recovery in high temperature high salinity carbonate reservoirs. Abu Dhabi International Petroleum Exhibition and Conference.

[120] Quadri S.M.R., Shoaib M., AlSumaiti A.M., Alhassan S.M. (2015). Screening of polymers for EOR in high temperature, high salinity and carbonate reservoir conditions. International Petroleum Technology Conference.

[121] Liu Y., Jessop P.G., Cunningham M., Eckert C.A., Liotta C.L. (2006). Switchable surfactants. Science.

[122] Sarsenbekuly B., Kang W., Fan H., Yang H., Dai C., Zhao B., Aidarova S.B. (2017). Study of salt tolerance and temperature resistance of a hydrophobically modified polyacrylamide based novel functional polymer for EOR. Colloids Surfaces A Physicochem. Eng. Asp..

[123] Xu Y., Gao P., Yang M., Huang G., Wang B. (2011). Synthesis and aqueous solution properties of a novel nonionic, amphiphilic comb-type polyacrylamide. J. Macromol. Sci. Part B.

[124] Zhao Y., Zhou J., Xu X., Liu W., Zhang J., Fan M., Wang J. (2009). Synthesis and characterization of a series of modified polyacrylamide. Colloid Polym. Sci..

[125] Zhu Y., Wang Z., Wu K., Hou Q., Long H. (2013). Enhanced oil recovery by chemical flooding from the biostromal carbonate reservoir. SPE Enhanced Oil Recovery Conference.

[126] Doe P.H., Moradi-Araghi A., Shaw J.E., Stahl G.A. (1987). Development and evaluation of EOR polymers suitable for hostile environments part 1: Copolymers of vinylpyrrolidone and acrylamide. SPE Reserv. Eng..

[127] Bridgewater J., Pace S., Gardner G., Schulz D. (1987). Enhanced Oil Recovery with Hydrophobically Associating Polymers Containing N-Vinyl-Pyrrolidone Functionality.

[128] Gaillard N., Giovannetti B., Favero C. (2010). Improved oil recovery using thermally and chemically protected compositions based on co-and ter-polymers containing acrylamide. SPE Improved Oil Recovery Symposium.

[129] Kulawardana E.U., Koh H., Kim D.H., Liyanage P.J., Upamali K.A., Huh C., Weerasooriya U., Pope G.A. (2012). Rheology and transport of improved EOR polymers under harsh reservoir conditions. SPE Improved Oil Recovery Symposium.

[130] Vermolen E., Van Haasterecht M., Masalmeh S.K., Faber M.J., Boersma D.M., Gruenenfelder M. (2011). Pushing the envelope for polymer flooding towards high-temperature and high-salinity reservoirs with polyacrylamide based ter-polymers. SPE Middle East Oil and Gas Show and Conference.

[131] Uhl J., Ching T., Bae J. (1995). A laboratory study of new, surfactant-containing polymers for high-salinity reservoirs. SPE Adv. Technol. Ser..

[132] Kathmann E.E., McCormick C.L. (1997). Water-soluble polymers. 71. pH responsive behavior of terpolymers of sodium acrylate, acrylamide, and the zwitterionic monomer 4-(2-acrylamido-2-methylpropanedimethylammonio) butanoate. J. Polym. Sci. Part A: Polym. Chem..

[133] Fernandez I. (2005). Evaluation of cationic water-soluble polymers with improved thermal stability. SPE International Symposium on Oilfield Chemistry.

[134] Zou C., Zhao P., Ge J., Lei Y., Luo P. (2012). *β*-Cyclodextrin modified anionic and cationic acrylamide polymers for enhancing oil recovery. Carbohydr. Polym..

[135] Argillier J.F., Audibert A., Lecourtier J., Moan M., Rousseau L. (1996). Solution and adsorption properties of hydrophobically associating water-soluble polyacrylamides. Colloids Surfaces A Physicochem. Eng. Asp..

[136] Lijian D., Biao W. (1995). Hydrophobically associating terpolymer and its complex with a stabilizer in brine for enhanced oil recovery. SPE International Symposium on Oilfield Chemistry.

[137] Jiang W., Kang X., Xie K., Lu X. (2013). Degree of the association of hydrophobically associating polymer and its adaptability to the oil reservoir. Pet. Geol. Oilfield Dev. Daqing.

[138] Jiang J., Rui Z., Hazlett R., Lu J. (2019). An integrated technical-economic model for evaluating CO_2_ enhanced oil recovery development. Appl. Energy.

[139] Feng Y., Billon L., Grassl B., Bastiat G., Borisov O., François J. (2005). Hydrophobically associating polyacrylamides and their partially hydrolyzed derivatives prepared by post-modification. 2. Properties of non-hydrolyzed polymers in pure water and brine. Polymer.

[140] Feng Y., Billon L., Grassl B., Khoukh A., François J. (2002). Hydrophobically associating polyacrylamides and their partially hydrolyzed derivatives prepared by post-modification. 1. Synthesis and characterization. Polymer.

[141] Li X., Zhang J., Zheng X., CHEN H.l., MING H. (2013). Sealing characteristics of compound system of cross-link polymer microspheres and association polymer. Mod. Chem. Ind..

[142] Blencowe A., Tan J.F., Goh T.K., Qiao G.G. (2009). Core cross-linked star polymers via controlled radical polymerisation. Polymer.

[143] Iwashita J., Hirayama T., Takagi I., Matsuzawa K., Suzuki K., Yoshizawa S., Konno K., Yahagi M., Sato K., Tagawa S. (2011). Characteristics of main chain decomposable STAR polymer for EUV resist. Advances in Resist Materials and Processing Technology XXVIII.

[144] Wenli L., Dong H., Li W., Qingxia L., Jian F. (2010). Synthesis and property evaluation of a salt-and alkali-resistant star-polymer. Pet. Explor. Dev..

[145] Li X., Yin H.Y., Zhang R.S., Cui J., Wu J.W., Feng Y.J. (2019). A salt-induced viscosifying smart polymer for fracturing inter-salt shale oil reservoirs. Pet. Sci..

[146] Nasr S., Soudi M.R., Haghighi M. (2007). Xanthan production by a native strain of X. campestris and evaluation of application in EOR. Pak. J. Biol. Sci. PJBS.

[147] Seright R.S., Wavrik K.E., Zhang G., AlSofi A.M. (2021). Stability and behavior in carbonate cores for new enhanced-oil-recovery polymers at elevated temperatures in hard saline brines. SPE Reserv. Eval. Eng..

[148] Alquraishi A.A., Alsewailem F.D. (2012). Xanthan and guar polymer solutions for water shut off in high salinity reservoirs. Carbohydr. Polym..

[149] Rivenq R., Donche A., Nolk C. (1992). Improved scleroglucan for polymer flooding under harsh reservoir conditions. SPE Reserv. Eng..

[150] Kalpakci B., Jeans Y., Magri N., Padolewski J. (1990). Thermal stability of scleroglucan at realistic reservoir conditions. SPE/DOE Enhanced Oil Recovery Symposium.

[151] Zohuriaan M., Shokrolahi F. (2004). Thermal studies on natural and modified gums. Polym. Test..

[152] Xu L., Xu G., Liu T., Chen Y., Gong H. (2013). The comparison of rheological properties of aqueous welan gum and xanthan gum solutions. Carbohydr. Polym..

[153] Song H., Zhang S.F., Ma X.C., Wang D.Z., Yang J.Z. (2007). Synthesis and application of starch-graft-poly (AM-co-AMPS) by using a complex initiation system of CS-APS. Carbohydr. Polym..

[154] Sen R. (2008). Biotechnology in petroleum recovery: The microbial EOR. Prog. Energy Combust. Sci..

[155] Haruna M.A., Nourafkan E., Hu Z., Wen D. (2019). Improved polymer flooding in harsh environments by free-radical polymerization and the use of nanomaterials. Energy Fuels.

[156] Volpert E., Selb J., Candau F., Green N., Argillier J., Audibert A. (1998). Adsorption of hydrophobically associating polyacrylamides on clay. Langmuir.

[157] Chassenieux C., Nicolai T., Benyahia L. (2011). Rheology of associative polymer solutions. Curr. Opin. Colloid Interface Sci..

[158] Perttamo E.K. (2013). Characterization of Associating Polymer (AP) Solutions. Influences on Flow Behavior by the Degree of Hydrophobicity and Salinity. Master’s Thesis.

[159] Niu Y., Jian O., Zhu Z., Wang G., Sun G., Shi L. (2001). Research on hydrophobically associating water-soluble polymer used for EOR. SPE International Symposium on Oilfield Chemistry.

[160] Huang Y., Santore M.M. (2002). Dynamics in adsorbed layers of associative polymers in the limit of strong backbone- surface attractions. Langmuir.

[161] Tanaka R., Williams P., Meadows J., Phillips G. (1992). The adsorption of hydroxyethyl cellulose and hydrophobically modified hydroxyethyl cellulose onto polystyrene latex. Colloids Surfaces.

[162] Zhu Y., Lei M., Zhu Z. (2015). Development and performance of salt-resistant polymers for chemical flooding. SPE Middle East Oil & Gas Show and Conference.

[163] Leblanc T., Braun O., Thomas A., Divers T., Gaillard N., Favero C. (2015). Rheological properties of stimuli-responsive polymers in solution to improve the salinity and temperature performances of polymer-based chemical enhanced oil recovery technologies. SPE Asia Pacific Enhanced Oil Recovery Conference.

[164] Kamal M.S., Sultan A. (2017). Thermosensitive water soluble polymers: A solution to high temperature and high salinity reservoirs. SPE Kingdom of Saudi Arabia Annual Technical Symposium and Exhibition.

[165] Levitt D.B., Dufour S., Pope G., Morel D., Gauer P. (2012). Design of an ASP flood in a high-temperature, high-salinity, low-permeability carbonate. IPTC 2012: International Petroleum Technology Conference.

[166] Jensen T., Kadhum M., Kozlowicz B., Sumner E., Malsam J., Muhammed F., Ravikiran R. (2018). Chemical EOR under harsh conditions: Scleroglucan as a viable commercial solution. SPE Improved Oil Recovery Conference.

[167] Ouellette R., Rawn J., Ouellette R.J., Rawn J.D. (2015). 15-Synthetic Polymers. Principles of Organic Chemistry.

[168] Svec F., Tennikova T.B., Deyl Z. (2003). Monolithic Materials: Preparation, Properties and Applications.

[169] Dupuis G., Antignard S., Giovannetti B., Gaillard N., Jouenne S., Bourdarot G., Morel D., Zaitoun A. (2017). A New Thermally Stable Synthetic Polymer for Harsh Conditions of Middle East Reservoirs. Part I. Thermal Stability and Injection in Carbonate Cores. Abu Dhabi International Petroleum Exhibition & Conference.

[170] Alfazazi U., Thomas N.C., Alameri W., Al-Shalabi E.W. (2020). Experimental investigation of polymer injectivity and retention under harsh carbonate reservoir conditions. J. Pet. Sci. Eng..

[171] Sandengen K., Meldahl M., Gjersvold B., Molesworth P., Gaillard N., Braun O., Antignard S. (2018). Long term stability of ATBS type polymers for enhanced oil recovery. J. Pet. Sci. Eng..

[172] Rodriguez L., Antignard S., Giovannetti B., Dupuis G., Gaillard N., Jouenne S., Bourdarot G., Morel D., Zaitoun A., Grassl B. (2018). A new thermally stable synthetic polymer for harsh conditions of middle east reservoirs: Part II. Nmr and size exclusion chromatography to assess chemical and structural changes during thermal stability tests. SPE Improved Oil Recovery Conference.

[173] Driver J.W., Britton C., Hernandez R., Glushko D., Pope G.A., Delshad M. (2018). Conditioning Polymer Solutions for Injection into Tight Reservoir Rocks. SPE Improved Oil Recovery Conference.

[174] Zhu D., Wei L., Wang B., Feng Y. (2014). Aqueous hybrids of silica nanoparticles and hydrophobically associating hydrolyzed polyacrylamide used for EOR in high-temperature and high-salinity reservoirs. Energies.

[175] Bennetzen M.V., Gilani S.F., Mogensen K., Ghozali M., Bounoua N. (2014). Successful polymer flooding of low-permeability, oil-wet, carbonate reservoir cores. Abu Dhabi International Petroleum Exhibition and Conference.

[176] Masalmeh S., AlSumaiti A., Gaillard N., Daguerre F., Skauge T., Skuage A. (2019). Extending polymer flooding towards high-temperature and high-salinity carbonate reservoirs. Abu Dhabi International Petroleum Exhibition & Conference.

[177] Jouenne S., Chakibi H., Levitt D. (2015). Polymer Stability Following Successive Mechanical Degradation Events. Proceedings of the IOR 2015—18th European Symposium on Improved Oil Recovery.

[178] Sheng J.J. (2014). Critical review of low-salinity waterflooding. J. Pet. Sci. Eng..

[179] Wang J., Han M., Fuseni A.B., Cao D. (2015). Surfactant adsorption in surfactant-polymer flooding for carbonate reservoirs. SPE Middle East Oil & Gas Show and Conference.

[180] Wang L., Mohanty K. (2015). Enhanced oil recovery in gasflooded carbonate reservoirs by wettability-altering surfactants. SPE J..

[181] Abalkhail N., Liyanage P.J., Upamali K.A., Pope G.A., Mohanty K.K. (2020). Alkaline-surfactant-polymer formulation development for a HTHS carbonate reservoir. J. Pet. Sci. Eng..

[182] Pourafshary P., Moradpour N. (2019). Hybrid EOR methods utilizing low-salinity water. Enhanc. Oil Recov. Process. New Technol.

[183] Dong X., Liu H., Chen Z. (2021). Introduction to hybrid enhanced oil recovery processes. Developments in Petroleum Science.

[184] Al-Murayri M.T., Kamal D.S., Al-Sabah H.M., AbdulSalam T., Al-Shamali A., Quttainah R., Glushko D., Britton C., Delshad M., Liyanage J. (2019). Low-Salinity Polymer Flooding in a High-Temperature Low-Permeability Carbonate Reservoir in West Kuwait. SPE Kuwait Oil & Gas Show and Conference.

[185] Lee Y., Lee W., Jang Y., Sung W. (2019). Oil recovery by low-salinity polymer flooding in carbonate oil reservoirs. J. Pet. Sci. Eng..

[186] Zhao Y., Yin S., Seright R.S., Ning S., Zhang Y., Bai B. (2021). Enhancing heavy-oil-recovery efficiency by combining low-salinity-water and polymer flooding. SPE J..

[187] Yan W., Miller C.A., Hirasaki G.J. (2006). Foam sweep in fractures for enhanced oil recovery. Colloids Surfaces A Physicochem. Eng. Asp..

[188] Heller J., Dandge D., Card R., Donaruma L. (1985). Direct thickeners for mobility control of CO2 floods. Soc. Pet. Eng. J..

[189] Horgue P., Soulaine C., Franc J., Guibert R., Debenest G. (2015). An open-source toolbox for multiphase flow in porous media. Comput. Phys. Commun..

[190] Chakravarthy D., Muralidharan V., Putra E., Hidayati D., Schechter D.S. (2006). Mitigating oil bypassed in fractured cores during CO_2_ flooding using WAG and polymer gel injections. SPE/DOE Symposium on Improved oil Recovery.

[191] Li W., Dong Z., Sun J., Schechter D.S. (2014). Polymer-alternating-gas simulation—A case study. SPE EOR Conference at Oil and Gas West Asia.

[192] Smith D.H. (1988). Surfactant-Based Mobility Control.

[193] Hiraski G.J. (1989). The steam-foam process. J. Pet. Technol..

[194] Zhang Y., Yue X., Dong J., Yu L. (2000). New and effective foam flooding to recover oil in heterogeneous reservoir. SPE/DOE Improved Oil Recovery Symposium.

[195] Ransohoff T., Radke C. (1988). Mechanisms of foam generation in glass-bead packs. SPE Reserv. Eng..

[196] Rossen W., Gauglitz P. (1990). Percolation theory of creation and mobilization of foams in porous media. AIChE J..

[197] Gauglitz P.A., Friedmann F., Kam S.I., Rossen W. (2002). Foam generation in porous media. SPE/DOE Improved Oil Recovery Symposium.

[198] Dicksen T., Hirasaki G.J., Miller C.A. (2002). Conditions for foam generation in homogeneous porous media. SPE/DOE Improved oil Recovery Symposium.

[199] Donaldson E.C., Chilingarian G.V., Yen T.F. (1989). Enhanced Oil Recovery, II: Processes and Operations.

[200] Heller J.P. (1994). CO_2_ Foams in Enhanced Oil Recovery.

[201] Lal R., Negassa W., Lorenz K. (2015). Carbon sequestration in soil. Curr. Opin. Environ. Sustain..

[202] Wei Z., Jian Z., Ming H., Wentao X., Guozhi F., Wei J., Fujie S., Shouwei Z., Yongjun G., Zhongbin Y. (2007). Application of hydrophobically associating water-soluble polymer for polymer flooding in China offshore heavy oilfield. IPTC 2007: International Petroleum Technology Conference.

[203] Jiran L. (2015). Experimental Investigation and Simulation of Polymer Flooding in High Temperature High Salinity Carbonate Reservoirs. Ph.D. Thesis.

[204] Jabbar M., Xiao R., Teletzke G.F., Willingham T., Al Obeidli A., Al Sowaidi A., Britton C., Delshad M., Li Z. (2018). Polymer EOR Assessment through Integrated Laboratory and Simulation Evaluation for an Offshore Middle East Carbonate Reservoir. Abu Dhabi International Petroleum Exhibition & Conference.

[205] Mohsenatabar Firozjaii A., Akbari M., Zargar G. (2019). Sensitivity analysis and optimization on effective parameters during chemical enhanced oil recovery (CEOR) using experimental design and numerical simulation. Energy Sources Part A Recover. Util. Environ. Eff..

[206] Rachapudi R., Alshehhi S., BinAmro A.A., Masalmeh S., Dey A., Al Nuimi S., Kenawy M., Fabbri C., Romero C., Xu S. (2020). World First Polymer Injectivity Test in High Salinity and High Temperature Carbonate Reservoir, Case Study from a Giant Reservoir in UAE. Abu Dhabi International Petroleum Exhibition & Conference.

[207] Baloch S., Leon J., Masalmeh S., Chappell D., Brodie J., Romero C., Al Mazrouei S., Al Tenaiji A., Al Balooshi M., Igogo A. (2021). Expanding Polymer Injectivity Tests on a Second Giant Carbonate UAE Oil Reservoir at High Salinity & High Temperature Conditions. Abu Dhabi International Petroleum Exhibition & Conference.

[208] Manrique E., Thomas C., Ravikiran R., Izadi M., Lantz M., Romero J., Alvarado V. (2010). EOR: Current status and opportunities. SPE Improved oil Recovery Symposium.

[209] Ghosh P., Sharma H., Mohanty K.K. (2017). Chemical Flooding in Low Permeability Carbonate Rocks. SPE Annual Technical Conference and Exhibition.

[210] Moore J.K. (1969). Reservoir barrier and polymer waterflood, Northeast Hallsville Crane unit. J. Pet. Technol..

[211] Tiwari D., Marathe R.V., Patel N.K., Ramachandran K., Maurya C.R., Tewari P. (2008). Performance of polymer flood in Sanand field, India-A case study. SPE Asia Pacific Oil and Gas Conference and Exhibition.

[212] Masalmeh S., Wei L., Hillgartner H., Al-Mjeni R., Blom C. (2012). Developing high resolution static and dynamic models for waterflood history matching and EOR evaluation of a Middle Eastern carbonate reservoir. Abu Dhabi International Petroleum Conference and Exhibition.

[213] Leon J.M., Masalmeh S.K., Xu S., AlSumaiti A.M., BinAmro A.A., Baslaib M.A. (2021). Analysis of the World’s First Polymer Injectivity Test in a Carbonate Reservoir Under Extreme Harsh Conditions in ADNOC’s Reservoirs. Abu Dhabi International Petroleum Exhibition & Conference.

[214] Al-Shalabi E.W. (2018). Numerical modeling of biopolymer flooding in high-temperature high-salinity carbonate cores. Offshore Technology Conference Asia.

[215] Rai S.K., Bera A., Mandal A. (2015). Modeling of surfactant and surfactant–polymer flooding for enhanced oil recovery using STARS (CMG) software. J. Pet. Explor. Prod. Technol..

[216] Rivet S.M. (2009). Coreflooding Oil Displacements with Low Salinity Brine. Ph.D. Thesis.

[217] Ayirala S., Uehara-Nagamine E., Matzakos A., Chin R., Doe P., van Den Hoek P. (2010). A designer water process for offshore low salinity and polymer flooding applications. SPE Improved Oil Recovery Symposium.

[218] Alzayer H., Sohrabi M. (2013). Numerical simulation of improved heavy oil recovery by low-salinity water injection and polymer flooding. SPE Saudi Arabia Section Technical Symposium and Exhibition.

[219] Webb K., Lager A., Black C. Comparison of high/low salinity water/oil relative permeability. Proceedings of the International Symposium of the Society of Core Analysts.

[220] Hirasaki G., Pope G. (1974). Analysis of factors influencing mobility and adsorption in the flow of polymer solution through porous media. Soc. Pet. Eng. J..

[221] Han B., Lee J. (2014). Sensitivity analysis on the design parameters of enhanced oil recovery by polymer flooding with low salinity waterflooding. The Twenty-Fourth International Ocean and Polar Engineering Conference.

[222] Khamees T.K., Flori R.E., Alsubaih A.A., Alhuraishawy A.K. (2014). Modeling the effects of salinity, polymer rheology, temperature, and reservoir wettability on the performance of in-depth gel treatment coupled with surfactant and polymer flooding. Abu Dhabi International Petroleum Exhibition & Conference.

[223] Ning S., Barnes J., Edwards R., Dunford K., Eastham K., Dandekar A., Zhang Y., Cercone D., Ciferno J. (2019). First Ever Polymer Flood Field Pilot to Enhance the Recovery of Heavy Oils on Alaska’s North Slope—Polymer Injection Performance. Proceedings of the Unconventional Resources Technology Conference.

[224] Zhao Y., Yin S., Seright R.S., Ning S., Zhang Y., Bai B. (2020). Performance of Low Salinity Polymer Flood in Enhancing Heavy Oil Recovery on the Alaska North Slope. Proceedings of the Unconventional Resources Technology Conference.

[225] Wang D., Li C., Seright R.S. (2020). Laboratory Evaluation of Polymer Retention in a Heavy Oil Sand for a Polymer Flooding Application on Alaska’s North Slope. SPE J..

[226] Sun X.F., Song Z.Y., Cai L.F., Zhang Y.Y., Li P. (2020). Phase behavior of heavy oil–solvent mixture systems under reservoir conditions. Pet. Sci..

[227] Kargozarfard Z., Riazi M., Ayatollahi S. (2019). Viscous fingering and its effect on areal sweep efficiency during waterflooding: An experimental study. Pet. Sci..

[228] Saboorian-Jooybari H., Dejam M., Chen Z. (2016). Heavy oil polymer flooding from laboratory core floods to pilot tests and field applications: Half-century studies. J. Pet. Sci. Eng..

[229] Dandekar A., Bai B., Barnes J., Cercone D., Ciferno J., Ning S., Seright R., Sheets B., Wang D., Zhang Y. (2019). First Ever Polymer Flood Field Pilot-A Game Changer to Enhance the Recovery of Heavy Oils on Alaska’s North Slope. SPE Western Regional Meeting.

[230] Dandekar A., Bai B., Barnes J., Cercone D., Ciferno J., Edwards R., Ning S., Schulpen W., Seright R., Sheets B. (2021). First ever polymer flood field pilot to enhance the recovery of heavy oils on Alaska’s North Slope Pushing Ahead One Year Later. SPE Western Regional Meeting.

[231] Al Shalabi E.W., Sepehrnoori K. (2017). Low Salinity and Engineered Water Injection for Sandstone and Carbonate Reservoirs.

[232] Al-Shalabi E.W., Sepehrnoori K., Pope G. (2016). Numerical modeling of combined low salinity water and carbon dioxide in carbonate cores. J. Pet. Sci. Eng..

[233] Al-Shalabi E.W., Sepehrnoori K., Delshad M., Pope G. (2014). A Novel Method to Model Low Salinity Water Injection in Carbonate Oil Reservoirs. SPE EOR Conference at Oil and Gas West Asia.

[234] Al-Shalabi E.W., Sepehrnoori K., Pope G. (2015). New mobility ratio definition for estimating volumetric sweep efficiency of low salinity water injection. Fuel.

[235] Al-Shalabi E.W., Sepehrnoori K. (2016). A comprehensive review of low salinity/engineered water injections and their applications in sandstone and carbonate rocks. J. Pet. Sci. Eng..

[236] Fortenberry R., Kim D.H., Nizamidin N., Adkins S., Pinnawala Arachchilage G.W., Koh H., Weerasooriya U., Pope G.A. (2015). Use of cosolvents to improve alkaline/polymer flooding. SPE J..

